# Three-component reaction of azulene, aryl glyoxal and 1,3-dicarbonyl compound for the synthesis of various azulene derivatives†

**DOI:** 10.1039/d0ra00356e

**Published:** 2020-03-10

**Authors:** Jing Gong, Anatoly A. Peshkov, Jiafeng Yu, Sagadat Amandykova, Aidana Gimnkhan, Jianjun Huang, Stepan Kashtanov, Olga P. Pereshivko, Vsevolod A. Peshkov

**Affiliations:** College of Chemistry, Chemical Engineering and Materials Science, Soochow University Dushu Lake Campus Suzhou 215123 P. R. China olga@suda.edu.cn vsevolod@suda.edu.cn; Department of Chemistry, School of Sciences and Humanities, Nazarbayev University 53 Kabanbay Batyr Ave, Block 7 Nur-Sultan 010000 Republic of Kazakhstan, olga.pereshivko@nu.edu.kz vsevolod.peshkov@nu.edu.kz; Department of Chemistry, Xi'an Jiaotong-Liverpool University Suzhou 215123 P. R. China; The Environment and Resource Efficiency Cluster (EREC), Nazarbayev University Nur-Sultan Republic of Kazakhstan

## Abstract

A three-component reaction of an azulene, an aryl glyoxal and a 1,3-dicarbonyl compound has been elaborated to access a series of azulene derivatives. Some of these azulene-containing adducts were further subjected to post-MCR transformations to assemble azulene–heterocycle conjugates.

## Abstract 2

Three-component reaction of azulene, aryl glyoxal and 1,3-dicarbonyl compound and subsequent post-transformations provide access to three distinct types of azulene derivatives.

## Graphical abstract

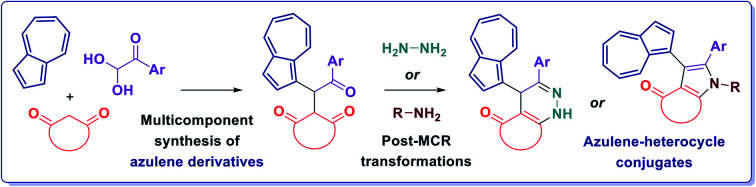

## Introduction

Azulene is a bicyclic aromatic hydrocarbon with a deep blue colour and a dipole moment of about 1.08 D.^1^ Such properties are in striking contrast with those of the isomeric naphthalene that is colourless and has a dipole moment of 0 D. The polarity of azulene and in turn the appearance of the blue colour can be explained by the charge-separated resonance structure in which the bicyclic core of azulene is regarded as a fusion of 6 π-electron cyclopentadienyl anion and 6 π-electron tropylium cation.

Owing to the unique structural and photophysical properties of the azulene core, a number of azulene-based advanced organic materials^2^ has been developed targeting the applications in sensors,^3^ bioimaging,^4^ non-linear optics (NLO),^5^ optoelectronics,^6^ molecular electronics^7^ and so on. Furthermore, azulene derivatives have been successfully incorporated in solar cells^8^ and organic field-effect transistors (OFETs)^8*c*,*d*,9^ demonstrating high potential for further exploration in this type of devices.

Consequently, this sparked a growing interest in the development of novel synthetic methodologies for azulene construction^10^ and functionalization^11^ with a special emphasis being given to the assembly of azulene-fused heterocycles,^12^ azulene–heterocycle conjugates^13^ and azulene-containing polymers.^8*a*,*c*,*d*,14^

Several recent methodologies for azulene functionalization involve one-pot and/or multicomponent approaches.^15^ On the other hand, in recent years, a number of multicomponent transformations have been developed based on the ability of aryl glyoxals to react with 1,3-dicarbonyl compounds and additional nucleophiles resulting in the formation of structurally diverse (heterocyclic) adducts.^16^ We decided to take an advantage of this strategy towards the synthesis of azulene derivatives through exploration of the nucleophilic potential of the five-membered ring of azulene core.

## Results and discussion

**Scheme 1:**
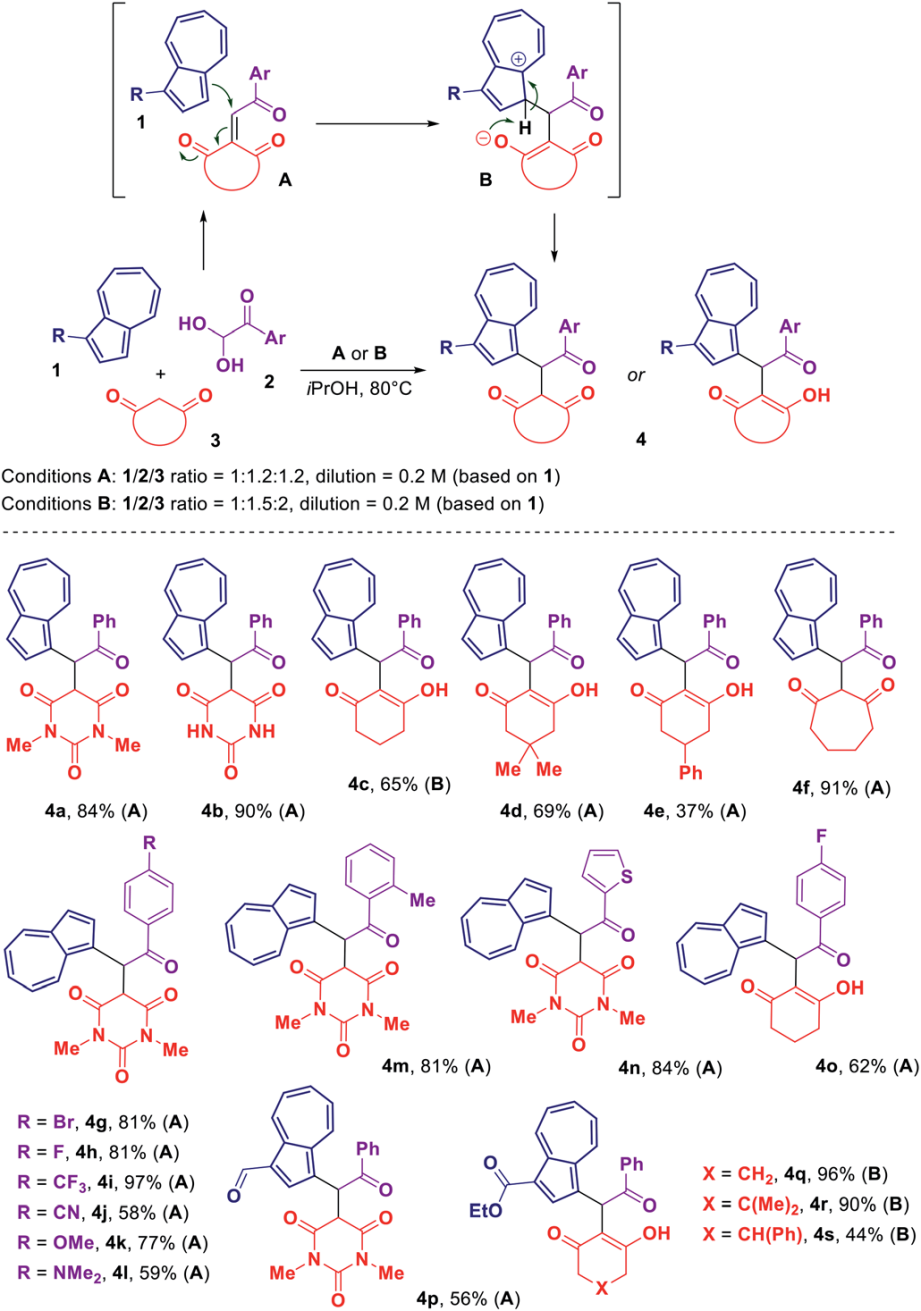
Scope of the three-component reaction of azulene 1, aryl glyoxal monohydrate 2 and 1,3-dicarbonyl compound 3.

Knowing that the treatment of an aryl glyoxal 2 with a 1,3-dicarbonyl compound 3 results in the Knoevenagel condensation,^16^ we envisaged that the presence of an azulene 1 would trigger the Michael addition of 1 onto the Knoevenagel adduct A. A subsequent proton transfer in the intermediate B would produce the desired azulene derivative 4 (Scheme 1). After conducting a brief screening of the reaction conditions (see ESI†), we were pleased to find that such a three-component transformation could be successfully accomplished at the elevated temperature of 80 °C using isopropanol as a solvent.

The scope of the resulting process is outlined in Scheme 1. In order to evaluate the reactivity of a 1,3-dicarbonyl component 3, several barbituric acid derivatives and cyclic 1,3-diketones were reacted with unsubstituted azulene and phenyl glyoxal monohydrate resulting in the formation of products 4a–f with the yields ranging from 37% to 91%. Interestingly, according to NMR analysis, products 4a, b, f derived from either barbituric acids or cycloheptane-1,3-dione were observed in a keto form in solution (CDCl_3_ for 4a and 4f, [D_6_]DMSO for 4b). In contrast, products 4c–e obtained using various cyclohexane-1,3-diones were observed in a enolized form, with the compound 4e existing as a mixture of two interconvertible diastereomeric enol forms.

With respect to an aryl glyoxal component 2, a number of variously substituted phenyl glyoxal monohydrates along with a heteroaromatic thiophen-2-yl glyoxal monohydrate have been tested allowing to acquire an array of azulene-containing adducts 4g–o (Scheme 1). It was found that the presence of either electron-withdrawing or electron-donating substituent in the phenyl ring of glyoxal could be well tolerated.

Blocking one of the azulene's reactive positions with an electron-withdrawing group did not shut down the reactivity of the azulene core towards our transformation. Thus, we were able to prepare a series of 1,3-disubstituted azulene derivatives 4p–s starting from either azulene-1-carbaldehyde or ethyl azulene-1-carboxylate.

**Scheme 2:**
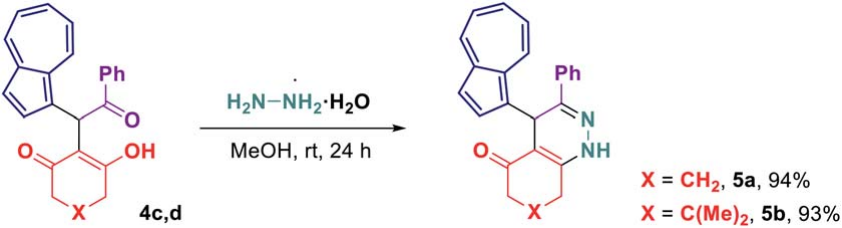
Synthesis of azulene–tetrahydrocinnolin-5-one conjugates 5.

**Scheme 3:**
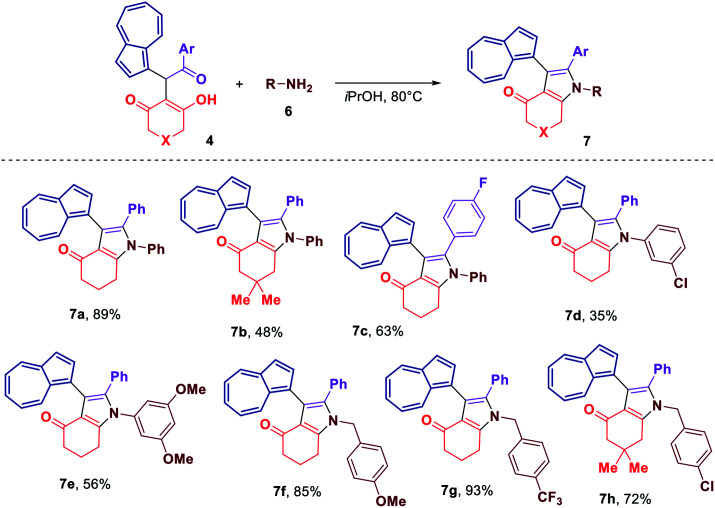
Synthesis of azulene–dihydroindol-4-one conjugates 7.

**Fig. 1:**
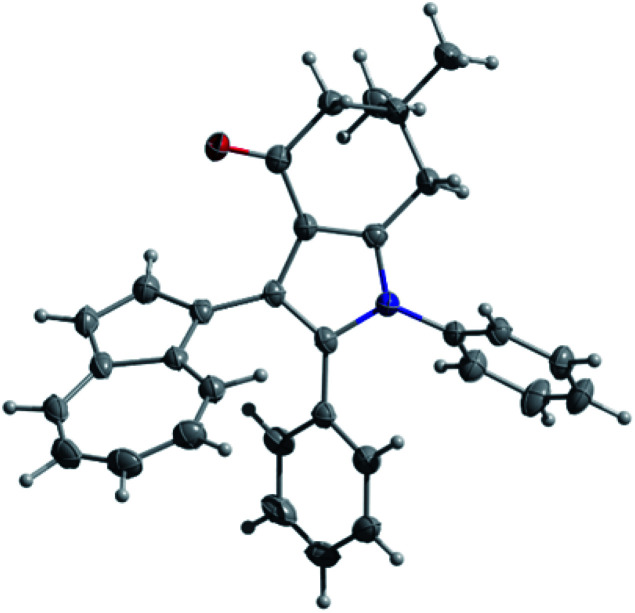
Molecular structure of 7b, showing thermal displacement ellipsoids at the 50% probability level. The dimethyl formamide (DMF) molecule acquired during the crystallization process and present in the crystal packing is not shown.

Considering that some of the obtained azulene derivatives, such as for example 4c and 4d comprised a 1,4-diketo unit, we decided to probe their reactivity in the condensations with nitrogen nucleophiles towards the formation of azulene–heterocycle conjugates. To our delight, reacting 4c and 4d with hydrazine monohydrate in methanol at rt produced azulene–tetrahydrocinnoline conjugates 5a and 5b in high yields of 94% and 93%, respectively (Scheme 2). Encouraged by these results, we went on exploring the potential of our 1,4-diketones in a Paal–Knorr synthesis of pyrroles.^17^ Gratifyingly, the treatment of 4c, 4d and 4o with aniline in isopropanol at 80 °C allowed to prepare azulene–dihydroindol-4-one conjugates 7a–c in moderate to good yields (Scheme 3). The molecular structure of representative azulene–dihydroindol-4-one derivative 7b has been resolved through the X-ray crystallographic analysis (Fig. 1, see ESI† for details). The above synthetic strategy was also found to be amenable to a variation of an amine component 6. Examining different aromatic and benzylic amines in the reactions with 4c or 4d delivered expected azulene-substituted dihydroindol-4-ones 7c–h in up to 93% yield (Scheme 3).

**Scheme 4:**
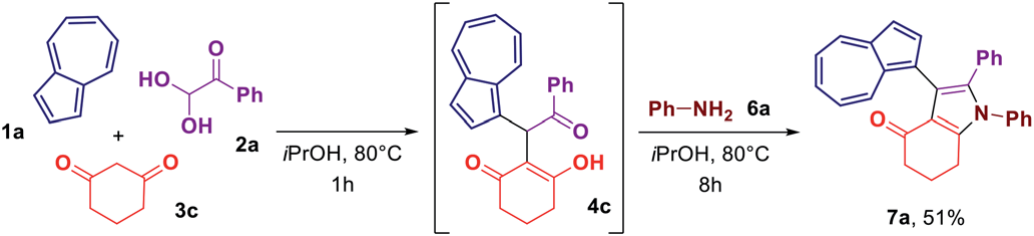
One-pot synthesis of azulene–dihydroindol-4-one conjugate 7a.

In an attempt to streamline the access towards azulene–heterocycle conjugates, we have conducted a one-pot synthesis of compounds 7a (Scheme 4). Reacting azulene (1a), phenyl glyoxal monohydrate (2a) and cyclohexane-1,3-dione (3c) in isopropanol at 80 °C for 1 h lead to the formation of acyclic adduct 4c. Once the formation of 4c was confirmed by the TLC analysis, the aniline (6a) was added and the reaction was continued for another 8 h allowing to obtain the desired azulene-substituted dihydroindol-4-one 7a in 51% overall yield.

**Fig. 2:**
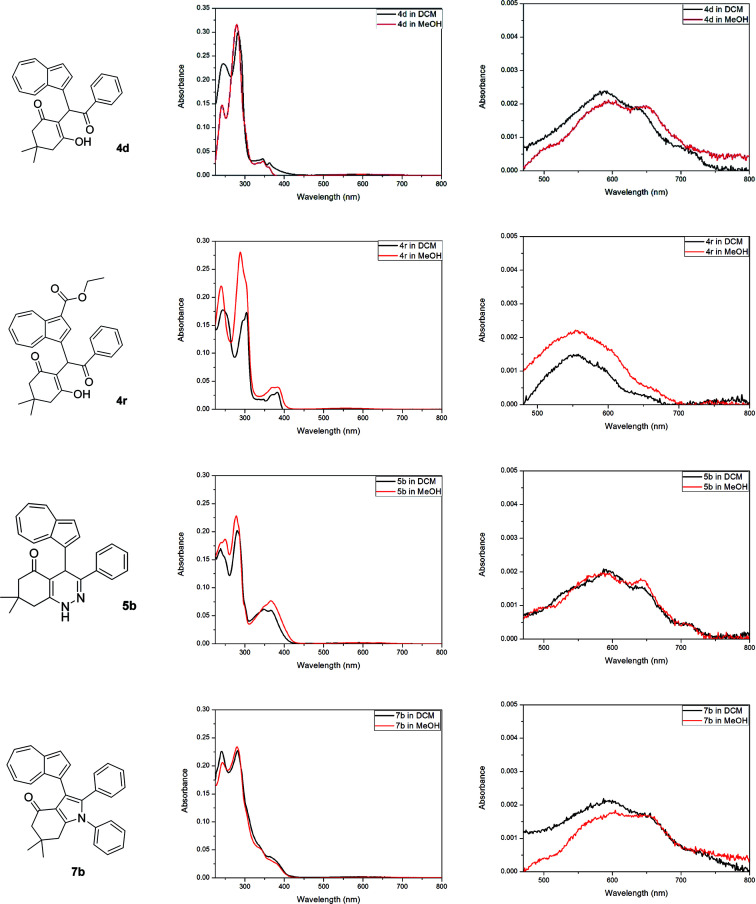
UV/Vis absorption spectra of 4d, 4r, 5b and 7b measured in dichloromethane and in methanol (both at *c* ≅ 5 × 10^−6^ M, left column); magnified visible region of UV/Vis absorption spectra of 4d, 4r, 5b and 7b (right column).

The optical properties of all acquired azulene derivatives 4, 5 and 7 have been assessed by measuring their UV/Vis absorption in dichloromethane and in methanol (both at *c* ≅ 5 × 10^−6^ M, see ESI†).‡‡UV/Vis absorption of 7b was measured only in methanol due to poor solubility in dichloromethane. The UV/Vis absorption spectra of representative azulene-containing products 4d, 4r, 5b and 7b are shown in Fig. 2. Similarly to most of simple azulene derivatives, all prepared compounds 4, 5 and 7 were characterized by a strong absorbance in the UV region and a relatively weak absorbance in the visible region, with the latter being responsible for the colouration of their solutions.

## Conclusions

In conclusion, we have developed a novel multicomponent protocol for the azulene derivatization through the reaction with an aryl glyoxal and a 1,3-dicarbonyl compound. The scope of the process has been briefly explored resulting in generation of a small set of branched azulene-containing adducts. Some of these adducts could be further upgraded into azulene–heterocycle conjugates through the post-MCR condensations with nitrogen nucleophiles. Collectively, these methodologies provide a straightforward access to three distinct types of azulene derivatives.

## Conflicts of interest

There are no conflicts to declare.

## Supplementary Material

RA-010-D0RA00356E-s001

RA-010-D0RA00356E-s002
