# Supplementary material for: Three-component reaction of azulene, aryl glyoxal and 1,3-dicarbonyl compound for the synthesis of various azulene derivatives
Source: RSC Adv. 2020 Mar 10;10(17):10113–7. doi: 10.1039/d0ra00356e (PMC9050217; doi:10.1039/d0ra00356e)
Supplement: RA-010-D0RA00356E-s001 [file RA-010-D0RA00356E-s001.pdf]

Electronic Supplementary Material (ESI) for RSC Advances.
This journal is © The Royal Society of Chemistry 2020

Three-component reaction of azulene, aryl glyoxal and 1,3-dicarbonyl compound for the synthesis of various azulene derivatives

Gong Jing,[a] Anatoly A. Peshkov,[b] Jiafeng Yu,[a] Sagadat Amandykova,[b] Aidana Gimnkhan,[b] Jianjun Huang,[a] Stepan Kashtanov,[c] Olga P. Pereshivko*[,a,b] and Vsevolod A. Peshkov*[,a,b,d]

[a.] *College of Chemistry, Chemical Engineering and Materials Science, Soochow University, Dushu Lake Campus, Suzhou, 215123, P.R. China. email: olga@suda.edu.cn; vsevolod@suda.edu.cn*

[b.] *Department of Chemistry, School of Sciences and Humanities, Nazarbayev University, 53 Kabanbay Batyr Ave, Block 7, Nur-Sultan 010000, Republic of Kazakhstan. email: olga.pereshivko@nu.edu.kz; vsevolod.peshkov@nu.edu.kz*

[c.] *Department of Chemistry, Xi'an Jiaotong-Liverpool University, Suzhou, 215123, P.R. China.*

[d.] *The Environment and Resource Efficiency Cluster (EREC), Nazarbayev University, Nur-Sultan, Republic of Kazakhstan.*

Table of content

**This page**	**1**
**X-ray crystallographic analysis**	**2**
**Synthesis and characterization**	**6**
**UV/vis absorption spectra**	**20**
**Copies of ^{1}H and ^{13}C NMR spectra**	**28-85**

X-ray crystallographic analysis

General details

Single crystals of **7b** were obtained by slow evaporation from dimethylformamide. The images were interpreted and integrated with the program Diamond v.4.0 (Crystal Impact).[1] Using Olex2,[2] the structures were solved with the ShelXS[3] structure solution program using direct methods and refined by full-matrix least-squares on F^2 with the ShelXL[4] refinement package.

Crystal data for 7b

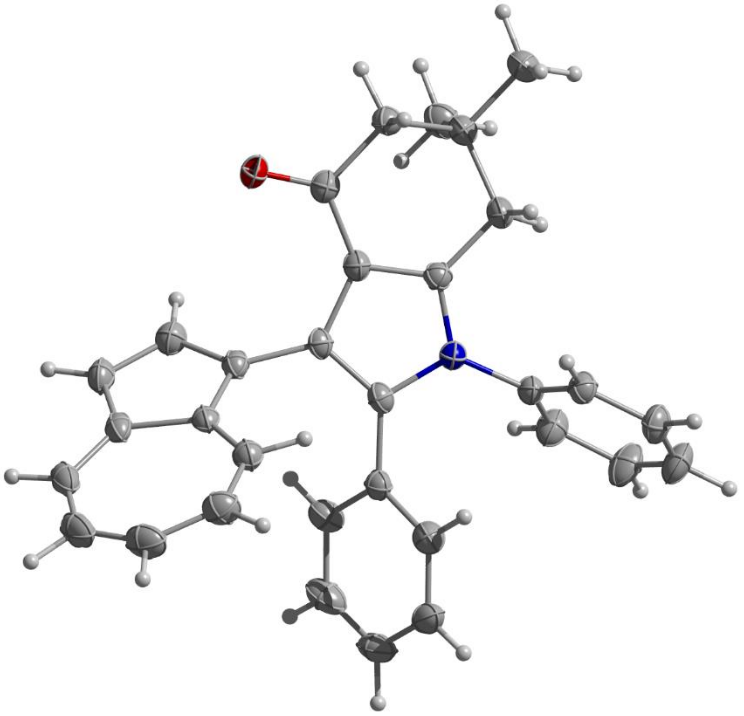

Molecular structure of **7b**, showing thermal displacement ellipsoids at the 50% probability level; the dimethyl formamide (DMF) molecule acquired during the crystallization process and present in the crystal packing is not shown

Crystal data for 7b·DMF. $C_{32}H_{27}NO{\cdot}C_3H_7NO$, M = 514.64 g/mol, triclinic, space group P-1 (no. 2), a =

---

[1] Diamond - Crystal and Molecular Structure Visualization Crystal Impact - Dr. H. Putz & Dr. K. Brandenburg GbR, Kreuzherrenstr. 102, 53227 Bonn, Germany http://www.crystalimpact.com/diamond.

[2] O. V. Dolomanov, L. J. Bourhis, R. J. Gildea, J. A. K. Howard and H. Puschmann, *J. Appl. Cryst.*, 2009, **42**, 339.

[3] G. M. Sheldrick, *Acta Cryst. A*, 2008, **64**, 112.

[4] G. M. Sheldrick, *Acta Cryst. C*, 2015, **71**, 3.

10.9118(6) Å, b = 11.6167(6) Å, c = 13.1533(7) Å, V = 1363.93(13) $Å^3$, Z = 27, T = 296.15 K, μ(MoKα) = 0.077 mm^{-1}, $Dcalc$ = 1.253 g/cm^3, 64042 reflections measured (4.878° ≤ 2Θ ≤ 55.058°), 6259 unique (R_{int} = 0.0882, R_{sigma} = 0.0494) which were used in all calculations. The final R_1 was 0.0450 (I > 2σ(I)) and wR_2 was 0.1000 (all data).

Structural features:

- the unit cell contains two molecules of the compound ($C_{32}H_{27}NO$) along with two molecules of solvent (C_3H_7NO, dimethylformamide (DMF))

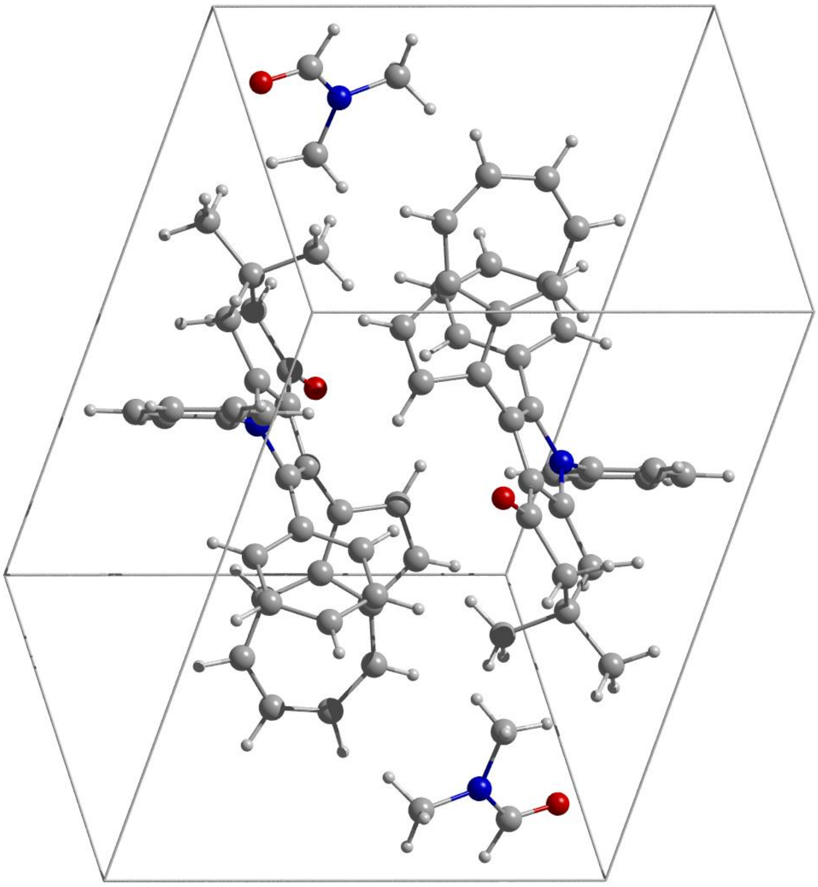

Composition of the unit cell of **7b**

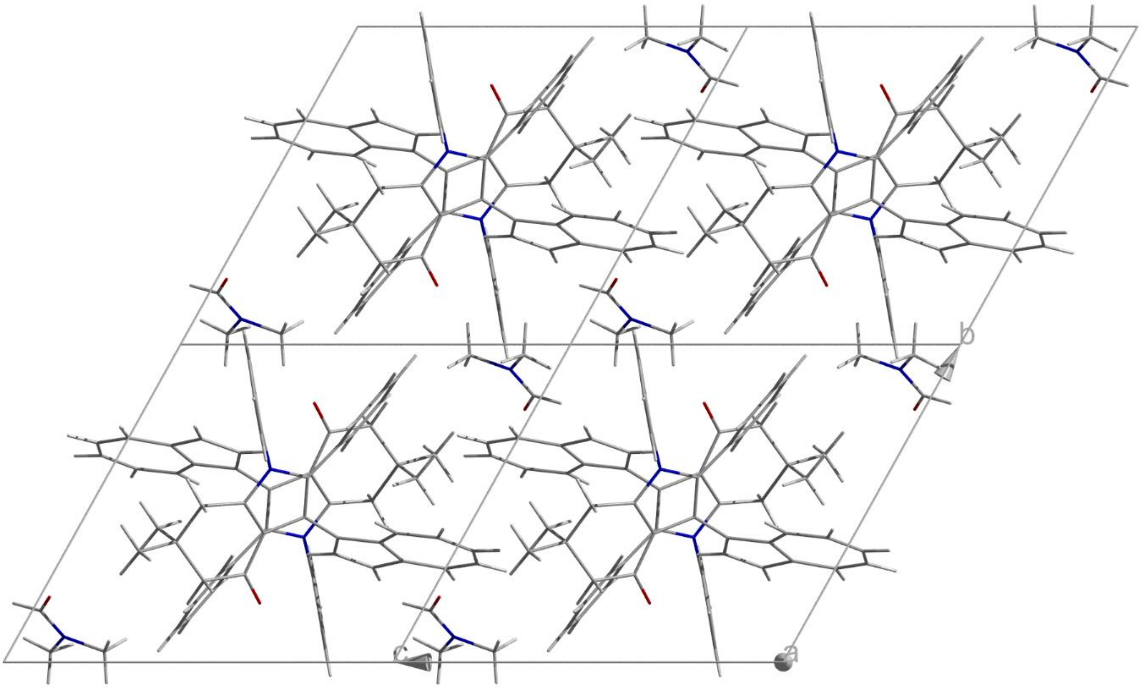

Packing diagram of the crystal structure of **7b**, viewed down the crystallographic a-axis.

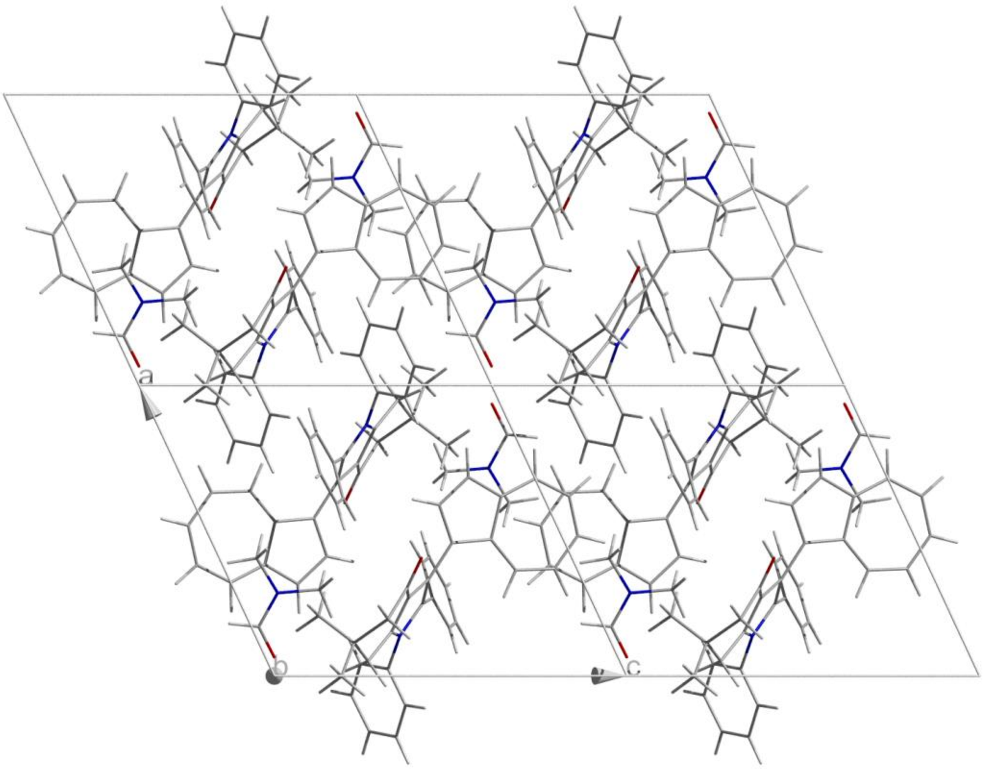

Packing diagram of the crystal structure of **7b**, viewed down the crystallographic b-axis.

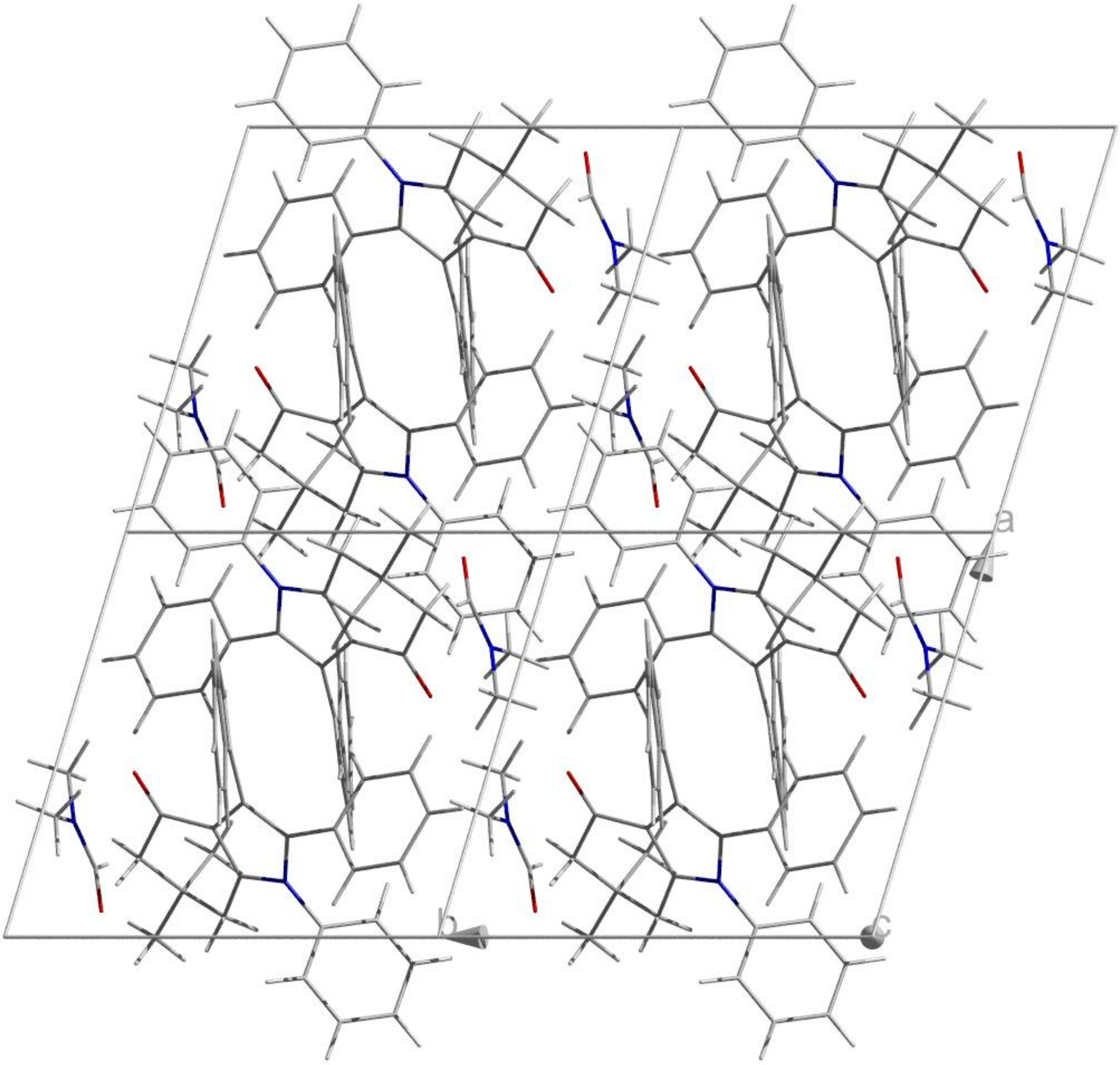

Packing diagram of the crystal structure of **7b**, viewed down the crystallographic c-axis.

Synthesis and characterization

General information

Unless otherwise specified, the starting materials and solvents were purchased from commercial sources and used as received. Azulene-1-carbaldehyde[5] and ethyl azulene-1-carboxylate[6] were synthesized following previously described protocols. Aryl glyoxal hydrates **2** were purchased or synthesized following previously described protocol.[7] Melting points were measured using INESA WRR apparatus. NMR spectra were recorded using 400 MHz Bruker Avance instruments. The ^{1}H and ^{13}C chemical shifts are reported relative to TMS using the residual $CDCl_3$ or $[D_6]DMSO$ signal as internal reference. HRMS were performed on a Bruker microTOF-Q III. UV-visible absorption spectra were recorded using Agilent Technologies Cary Series UV-VIS-NIR Spectrophotometer.

Screening of the reaction conditions for the synthesis of azulene derivative 4a

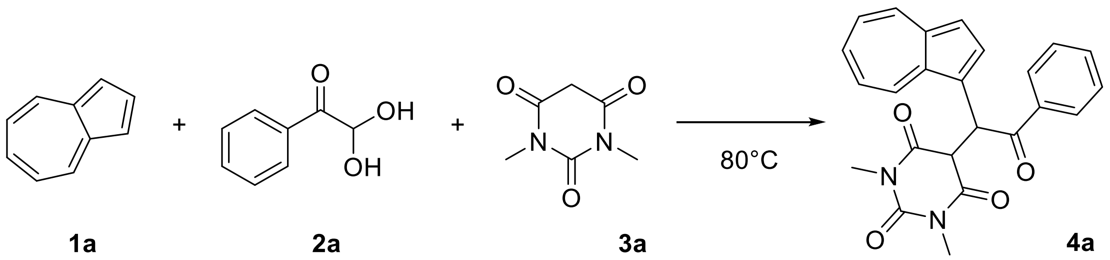

Entry	**1a**:**2a**:**3a** ratio	Solvent	Time (h)	Isolated yield (%)
1	1:1:1	MeOH	1	63
2	1:1:1	*i*PrOH	1	74
3	1:1.2:1.2	*i*PrOH	1	78
4	1:1.2:1.2	*i*PrOH	0.5	84
5	1:1:1	DMF	0.5	58
6	1:1:1	*t*BuOH	0.5	76
7	1:1:1	CF_3CH_2OH	0.5	-

5 A. Székely, Á. Péter, K. Aradi, G. L. Tolnai and Z. Novák, *Org. Lett.*, 2017, **19**, 954.

6 J. Dubovik and A. Bredihhin, *Synthesis*, 2015, **47**, 538.

7 (a) P. Wang, W.-J. Tao, X.-L. Sun, S. Liao and Y. Tang, *J. Am. Chem. Soc.*, 2013, **135**, 16849; (b) H. Batchu and S. Batra, *Eur. J. Org. Chem.*, 2012, 2935; (c) G.-X. He, J.-M. Yuan, H.-M. Zhu, K. Wei, L.-Y. Wang, S.-L. Kong, D.-L. Mo, C.-X. Pan and G.-F. Su, *Bioorg. Med. Chem. Lett.*, 2017, **27**, 1660; (d) G. Fodor and Ö. Kovacs, *J. Am. Chem. Soc.*, 1949, **71**, 1045.

Synthesis of azulene derivatives 4 *via* three-component reaction of azulene 1, aryl glyoxal 2 and 1,3-dicarbonyl compound 3

General procedure A

Arylglyoxal monohydrate **2** (0.48 mmol, 1.2 equiv) was placed in a screw-capped vial and dissolved in *i*PrOH (2 mL) followed by addition of azulene **1** (0.4 mmol, 1 equiv) and 1,3-dicarbonyl compound **3** (0.48 mmol, 1.2 equiv). The resulting mixture was sealed, placed in an oil bath preheated at 80 °C and stirred for 30 min. After completion of reaction, the resulting mixture was diluted with ethyl acetate. Then silica gel was added and the volatiles were removed under reduced pressure. Column chromatography with petroleum ether/EtOAc (the ratio was adjusted according to TLC) as eluent delivered azulene derivative **4**.

General procedure B

Arylglyoxal monohydrate **2** (0.3 mmol, 1.5 equiv) was placed in a screw-capped vial and dissolved in *i*PrOH (1 mL) followed by addition of azulene **1** (0.2 mmol, 1 equiv) and 1,3-dicarbonyl compound **3** (0.4 mmol, 2 equiv). The resulting mixture was sealed, placed in an oil bath preheated at 80 °C and stirred for 1h. Upon completion, the mixture was diluted with EtOAc and evaporated with silica gel under reduced pressure. Column chromatography with petroleum ether/EtOAc (the ratio was adjusted according to TLC) as eluent delivered azulene derivative **4**.

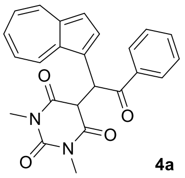

4a

5-(1-(Azulen-1-yl)-2-oxo-2-phenylethyl)-1,3-dimethylpyrimidine-2,4,6(1*H*,3*H*,5*H*)-trione (4a). Yield: 67 mg, 84% (Procedure A, 0.2 mmol scale); purple solid; mp: 151-152°C; ^{1}H NMR (400 MHz, $CDCl_3$) δ 8.54 (d, *J* = 9.5 Hz, 1H), 8.32 (d, *J* = 9.2 Hz, 1H), 7.80 – 7.55 (m, 4H), 7.47 – 7.15 (m, 6H), 6.46 (s, 1H), 3.84 (s, 1H), 3.33 (s, 3H), 3.32 (s, 3H); ^{13}C NMR (100 MHz, $CDCl_3$) δ 198.5, 168.1, 167.6, 151.7, 141.5, 140.7, 137.9, 137.2, 135.1, 134.9, 133.5, 131.8, 129.4, 128.6, 124.1, 123.7, 123.1, 117.5, 51.0, 50.4, 29.0, 28.7; HRMS (ESI, $[M+Na]^+$) for $C_{24}H_{20}N_2O_4Na^+$ calcd. 423.1315, found 423.1287.

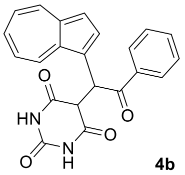

4b

5-(1-(Azulen-1-yl)-2-oxo-2-phenylethyl)pyrimidine-2,4,6(1*H*,3*H*,5*H*)-trione (4b). Yield: 134 mg, 90% (Procedure A, 0.4 mmol scale, column chromatography was conducted using CH_2Cl_2/MeOH (9:1) as eluent); dark grey-blue solid; mp: 151-153°C; ^{1}H NMR (400 MHz, [D$_6$]DMSO) δ 8.98 (s, 2H), 8.47 (d, *J* = 9.8 Hz, 1H), 8.24 (d, *J* = 9.2 Hz, 1H), 8.11 (d, *J* = 3.8 Hz, 1H), 7.98 – 7.90 (m, 2H), 7.58 – 7.43 (m,

2H), 7.41 – 7.34 (m, 2H), 7.29 (d, *J* = 3.8 Hz, 1H), 7.11 – 6.99 (m, 2H), 6.15 (s, 1H), 3.35 (s, 1H, tentatively overlaped with signal of water); ^{13}C NMR (100 MHz, [D$_6$]DMSO) δ 200.4, 163.6, 151.8, 140.4, 140.3, 138.4, 136.6, 135.2, 133.5, 131.4, 131.3, 127.7, 121.6, 121.0, 116.2, 89.3, 42.0, one signal is tentatively overlapped with the signal of [D$_6$]DMSO; HRMS (ESI, [M+Na]$^+$) for $C_{22}H_{16}N_2O_4Na^+$ calcd. 395.1002, found 395.1002.

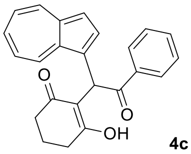

4c

2-(1-(Azulen-1-yl)-2-oxo-2-phenylethyl)-3-hydroxycyclohex-2-en-1-one (4c). Yield: 46 mg, 65% (Procedure B, 0.2 mmol scale); dark blue solid; mp: 80-82°C; ^{1}H NMR (400 MHz, CDCl$_3$) δ 11.13 (bs, 1H), 8.45 (d, *J* = 9.6 Hz, 1H), 8.32 – 3.22 (m, 3H), 7.92 (d, *J* = 3.9 Hz, 1H), 7.64 – 7.54 (m, 2H), 7.52 – 7.44 (m, 2H), 7.41 (s, 1H), 7.30 (d, *J* = 3.8 Hz, 1H), 7.21 – 7.08 (m, 2H), 2.60 – 2.30 (m, 4H), 1.99 – 1.77 (m, 2H); ^{13}C NMR (100 MHz, CDCl$_3$) δ 205.6, 197.2, 177.1, 141.2, 138.1, 137.2, 136.7, 136.4, 136.0, 134.6, 134.3, 129.7, 129.1, 124.6, 123.3, 123.1, 117.0, 113.7, 39.7, 36.8, 30.6, 20.4; HRMS (EI, [M+Na]$^+$) for $C_{24}H_{20}O_3Na^+$ calcd. 379.1305, found 379.1306.

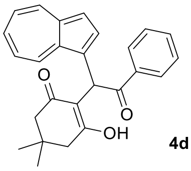

4d

2-(1-(Azulen-1-yl)-2-oxo-2-phenylethyl)-3-hydroxy-5,5-dimethylcyclohex-2-en-1-one (4d). Yield: 267 mg, 69% (Procedure A, 1 mmol scale); black-blue solid; mp: 150-152°C; ^{1}H NMR (400 MHz, CDCl$_3$) δ 10.80 (s, 1H), 8.40 (d, *J* = 9.7 Hz, 1H), 8.30 – 8.22 (m, 3H), 7.89 (d, *J* = 3.9 Hz, 1H), 7.64 – 7.52 (m, 2H), 7.52 – 7.44 (m, 2H), 7.35 (s, 1H), 7.30 (d, *J* = 3.8 Hz, 1H), 7.18 –7.08 (m, 2H), 2.43 (d, *J* = 17.7 Hz, 1H), 2.37 – 2.23 (m, 3H), 1.03 (s, 3H), 0.91 (s, 3H); ^{13}C NMR (100 MHz, CDCl$_3$) δ 205.3, 196.8, 175.3, 141.3, 138.1, 137.2, 136.7, 136.4, 136.0, 134.6, 134.3, 129.7, 129.1, 124.5, 123.3, 123.1, 117.0, 112.7, 50.6, 44.1, 39.8, 31.6, 29.4, 27.1; HRMS (EI, [M+Na]$^+$) for $C_{26}H_{24}O_3Na^+$ calcd. 407.1618, found 407.1618.

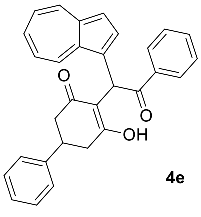

4e

4-(1-(Azulen-1-yl)-2-oxo-2-phenylethyl)-5-hydroxy-1,6-dihydro-[1,1'-biphenyl]-3(2*H*)-one (4e). Yield: 95 mg, 37% (Procedure A, 0.6 mmol scale, column chromatography was conducted using CH_2Cl_2/petroleum ether (1:1) as eluent, after which the product was washed with hexane); black-blue solid; mp: 70-72°C; in NMR, observed as as a mixture of two interconvertible diastereomeric enol forms

(dr = 2:1); ^{1}H NMR (400 MHz, $CDCl_3$) δ 11.53 (s, 0.67H), 10.92 (s, 0.33H), 8.54 (d, *J* = 9.6 Hz, 0.67H), 8.41 (d, *J* = 9.7 Hz, 0.33H), 8.33 – 8.20 (m, 3H), 7.95 (d, *J* = 3.3 Hz, 0.67H), 7.87 (d, *J* = 3.4 Hz, 0.33H), 7.66 – 6.94 (m, 13H), 3.34 – 3.29 (m, 0.33H), 3.27 – 3.09 (m, 0.67H), 2.86 – 2.50 (m, 4H); ^{13}C NMR (100 MHz, $CDCl_3$) δ 205.8, 205.0, 196.3, 196.1, 176.3, 176.8, 142.6, 141.30, 141.28, 138.2, 138.1, 137.29, 137.26, 136.7, 136.4, 136.0, 134.7, 134.6, 134.4, 134.3, 129.8, 129.7, 129.1, 128.9, 128.7, 127.1, 127.0, 126.8, 126.7, 124.4, 124.2, 123.41, 123.38, 123.3, 117.1, 113.7, 113.5, 43.8, 43.6, 39.9, 39.7, 38.34, 38.29, 38.25, 37.5; HRMS (EI, $[M+H]^+$) for $C_{30}H_{25}O_3^+$ calcd. 433.1798, found 433.1797.

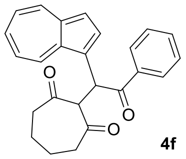

4f

2-(1-(Azulen-1-yl)-2-oxo-2-phenylethyl)cycloheptane-1,3-dione (4f). Yield: 135 mg, 91% (Procedure A, 0.4 mmol scale); purple solid; mp: 170-172°C; ^{1}H NMR (400 MHz, $CDCl_3$) δ 8.82 (d, *J* = 9.8 Hz, 1H), 8.18 (d, *J* = 9.4 Hz, 1H), 7.98 – 7.86 (m, 2H), 7.76 (d, *J* = 3.8 Hz, 1H), 7.65 – 7.54 (m, 1H), 7.38 – 7.29 (m, 2H), 7.28 – 7.19 (m, 3H), 7.16 – 7.07 (m, 1H), 6.24 (d, *J* = 10.5 Hz, 1H), 5.55 (d, *J* = 10.5 Hz, 1H), 3.02 – 2.88 (m, 1H), 2.57 – 2.45 (m, 1H), 2.37 – 2.11 (m, 4H), 1.88 – 1.67 (m, 2H); ^{13}C NMR (100 MHz, $CDCl_3$) δ 204.2, 203.0, 198.9, 141.9, 138.2, 137.2, 136.8, 136.7, 136.0, 134.7, 132.8, 128.7, 128.5, 123.7, 123.4, 123.0, 117.7, 71.0, 45.4, 44.6, 24.5, 24.1; HRMS (EI, $[M+Na]^+$) for $C_{25}H_{22}O_3Na^+$ calcd. 393.1461, found 393.1461.

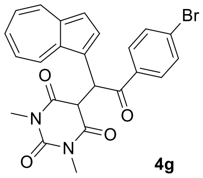

4g

5-(1-(Azulen-1-yl)-2-(4-bromophenyl)-2-oxoethyl)-1,3-dimethylpyrimidine-2,4,6(1*H*,3*H*,5*H*)-trione (4g). Yield: 388 mg, 81% (Procedure A, 1 mmol scale); black-blue solid; mp: 171-172°C; ^{1}H NMR (400 MHz, $CDCl_3$) δ 8.52 (d, *J* = 9.7 Hz, 1H), 8.34 (d, *J* = 9.4 Hz, 1H), 7.75 – 7.65 (m, 1H), 7.62 (d, *J* = 3.9 Hz, 1H), 7.53 (d, *J* = 8.5 Hz, 2H), 7.41 – 7.20 (m, 5H), 6.39 (d, *J* = 2.5 Hz, 1H), 3.84 (d, *J* = 2.5 Hz, 1H), 3.33 (s, 3H), 3.31 (s, 3H); ^{13}C NMR (100 MHz, $CDCl_3$) δ 197.5, 167.9, 167.4, 151.6, 141.5, 140.6, 138.0, 137.3, 135.0, 133.87, 131.85, 131.80, 130.8, 128.7, 124.3, 123.3, 123.2, 117.6, 51.0, 50.2, 29.0, 28.7; HRMS (EI, $[M+Na]^+$) for $C_{24}H_{19}BrN_2O_4Na^+$ calcd. 501.0420, found 501.0408.

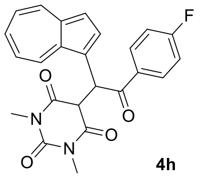

4h

5-(1-(Azulen-1-yl)-2-(4-fluorophenyl)-2-oxoethyl)-1,3-dimethylpyrimidine-2,4,6(1*H*,3*H*,5*H*)-trione (4h). Yield: 136 mg, 81% (Procedure A, 0.4 mmol scale); purple solid; mp: 151-152°C; ^{1}H NMR (400 MHz, $CDCl_3$) δ 8.52 (d, *J* = 9.7 Hz, 1H), 8.34 (d, *J* = 9.4 Hz, 1H), 7.76 – 7.66 (m, 3H), 7.64 (d, *J*

= 4.0 Hz, 1H), 7.37 – 7.21 (m, 3H), 6.95 – 6.83 (m, 2H), 6.41 (d, *J* = 2.5 Hz, 1H), 3.83 (d, *J* = 2.6 Hz, 1H), 3.34 (s, 3H), 3.31 (s, 3H); ^{13}C NMR (100 MHz, $CDCl_3$) δ 196.9, 168.0, 167.5, 165.7 (d, *J* = 256.0 Hz), 151.7, 141.4, 140.7, 138.0, 137.3, 134.9, 132.1 (d, *J* = 9.4 Hz), 131.8, 131.4 (d, *J* = 2.7 Hz), 124.2, 123.5, 123.2, 117.5, 115.7 (d, *J* = 21.9 Hz), 51.0, 50.3, 29.0, 28.7; HRMS (EI, $[M+H]^+$) for $C_{24}H_{20}FN_2O_4^+$ calcd. 419.1402, found 419.1402.

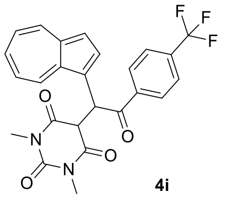

4i

5-(1-(Azulen-1-yl)-2-oxo-2-(4-(trifluoromethyl)phenyl)ethyl)-1,3-dimethylpyrimidine-2,4,6(1*H*,3*H*,5*H*)-trione (4i). Yield: 181 mg, 97% (Procedure A, 0.4 mmol scale); black-blue solid; mp: 147-148°C; ^{1}H NMR (400 MHz, $CDCl_3$) δ 8.53 (d, *J* = 9.7 Hz, 1H), 8.35 (d, *J* = 9.4 Hz, 1H), 7.78 (d, *J* = 8.1 Hz, 2H), 7.75 – 7.67 (m, 1H), 7.62 (d, *J* = 4.0 Hz, 1H), 7.49 (d, *J* = 8.3 Hz, 2H), 7.38 – 7.31 (m, 1H), 7.30 – 7.23 (m, 2H), 6.44 (d, *J* = 2.7 Hz, 1H), 3.90 (d, *J* = 2.8 Hz, 1H), 3.34 (s, 3H), 3.31 (s, 3H); ^{13}C NMR (100 MHz, $CDCl_3$) δ 197.6, 167.8, 167.4, 151.6, 141.4, 140.5, 138.1, 137.9, 137.4, 135.1, 134.5 (q, *J* = 32.7 Hz), 131.9, 129.6, 125.6 (q, *J* = 3.6 Hz), 124.4, 123.5 (q, *J* = 273.0 Hz), 123.3, 122.7, 117.6, 51.0, 50.3, 29.0, 28.7; HRMS (EI, $[M+Na]^+$) for $C_{25}H_{19}F_3N_2O_4Na^+$ calcd. 491.1189, found 491.1189.

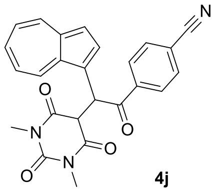

4j

4-(2-(Azulen-1-yl)-2-(1,3-dimethyl-2,4,6-trioxohexahydropyrimidin-5-yl)acetyl)benzonitrile (4j). Yield: 31 mg, 58% (Procedure A, 0.125 mmol scale); black-blue solid; mp: 101-103°C; ^{1}H NMR (400 MHz, $CDCl_3$) δ 8.51 (d, *J* = 9.7 Hz, 1H), 8.33 (d, *J* = 9.4 Hz, 1H), 7.77 – 7.66 (m, 3H), 7.59 (d, *J* = 3.9 Hz, 1H), 7.49 (d, *J* = 8.4 Hz, 2H), 7.38 – 7.22 (m, 3H), 6.40 (d, *J* = 2.5 Hz, 1H), 3.92 (d, *J* = 2.5 Hz, 1H), 3.32 (s, 3H), 3.29 (s, 3H); ^{13}C NMR (100 MHz, $CDCl_3$) δ 197.3, 167.7, 167.3, 151.5, 141.5, 140.4, 138.4, 138.2, 137.5, 135.2, 132.3, 131.9, 129.6, 124.5, 123.4, 122.3, 117.8, 117.6, 116.4, 51.0, 50.1, 29.0, 28.7; HRMS (EI, $[M+Na]^+$) for $C_{25}H_{19}N_3O_4Na^+$ calcd. 448.1268, found 448.1259.

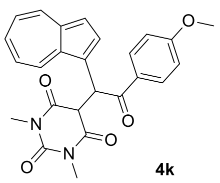

4k

5-(1-(Azulen-1-yl)-2-(4-methoxyphenyl)-2-oxoethyl)-1,3-dimethylpyrimidine-2,4,6(1*H*,3*H*,5*H*)-trione (4k). Yield: 66 mg, 77% (Procedure A, 0.2 mmol scale); dark blue solid; mp: 105-107°C; ^{1}H NMR (400 MHz, $CDCl_3$) δ 8.53 (d, *J* = 9.7 Hz, 1H), 8.32 (d, *J* = 9.3 Hz, 1H), 7.72 – 6.62 (m, 4H), 7.34

– 7.19 (m, 3H), 6.68 (d, *J* = 9.0 Hz, 2H), 6.42 (d, *J* = 2.7 Hz, 1H), 3.78 (d, *J* = 2.8 Hz, 1H), 3.73 (s, 3H), 3.34 (s, 3H), 3.32 (s, 3H); ^{13}C NMR (100 MHz, $CDCl_3$) δ 196.9, 168.2, 167.7, 163.7, 151.8, 141.4, 140.8, 137.8, 137.1, 134.7, 131.83, 131.76, 127.9, 124.4, 124.0, 123.0, 117.4, 113.7, 55.5, 51.0, 50.4, 29.0, 28.6; HRMS (EI, [M+H]$^+$) for $C_{25}H_{23}N_2O_5^+$ calcd. 431.1601, found 431.1596.

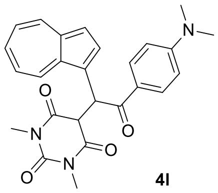

4l

5-(1-(Azulen-1-yl)-2-(4-(dimethylamino)phenyl)-2-oxoethyl)-1,3-dimethylpyrimidine-2,4,6(1*H*,3*H*,5*H*)-trione (4l). Yield: 105 mg, 59% (Procedure A, 0.4 mmol scale); grey blue solid; mp: 191-193°C; ^{1}H NMR (400 MHz, $CDCl_3$) δ 8.54 (d, *J* = 9.7 Hz, 1H), 8.31 (d, *J* = 9.3 Hz, 1H), 7.71 (d, *J* = 3.9 Hz, 1H), 7.69 – 7.61 (m, 1H), 7.57 (d, *J* = 9.1 Hz, 2H), 7.33 – 7.16 (m, 3H), 6.44 – 6.35 (m, 3H), 3.72 (d, *J* = 2.8 Hz, 1H), 3.34 (s, 3H), 3.32 (s, 3H), 2.94 (s, 6H); ^{13}C NMR (100 MHz, $CDCl_3$) δ 195.9, 168.4, 167.9, 153.5, 151.9, 141.5, 141.1, 137.6, 136.9, 134.6, 131.8, 131.7, 125.5, 123.8, 122.7, 122.6, 117.4, 110.5, 51.1, 50.3, 39.9, 28.9, 28.6; HRMS (EI, [M+Na]$^+$) for $C_{26}H_{25}N_3O_4Na^+$ calcd. 466.1737, found 466.1733.

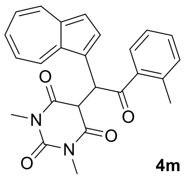

4m

5-(1-(Azulen-1-yl)-2-oxo-2-(o-tolyl)ethyl)-1,3-dimethylpyrimidine-2,4,6(1*H*,3*H*,5*H*)-trione (4m). Yield: 141 mg, 81% (Procedure A, 0.42 mmol scale); purple solid; mp: 132-134°C; ^{1}H NMR (400 MHz, $CDCl_3$) δ 8.37 (d, *J* = 9.2 Hz, 1H), 8.16 (d, *J* = 8.7 Hz, 1H), 7.64 – 7.44 (m, 2H), 7.33 – 6.99 (m, 6H), 6.87 – 6.71 (m, 1H), 6.31 (s, 1H), 3.85 (s, 1H), 3.22 (s, 3H), 3.17 (s, 3H), 2.45 (s, 3H); ^{13}C NMR (100 MHz, $CDCl_3$) δ 202.1, 168.0, 167.8, 151.6, 141.2, 140.2, 139.0, 137.8, 137.0, 136.0, 135.5, 132.0, 131.9, 131.6, 128.9, 125.5, 123.9, 123.4, 123.0, 117.4, 51.6, 51.1, 28.9, 28.7, 21.3; HRMS (EI, [M+Na]$^+$) for $C_{25}H_{22}N_2O_4Na^+$ calcd. 437.1472, found 437.1472.

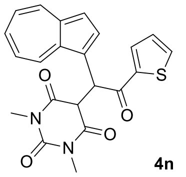

4n

5-(1-(Azulen-1-yl)-2-oxo-2-(thiophen-2-yl)ethyl)-1,3-dimethylpyrimidine-2,4,6(1*H*,3*H*,5*H*)-trione (4n). Yield: 274 mg, 84% (Procedure A, 0.8 mmol scale); black-blue solid; mp: 99-101°C; ^{1}H NMR (400 MHz, $CDCl_3$) δ 8.53 (d, *J* = 9.7 Hz, 1H), 8.36 (d, *J* = 9.3 Hz, 1H), 7.88 (d, *J* = 3.5 Hz, 1H), 7.74 – 7.63 (m, 1H), 7.47 (d, *J* = 4.5 Hz, 1H), 7.38 – 7.21 (m, 3H), 7.11 (d, *J* = 3.1 Hz, 1H), 6.81 (t, *J* = 4.1 Hz, 1H), 6.32 (d, *J* = 1.3 Hz, 1H), 3.82 (d, *J* = 1.4 Hz, 1H), 3.34 (s, 3H), 3.30 (s, 3H); ^{13}C NMR (100 MHz,

CDCl$_3$) δ 191.2, 167.9, 167.5, 151.6, 141.6, 141.5, 140.8, 138.0, 137.3, 135.6, 134.6, 134.4, 132.0, 128.1, 124.2, 123.8, 123.3, 117.5, 51.1, 51.0, 29.0, 28.7; HRMS (EI, $[M+Na]^+$) for $C_{22}H_{18}N_2O_4SNa^+$ calcd. 429.0829, found 429.0827.

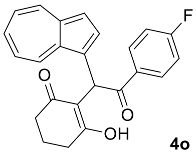

4o

2-(1-(Azulen-1-yl)-2-(4-fluorophenyl)-2-oxoethyl)-3-hydroxycyclohex-2-en-1-one (4o). Yield: 140 mg, 62% (Procedure A, 0.6 mmol scale); dark blue solid ; mp: 182-184°C; ^{1}H NMR (400 MHz, CDCl$_3$) δ 10.93 (s, 1H), 8.39 (d, *J* = 9.7 Hz, 1H), 8.34 – 8.25 (m, 3H), 7.89 (d, *J* = 3.9 Hz, 1H), 7.63 – 7.54 (m, 1H), 7.34 (m, 1H), 7.30 (d, *J* = 3.9 Hz, 1H), 7.21 – 7.10 (m, 4H), 2.59 – 2.31 (m, 4H), 1.99 – 1.78 (m, 2H); ^{13}C NMR (100 MHz, CDCl$_3$) δ 203.8, 197.2, 177.2, 166.8 (d, *J* = 257.4 Hz), 141.3, 138.2, 137.3, 136.7, 136.4, 134.2, 132.6 (d, *J* = 9.7 Hz), 132.4 (d, *J* = 2.9 Hz), 124.3, 123.4, 123.2, 117.0, 116.3 (d, *J* = 22.0 Hz), 113.5, 39.6, 36.8, 30.6, 20.4; HRMS (EI, $[M+Na]^+$) for $C_{24}H_{19}FO_3Na^+$ calcd. 397.1210, found 397.1210.

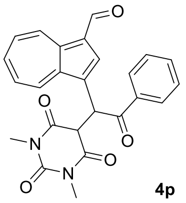

4p

3-(1-(1,3-Dimethyl-2,4,6-trioxohexahydropyrimidin-5-yl)-2-oxo-2-phenylethyl)azulene-1-carbaldehyde (4p). Yield: 240 mg, 56% (Procedure A, 1 mmol scale); dark red solid; mp: 178-180°C; ^{1}H NMR (400 MHz, CDCl$_3$) δ 10.18 (s, 1H), 9.66 (d, *J* = 9.6 Hz, 1H), 8.73 (d, *J* = 9.9 Hz, 1H), 8.04 (s, 1H), 8.01 – 7.89 (m, 1H), 7.77 – 7.62 (m, 4H), 7.50 – 7.39 (m, 1H), 7.32 – 7.20 (m, 2H), 6.40 (d, *J* = 2.4 Hz, 1H), 3.80 (d, *J* = 2.4 Hz, 1H), 3.37 (s, 3H), 3.34 (s, 3H); ^{13}C NMR (100 MHz, CDCl$_3$) δ 197.9, 187.1, 167.9, 167.4, 151.5, 146.3, 141.0, 140.7, 140.3, 138.6, 134.7, 134.2, 133.9, 130.8, 129.3, 128.7, 128.6, 124.9, 124.8, 50.3, 49.6, 29.1, 28.7; HRMS (EI, $[M+H]^+$) for $C_{25}H_{21}N_2O_5^+$ calcd. 429.1445, found 429.1445.

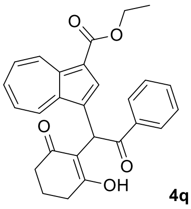

4q

Ethyl 3-(1-(2-hydroxy-6-oxocyclohex-1-en-1-yl)-2-oxo-2-phenylethyl)azulene-1-carboxylate (4q). Yield: 247 mg, 96% (Procedure B, 0.6 mmol scale); dark purple solid; mp: 88-100°C; ^{1}H NMR (400 MHz, CDCl$_3$) δ 11.07 (s, 1H), 9.63 (d, *J* = 10.0 Hz, 1H), 8.55 (d, *J* = 9.7 Hz, 1H), 8.33 (s, 1H), 8.31 – 8.24 (m, 2H), 7.83 – 7.74 (m, 1H), 7.67 – 7.59 (m, 1H), 7.55 – 7.47 (m, 3H), 7.47 – 7.39 (m, 1H), 7.34 (s, 1H), 4.40 (q, *J* = 7.1 Hz, 2H), 2.59 – 2.32 (m, 4H), 1.99 – 1.76 (m, 2H), 1.43 (t, *J* = 7.1 Hz, 3H); ^{13}C

NMR (100 MHz, $CDCl_3$) δ 205.2, 197.1, 177.6, 165.3, 141.7, 141.1, 139.7, 139.4, 138.1, 135.8, 135.7, 134.9, 129.8, 129.2, 128.1, 127.0, 124.0, 115.7, 113.2, 60.0, 39.2, 36.8, 30.6, 20.4, 14.8; HRMS (EI, $[M+Na]^+$) for $C_{27}H_{24}O_5Na^+$ calcd. 451.1516, found 451.1510.

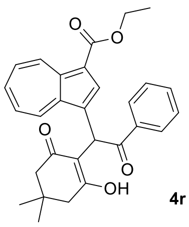

4r

Ethyl 3-(1-(2-hydroxy-4,4-dimethyl-6-oxocyclohex-1-en-1-yl)-2-oxo-2-phenylethyl)azulene-1-carboxylate (4r). Yield: 165 mg, 90% (Procedure B, 0.4 mmol scale); dark purple solid ; mp: 88-89°C; ^{1}H NMR (400 MHz, $CDCl_3$) δ 10.80 (bs, 1H), 9.63 (d, J = 9.9 Hz, 1H), 8.52 (d, J = 9.7 Hz, 1H), 8.32 (s, 1H), 8.30 – 8.24 (m, 2H), 7.83 – 7.74 (m, 1H), 7.67 – 7.59 (m, 1H), 7.55 – 7.47 (m, 3H), 7.45 – 7.37 (m, 1H), 7.28 (s, 1H), 4.44 – 4.35 (m, 2H), 2.43 (d, J = 17.8 Hz, 1H), 2.38 – 2.24 (m, 4H), 1.43 (t, J = 7.1 Hz, 3H), 1.02 (s, 3H), 0.89 (s, 3H); ^{13}C NMR (100 MHz, $CDCl_3$) δ 205.0, 196.8, 175.9, 165.3, 141.8, 141.1, 139.7, 139.4, 138.1, 135.80, 135.77, 134.9, 129.7, 129.2, 128.1, 126.9, 124.0, 115.7, 112.3, 60.0, 50.5, 44.1, 39.2, 31.6, 29.5, 26.9, 14.8; HRMS (EI, $[M+Na]^+$) for $C_{27}H_{28}O_5Na^+$ calcd. 479.1829, found 479.1821.

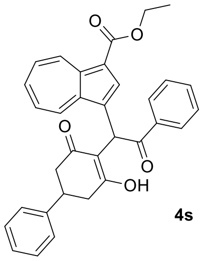

4s

Ethyl 3-(1-(5-hydroxy-3-oxo-1,2,3,6-tetrahydro-[1,1'-biphenyl]-4-yl)-2-oxo-2-phenylethyl)azulene-1-carboxylate (4s). Yield: 33 mg, 44% (Procedure B, 0.15 mmol scale, the product was washed with pentane after column chromatography); dark purple solid; mp: 87-89°C; in NMR, observed as as a mixture of two interconvertible diastereomeric enol forms (dr = 2:1); ^{1}H NMR (400 MHz, $CDCl_3$) δ 11.44 (bs, 0.67H), 10.93 (bs, 0.33H), 9.75 – 9.55 (m, 1H), 8.66 (d, J = 9.7 Hz, 0.67H), 8.49 (d, J = 9.6 Hz, 0.33H), 8.39 (s, 0.67H), 8.34 (s, 0.33H), 8.32 – 8.20 (m, 2H), 7.86 – 7.71 (m, 1H), 7.68 – 7.01 (m, 11H), 4.49 – 4.33 (m, 2H), 3.44 – 3.30 (m, 0.33H), 3.27 – 3.10 (s, 0.67H), 2.90 – 2.52 (m, 4H), 1.49 – 1.37 (m, 3H); ^{13}C NMR (100 MHz, $CDCl_3$) δ 205.4, 204.7, 196.3, 196.0, 176.8, 176.2, 165.3, 142.5, 142.4, 141.7, 141.2, 141.1, 139.8, 139.6, 139.4, 138.2, 138.1, 135.79, 135.76, 134.93, 134.85, 129.8, 129.7, 129.2, 128.9, 128.7, 128.2, 128.1, 127.2, 127.10, 127.07, 127.0, 126.74, 126.70, 123.9, 123.7, 115.9, 115.8, 113.2, 113.1, 60.03, 59.99, 43.8, 43.6, 39.4, 39.2, 38.3, 38.1, 37.3, 14.8; HRMS (EI, $[M+Na]^+$) for $C_{33}H_{28}O_5Na^+$ calcd. 527.1829, found 527.1816.

General procedure for the synthesis of azulene-tetrahydrocinnolin-5-one conjugates 5a,5b from azulene-containing MCR adducts 4c,4d

Azulene derivative **4c,d** (0.4 mmol, 1 equiv) was placed in a screw-capped vial and dissolved in methanol (2 mL) followed by addition of hydrazine monohydrate (20 mg, 0.4 mmol, 1 equiv). The reaction mixture was stirred for 24h at room temperature. The reaction progress was monitored by TLC. After completion of reaction, the mixture was diluted with CH_2Cl_2 and evaporated with silica gel under reduced pressure. Column chromatography with CH_2Cl_2/EtOAc (19:1) as eluent delivered azulene-tetrahydrocinnolin-5-one conjugate **5a**. For the isolation of product **5b**, pure CH_2Cl_2 was used as an eluent.

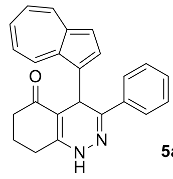

5a

4-(Azulen-1-yl)-3-phenyl-4,6,7,8-tetrahydrocinnolin-5(1*H*)-one (5a). Yield: 132 mg, 94%; blue solid; mp: 236-238°C; ^{1}H NMR (400 MHz, [D$_6$]DMSO) δ 10.96 (s, 1H), 9.02 (d, *J* = 9.8 Hz, 1H), 8.28 (d, *J* = 9.3 Hz, 1H), 7.75 – 7.62 (m, 3H), 7.49 (d, *J* = 3.8 Hz, 1H), 7.35 – 7.19 (m, 5H), 7.19 – 7.11 (m, 1H), 5.77 (s, 1H), 2.56 – 2.48 (m, 2H, overlaps with the signal of [D$_6$]DMSO), 2.31 – 2.09 (m, 2H), 1.94 – 1.82 (m, 1H), 1.70 – 1.55 (m, 1H); ^{13}C NMR (100 MHz, [D$_6$]DMSO) δ 193.9, 150.1, 147.0, 140.5, 138.1, 136.9, 136.2, 136.1, 134.8, 133.2, 132.0, 128.9, 128.4, 125.8, 122.7, 122.6, 117.3, 104.6, 36.7, 27.7, 24.5, 20.6; HRMS (EI, [M+Na]$^+$) for $C_{24}H_{20}N_2ONa^+$ calcd. 375.1468, found 375.1464.

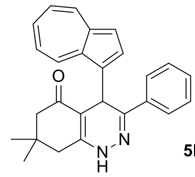

5b

4-(Azulen-1-yl)-8,8-dimethyl-3-phenyl-4,6,7,8-tetrahydrocinnolin-5(1*H*)-one (5b). Yield: 141 mg, 93%; blue solid; mp: 199-202°C; ^{1}H NMR (400 MHz, [D$_6$]DMSO) δ 10.92 (s, 1H), 8.96 (d, *J* = 9.8 Hz, 1H), 8.27 (d, *J* = 9.3 Hz, 1H), 7.78 – 7.70 (m, 2H), 7.70 – 7.62 (m, 1H), 7.54 (d, *J* = 3.8 Hz, 1H), 7.35 – 7.19 (m, 5H), 7.19 – 7.10 (m, 1H), 5.74 (s, 1H), 2.45 (d, *J* = 16.7 Hz, 1H), 2.30 (d, *J* = 16.5 Hz, 1H), 2.20 (d, *J* = 16.1 Hz, 1H), 1.94 (d, *J* = 16.4 Hz, 1H), 1.00 (s, 3H), 0.67 (s, 3H); ^{13}C NMR (100 MHz, [D$_6$]DMSO) δ 193.4, 148.4, 147.0, 140.5, 138.0, 136.9, 136.2, 136.1, 134.8, 133.1, 131.9, 128.9, 128.3, 125.8, 122.7, 122.6, 117.3, 103.4, 50.3, 37.7, 32.2, 29.0, 27.7, 26.1; HRMS (EI, [M+Na]$^+$) for $C_{26}H_{24}N_2ONa^+$ calcd. 403.1781, found 403.1774.

Synthesis of azulene-dihydroindol-4-one conjugates 7 from azulene-containing MCR adducts 4

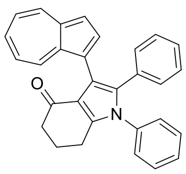

7a

3-(Azulen-1-yl)-1,2-diphenyl-1,5,6,7-tetrahydro-4*H*-indol-4-one (7a). Azulene-containing adduct **4c** (36 mg, 0.1 mmol, 1 equiv) was placed in a screw-capped vial and dissolved in *i*PrOH (1 mL) followed by addition of aniline (14 mg, 0.15 mmol, 1.5 equiv). The mixture was sealed, placed in an oil bath preheated at 80 °C and stirred for 6 h. The resulting mixture was diluted with EtOAc and evaporated with silica gel under reduced pressure. Column chromatography with petroleum ether/EtOAc (7:1) as eluent delivered azulene-dihydroindol-4-one conjugate **7a**. Yield: 37 mg, 89%; dark blue solid; mp: 191-192°C; ^{1}H NMR (400 MHz, $CDCl_3$) δ 8.21 (d, *J* = 9.3 Hz, 1H), 8.03 (d, *J* = 9.8 Hz, 1H), 7.84 (d, *J* = 3.8 Hz, 1H), 7.45 – 7.31 (m, 5H), 7.25 – 7.14 (m, 2H), 7.07 – 6.98 (m, 1H), 6.96 – 6.82 (m, 4H), 6.79 – 6.72 (m, 2H), 2.88 – 2.69 (m, 2H), 2.60 – 2.47 (m, 2H), 2.26 – 2.14 (m, 2H); ^{13}C NMR (100 MHz, $CDCl_3$) δ 193.7, 145.0, 141.5, 140.0, 137.9, 137.1, 136.13, 136.11, 136.07, 133.8, 131.8, 130.4, 129.2, 128.2, 128.1, 127.8, 126.6, 123.2, 122.5, 122.0, 119.7, 117.0, 116.8, 39.2, 23.8, 23.6; HRMS (EI, $[M+H]^+$) for $C_{30}H_{24}NO^+$ calcd. 414.1852, found 414.1843.

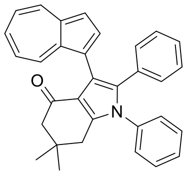

7b

3-(Azulen-1-yl)-6,6-dimethyl-1,2-diphenyl-1,5,6,7-tetrahydro-4*H*-indol-4-one (7b). Azulene-containing adduct **4d** (38 mg, 0.1 mmol, 1 equiv) was placed in a screw-capped vial and dissolved in *i*PrOH (1 mL) followed by addition of aniline (10 mg, 0.11 mmol, 1.1 equiv). The mixture was sealed, placed in an oil bath preheated at 80 °C and stirred for 8 h. The resulting mixture was diluted with EtOAc and evaporated with silica gel under reduced pressure. Column chromatography with petroleum ether/EtOAc (17:3) as eluent delivered azulene-dihydroindol-4-one conjugate **7b**. Yield: 21 mg, 48%; dark blue solid; mp: 238-240°C; ^{1}H NMR (400 MHz, $CDCl_3$) δ 8.22 (d, *J* = 9.3 Hz, 1H), 8.03 (d, *J* = 9.7 Hz, 1H), 7.87 (d, *J* = 3.8 Hz, 1H), 7.45 – 7.33 (m, 5H), 7.26 – 7.13 (m, 2H), 7.07 – 6.99 (m, 1H), 6.97 – 6.83 (m, 4H), 6.80 – 6.74 (m, 2H), 2.66 (bs, 2H), 2.44 (s, 2H), 1.20 (s, 6H); ^{13}C NMR (100 MHz, $CDCl_3$) δ 193.2, 143.8, 141.5, 140.1, 137.8, 137.1, 136.1, 136.0, 133.9, 131.8, 130.3, 129.2, 128.17, 128.15, 127.7, 126.5, 123.1, 122.5, 122.0, 118.5, 117.0, 116.6, 53.2, 37.6, 35.1, 28.8; HRMS (EI, $[M+Na]^+$) for $C_{32}H_{27}NONa^+$ calcd. 464.1985, found 464.1985.

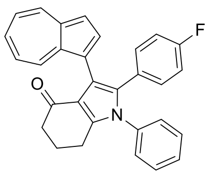

7c

3-(Azulen-1-yl)-2-(4-fluorophenyl)-1-phenyl-1,5,6,7-tetrahydro-4*H*-indol-4-one (7c). Azulene-containing adduct **4o** (150 mg, 0.4 mmol, 1 equiv) was placed in a screw-capped vial and dissolved in *i*PrOH (2 mL) followed by addition of aniline (56 mg, 0.6 mmol, 1.5 equiv). The mixture was sealed, placed in an oil bath preheated at 80 °C and stirred for 8 h. The resulting mixture was diluted with EtOAc and evaporated with silica gel under reduced pressure. Column chromatography with petroleum ether/EtOAc (9:1→17:3) as eluent followed by washing with pentane delivered azulene-dihydroindol-4-one conjugate **7c**. Yield: 108 mg, 63%; dark blue solid; mp: 180-182°C; ^{1}H NMR (400 MHz, $CDCl_3$) δ 8.22 (d, *J* = 9.3 Hz, 1H), 7.99 (d, *J* = 9.7 Hz, 1H), 7.82 (d, *J* = 3.8 Hz, 1H), 7.46 – 7.32 (m, 5H), 7.26 – 7.10 (m, 2H), 7.08 – 7.00 (m, 1H), 6.92 – 6.83 (m, 1H), 6.75 – 6.67 (m, 2H), 6.63 – 6.54 (m, 2H), 2.87 – 2.66 (m, 2H), 2.58 – 2.48 (m, 2H), 2.25 – 2.13 (m, 2H); ^{13}C NMR (100 MHz, $CDCl_3$) δ 193.8, 161.5 (d, *J* = 246.9 Hz), 145.0, 141.6, 140.0, 137.7, 137.2, 136.2, 136.1, 136.0, 132.7, 132.0 (d, *J* = 8.0 Hz), 129.3, 128.3, 128.1, 127.9 (d, *J* = 3.2 Hz), 123.0, 122.7, 122.1, 119.7, 117.1, 117.0, 114.9 (d, *J* = 21.5 Hz), 39.2, 23.8, 23.6; HRMS (EI, $[M+H]^+$) for $C_{30}H_{23}FNO^+$ calcd. 432.1758, found 432.1754.

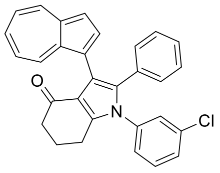

7d

3-(Azulen-1-yl)-1-(3-chlorophenyl)-2-phenyl-1,5,6,7-tetrahydro-4*H*-indol-4-one (7d). Azulene-containing adduct **4c** (71 mg, 0.2 mmol, 1 equiv) was placed in a screw-capped vial and dissolved in *i*PrOH (1 mL) followed by addition of 3-chloroaniline (28 mg, 0.22 mmol, 1.1 equiv). The mixture was sealed, placed in an oil bath preheated at 80 °C and stirred for 24 h. For the next purification steps, three batches of 0.2 mmol scale were combined together. The resulting mixture was diluted with EtOAc and evaporated with silica gel under reduced pressure. Column chromatography with petroleum ether/EtOAc (9:1→17:3) as eluent followed by washing with pentane delivered azulene-dihydroindol-4-one conjugate **7d**. Yield: 94 mg, 35% (combined yield for three reactions on 0.2 mmol scale); dark blue solid; mp: 188-190°C; ^{1}H NMR (400 MHz, $CDCl_3$) δ 8.21 (d, *J* = 9.3 Hz, 1H), 7.99 (d, *J* = 9.7 Hz, 1H), 7.81 (d, *J* = 3.1 Hz, 1H), 7.45 – 7.37 (m, 1H), 7.36 – 7.31 (m, 2H), 7.31 – 7.25 (m, 2H), 7.08 – 6.99 (m, 2H), 6.99 – 6.82 (m, 4H), 6.78 – 6.72 (m, 2H), 2.87 – 2.71 (m, 2H), 2.58 – 2.49 (m, 2H), 2.26 – 2.16 (m, 2H); ^{13}C NMR (100 MHz, $CDCl_3$) δ 193.6, 144.8, 141.5, 140.0, 139.0, 137.1, 136.18, 136.15, 136.0, 134.7, 133.7, 131.3, 130.3, 130.2, 128.5, 128.2, 127.9, 126.9, 126.5, 122.9, 122.6, 122.1, 119.9, 117.2, 117.1, 39.1, 23.8, 23.6; HRMS (EI, $[M+Na]^+$) for $C_{30}H_{22}ClNONa^+$ calcd. 470.1282, found 470.1282.

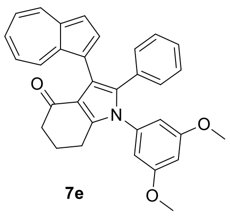

7e

3-(Azulen-1-yl)-1-(3,5-dimethoxyphenyl)-2-phenyl-1,5,6,7-tetrahydro-4*H*-indol-4-one (7e). Azulene-containing adduct **4c** (143 mg, 0.4 mmol, 1 equiv) was placed in a screw-capped vial and dissolved in *i*PrOH (2 mL) followed by addition of 3,5-dimethoxyaniline (92 mg, 0.6 mmol, 1.5 equiv). The mixture was sealed, placed in an oil bath preheated at 80 °C and stirred for 8 h. The resulting mixture was diluted with EtOAc and evaporated with silica gel under reduced pressure. Column chromatography with petroleum ether/EtOAc (9:1→4:1) as eluent delivered azulene-dihydroindol-4-one conjugate **7e**. Yield: 107 mg, 56%; dark blue solid; mp: 250-252°C; ^{1}H NMR (400 MHz, $CDCl_3$) δ 8.20 (d, *J* = 9.3 Hz, 1H), 8.01 (d, *J* = 9.7 Hz, 1H), 7.83 (d, *J* = 3.8 Hz, 1H), 7.44 – 7.36 (m, 1H), 7.33 (d, *J* = 3.8 Hz, 1H), 7.06 – 6.98 (m, 1H), 6.98 – 6.89 (m, 3H), 6.89 – 6.78 (m, 3H), 6.42 (t, *J* = 2.2 Hz, 1H), 6.32 (bs, 2H), 3.66 (s, 6H), 2.91 – 2.76 (m, 2H), 2.58 – 2.47 (m, 2H), 2.26 – 2.14 (m, 2H); ^{13}C NMR (100 MHz, $CDCl_3$) δ 193.7, 160.9, 144.9, 141.6, 140.1, 139.4, 137.1, 136.14, 136.11, 136.09, 133.7, 132.0, 130.2, 127.8, 126.7, 123.2, 122.5, 122.0, 119.7, 117.0, 116.8, 106.6, 100.3, 55.6, 39.2, 23.9, 23.7; HRMS (EI, $[M+H]^+$) for $C_{32}H_{27}NO_3^+$ calcd. 474.2064, found 474.2064.

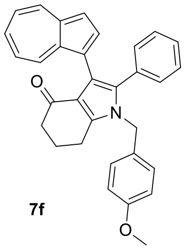

7f

3-(Azulen-1-yl)-1-(4-methoxybenzyl)-2-phenyl-1,5,6,7-tetrahydro-4*H*-indol-4-one (7f). Azulene-containing adduct **4c** (71 mg, 0.2 mmol, 1 equiv) was placed in a screw-capped vial and dissolved in *i*PrOH (1 mL) followed by addition of 4-methoxybenzylamine (41 mg, 0.3 mmol, 1.5 equiv). The mixture was sealed, placed in an oil bath preheated at 80 °C and stirred for 8 h. The resulting mixture was diluted with EtOAc and evaporated with silica gel under reduced pressure. Column chromatography with petroleum ether/EtOAc (9:1→17:3) as eluent delivered azulene-dihydroindol-4-one conjugate **7f**. Yield: 78 mg, 85%; blue solid; mp: 159-161°C; ^{1}H NMR (400 MHz, $CDCl_3$) δ 8.17 (d, *J* = 9.3 Hz, 1H), 7.99 (d, *J* = 9.7 Hz, 1H), 7.76 (d, *J* = 3.8 Hz, 1H), 7.44 – 7.35 (m, 1H), 7.28 (d, *J* = 3.9 Hz, 1H), 7.13 – 7.03 (m, 3H), 7.03 – 6.96 (m, 3H), 6.94 (d, *J* = 8.7 Hz, 2H), 6.91 – 6.83 (m, 3H), 5.09 (s, 2H), 3.82 (s, 3H), 2.83 – 2.71 (m, 2H), 2.56 – 2.44 (m, 2H), 2.24 – 2.13 (m, 2H); ^{13}C NMR (100 MHz, $CDCl_3$) δ 193.5, 159.1, 144.2, 141.4, 139.9, 137.0, 136.12, 136.05, 136.03, 134.1, 132.0, 130.7, 129.5, 128.3, 127.6, 127.2, 123.5, 122.4, 121.9, 119.5, 116.9, 116.5, 114.5, 55.4, 47.7, 39.1, 23.6, 23.0; HRMS (EI, $[M+H]^+$) for $C_{32}H_{28}NO_2^+$ calcd. 458.2115, found 458.2115.

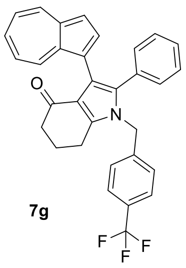

7g

3-(Azulen-1-yl)-2-phenyl-1-(4-(trifluoromethyl)benzyl)-1,5,6,7-tetrahydro-4*H*-indol-4-one (7g). Azulene-containing adduct **4c** (71 mg, 0.2 mmol, 1 equiv) was placed in a screw-capped vial and dissolved in *i*PrOH (1 mL) followed by addition of (4-(trifluoromethyl)phenyl)methanamine (39 mg, 0.22 mmol, 1.1 equiv). The mixture was sealed, placed in an oil bath preheated at 80 °C and stirred for 5 h. The resulting mixture was diluted with EtOAc and evaporated with silica gel under reduced pressure. Column chromatography with petroleum ether/EtOAc (4:1→7:3) as eluent delivered azulene-dihydroindol-4-one conjugate **7g**. Yield: 92 mg, 93%; dark blue solid; mp: 148-150°C; ^{1}H NMR (400 MHz, $CDCl_3$) δ 8.18 (d, *J* = 9.3 Hz, 1H), 8.00 (d, *J* = 9.7 Hz, 1H), 7.77 (d, *J* = 3.9 Hz, 1H), 7.63 (d, *J* = 8.1 Hz, 2H), 7.46 – 7.36 (m, 1H), 7.29 (d, *J* = 3.9 Hz, 1H), 7.17 – 6.94 (m, 8H), 6.93 – 6.85 (m, 1H), 5.19 (s, 2H), 2.80 – 2.68 (m, 2H), 2.57 – 2.45 (m, 2H), 2.26 – 2.14 (m, 2H); ^{13}C NMR (100 MHz, $CDCl_3$) δ 193.4, 143.9, 141.65, 141.64, 141.5, 139.9, 137.1, 136.2, 136.1, 135.9, 134.1, 131.6, 130.6, 130.1 (d, *J* = 32.6 Hz), 128.5, 127.8, 126.24, 126.15 (q, *J* = 3.7 Hz), 124.1 (q, *J* = 272.0 Hz), 123.1, 122.6, 122.0, 119.8, 117.0, 116.9, 47.8, 39.0, 23.5, 22.9; HRMS (EI, $[M+H]^+$) for $C_{32}H_{25}F_3NO^+$ calcd. 496.1883, found 496.1883.

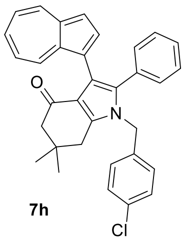

7h

3-(Azulen-1-yl)-1-(4-chlorobenzyl)-6,6-dimethyl-2-phenyl-1,5,6,7-tetrahydro-4*H*-indol-4-one (7h). Azulene-containing adduct **4d** (77 mg, 0.2 mmol, 1 equiv) was placed in a screw-capped vial and dissolved in *i*PrOH (1 mL) followed by addition of (4-chlorophenyl)methanamine (31 mg, 0.22 mmol, 1.1 equiv). The mixture was sealed, placed in an oil bath preheated at 80 °C and stirred for 4.5 h. The resulting mixture was diluted with EtOAc and evaporated with silica gel under reduced pressure. Column chromatography with petroleum ether/EtOAc (17:3) as eluent followed by washing with pentane delivered azulene-dihydroindol-4-one conjugate **7h**. Yield: 71 mg, 72%; blue solid; mp: 190-192°C; ^{1}H NMR (400 MHz, $CDCl_3$) δ 8.17 (d, *J* = 9.3 Hz, 1H), 7.96 (d, *J* = 9.7 Hz, 1H), 7.76 (d, *J* = 3.9 Hz, 1H), 7.44 – 7.36 (m, 1H), 7.34 (d, *J* = 8.4 Hz, 2H), 7.28 (d, *J* = 3.9 Hz, 1H), 7.13 – 6.90 (m, 8H), 6.90 – 6.82 (m, 1H), 5.10 (s, 2H), 2.61 (s, 2H), 2.40 (s, 2H), 1.15 (s, 6H); ^{13}C NMR (100 MHz, $CDCl_3$) δ 192.9, 142.9, 141.5, 139.9, 137.1, 136.2, 136.1, 136.0, 134.2, 133.5, 131.8, 130.6, 129.3, 128.4, 127.7, 127.2, 123.1, 122.5, 122.0, 118.5, 117.0, 116.6, 53.2, 47.5, 36.8, 35.3, 28.8; HRMS (EI, $[M+H]^+$) for $C_{33}H_{29}ClNO^+$ calcd. 490.1932, found 490.1932.

One-pot synthesis of azulene-dihydroindol-4-one conjugate 7a

Phenylglyoxal monohydrate (**2a**, 46 mg, 0.3 mmol, 1.5 equiv) was placed in a screw-capped vial and dissolved in *i*PrOH (1 mL) followed by addition of azulene (**1a**, 26 mg, 0.2 mmol, 1 equiv) and cyclohexane-1,3-dione (**3c**, 45 mg, 0.4 mmol, 2 equiv). The resulting mixture was sealed, placed in an oil bath preheated at 80 °C and stirred for 1h. Upon completion of this time, the aniline (**6a**, 41 mg, 0.44 mmol, 2.2 equiv) was added and the reaction was continued for another 8h. The resulting mixture was diluted with EtOAc and evaporated with silica gel under reduced pressure. Column chromatography with petroleum ether/EtOAc (17:3) as eluent delivered azulene-dihydroindol-4-one conjugate **7a**.

UV/vis absorption spectra

The UV/Vis absorption was measured in dichloromethane and in methanol at c ≅ $5 \cdot 10^{-6}$ M.

Compound	Spectra	Magnified spectra in a visible region
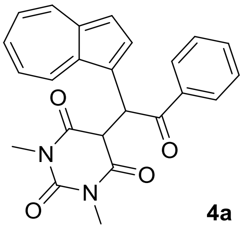 4a	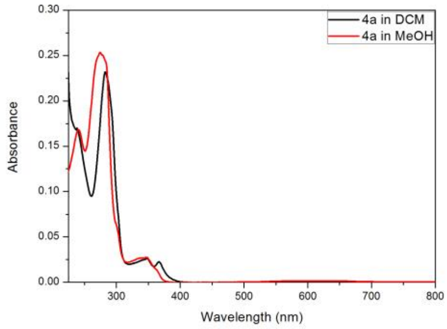	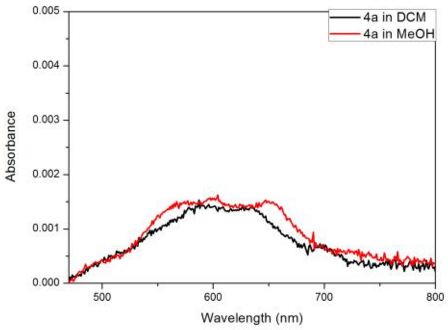
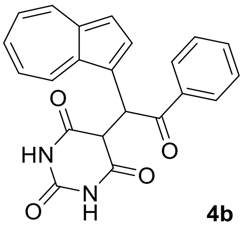 4b	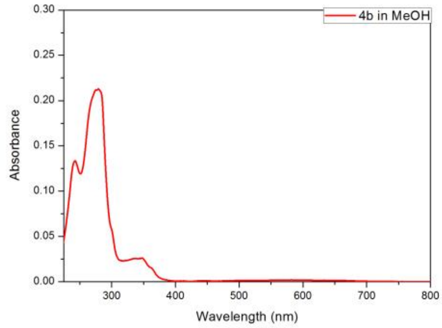	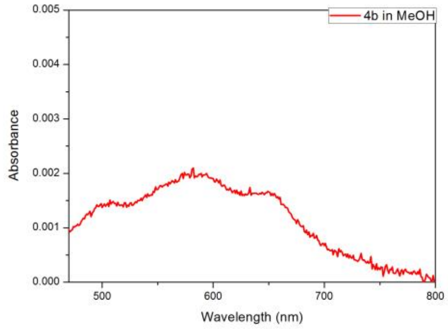
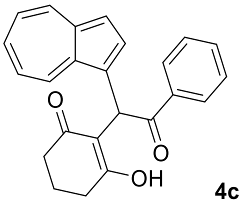 4c	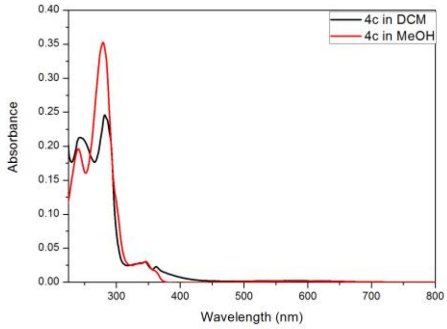	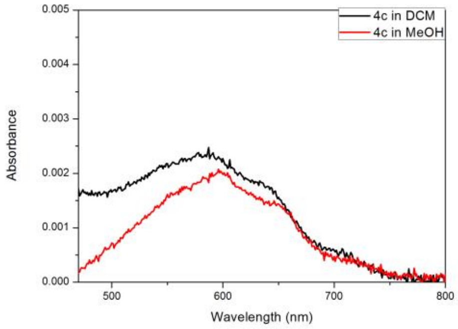

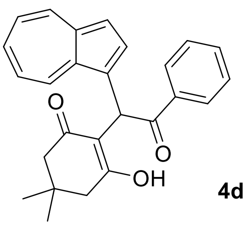 **4d**	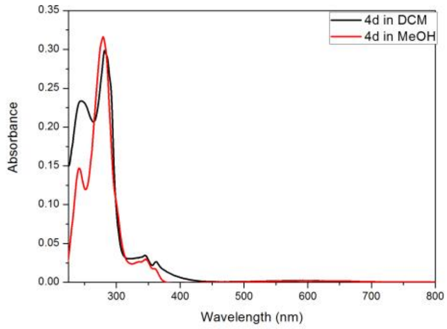4d in DCM<br>4d in MeOH<br>Absorbance<br>Wavelength (nm)	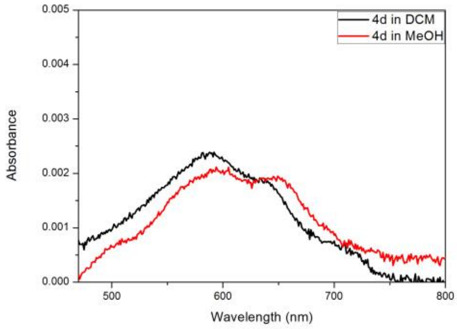4d in DCM<br>4d in MeOH<br>Absorbance<br>Wavelength (nm)
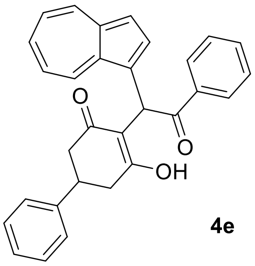 **4e**	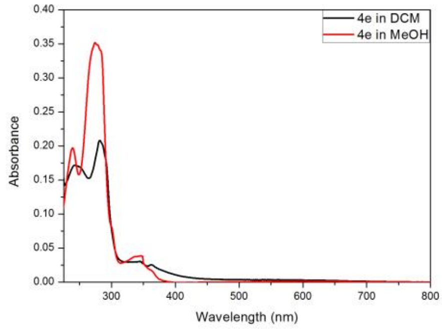4e in DCM<br>4e in MeOH<br>Absorbance<br>Wavelength (nm)	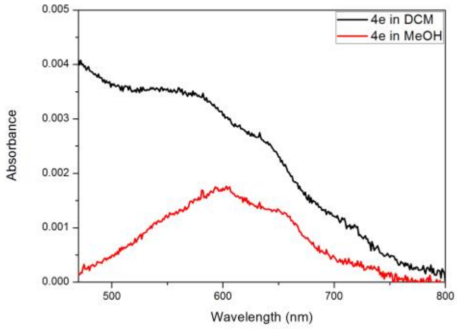4e in DCM<br>4e in MeOH<br>Absorbance<br>Wavelength (nm)
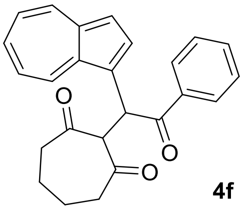 **4f**	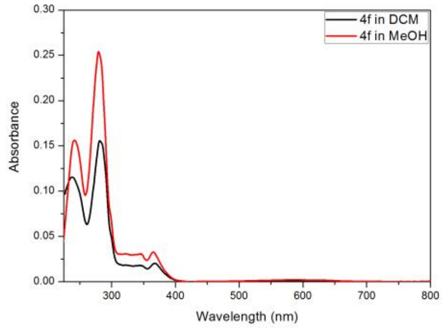4f in DCM<br>4f in MeOH<br>Absorbance<br>Wavelength (nm)	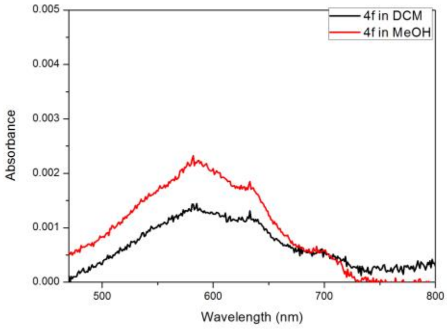4f in DCM<br>4f in MeOH<br>Absorbance<br>Wavelength (nm)
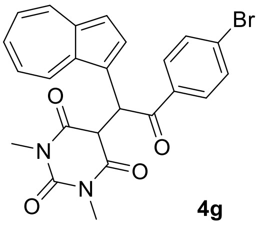 **4g**	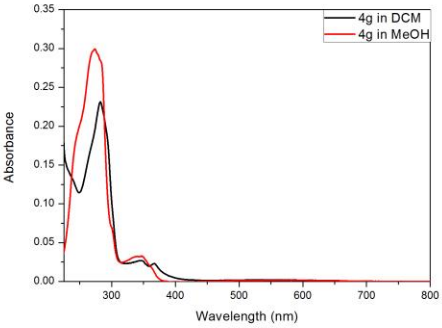4g in DCM<br>4g in MeOH<br>Absorbance<br>Wavelength (nm)	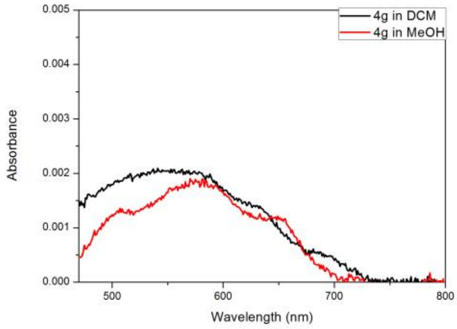4g in DCM<br>4g in MeOH<br>Absorbance<br>Wavelength (nm)

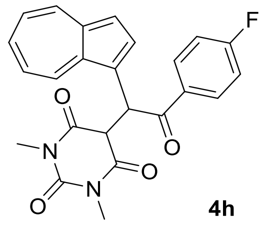

4h

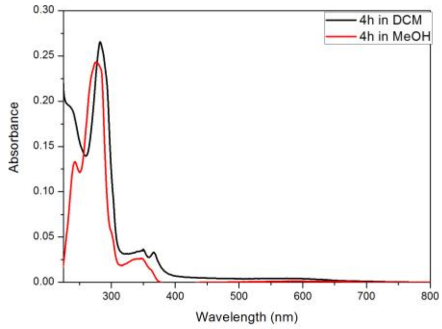
4h in DCM
4h in MeOH
Absorbance
Wavelength (nm)

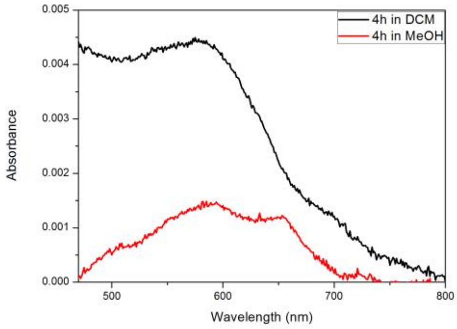
4h in DCM
4h in MeOH
Absorbance
Wavelength (nm)

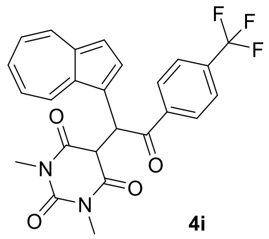

4i

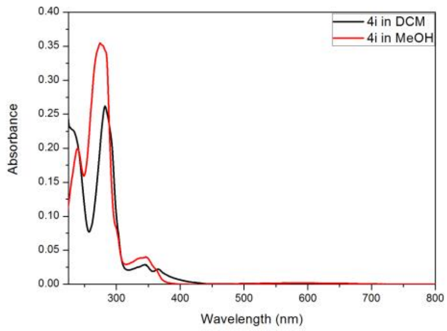
4i in DCM
4i in MeOH
Absorbance
Wavelength (nm)

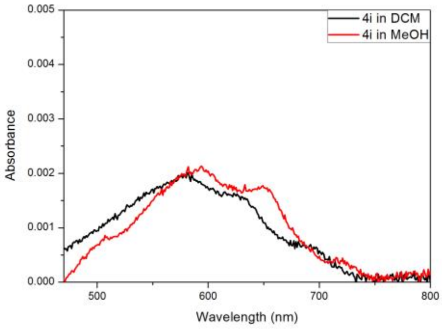
4i in DCM
4i in MeOH
Absorbance
Wavelength (nm)

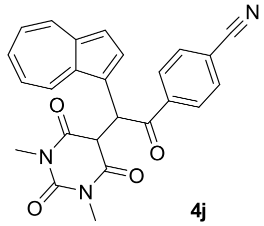

4j

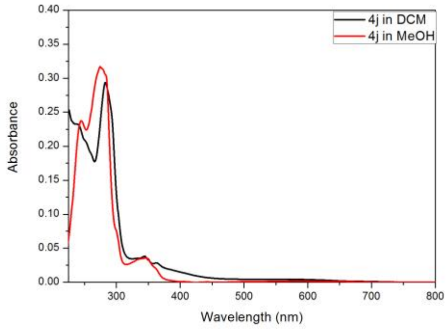
4j in DCM
4j in MeOH
Absorbance
Wavelength (nm)

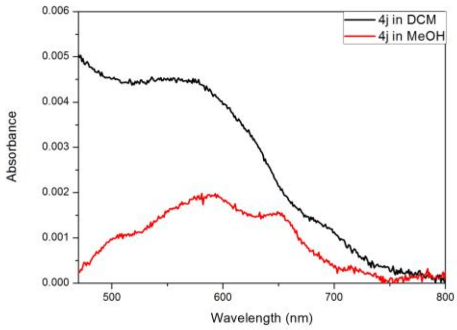
4j in DCM
4j in MeOH
Absorbance
Wavelength (nm)

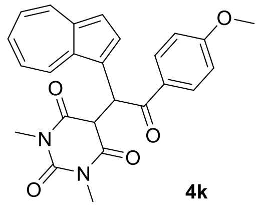

4k

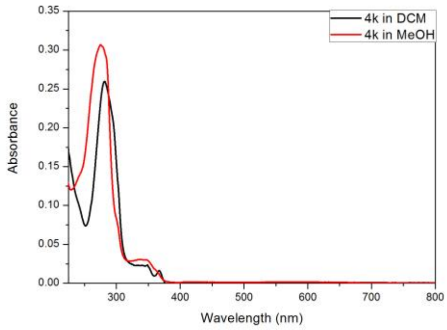
4k in DCM
4k in MeOH
Absorbance
Wavelength (nm)

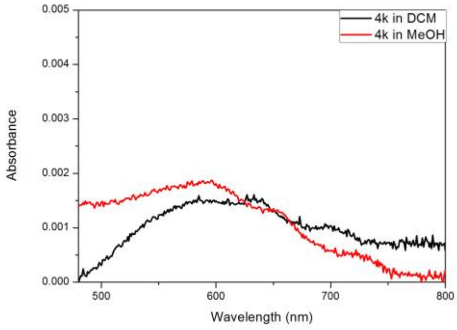
4k in DCM
4k in MeOH
Absorbance
Wavelength (nm)

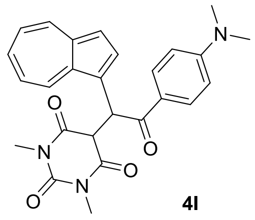
N
O
N
O
O
N
O

4l

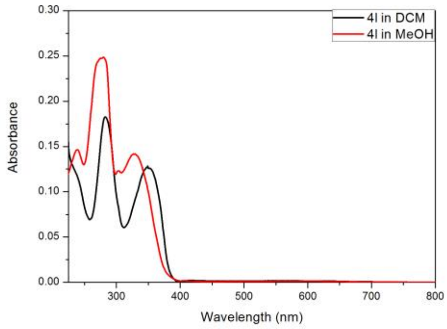
4l in DCM
4l in MeOH
Absorbance
Wavelength (nm)

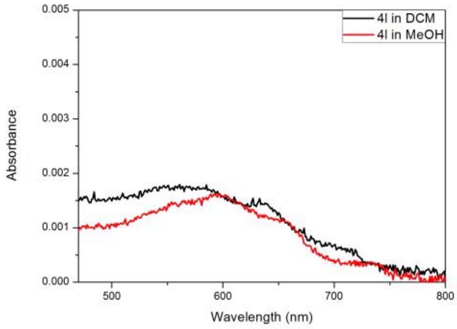
4l in DCM
4l in MeOH
Absorbance
Wavelength (nm)

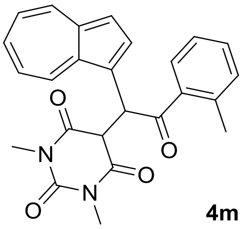
O
N
O
N
O
O

4m

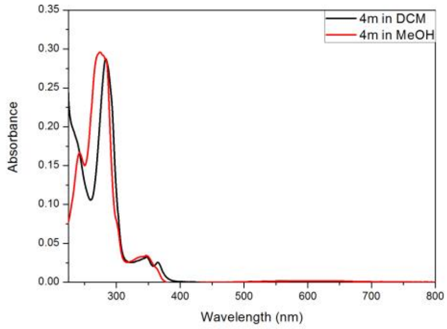
4m in DCM
4m in MeOH
Absorbance
Wavelength (nm)

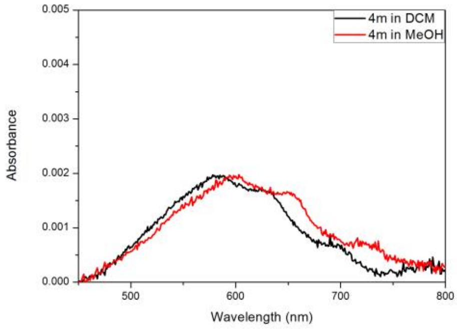
4m in DCM
4m in MeOH
Absorbance
Wavelength (nm)

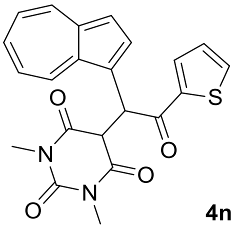
S
O
N
O
N
O
O

4n

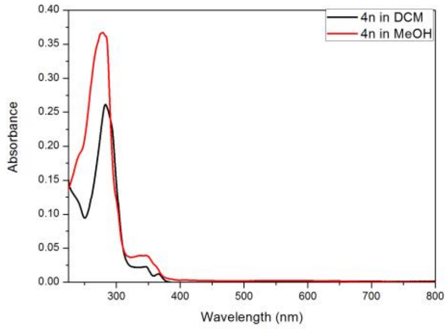
4n in DCM
4n in MeOH
Absorbance
Wavelength (nm)

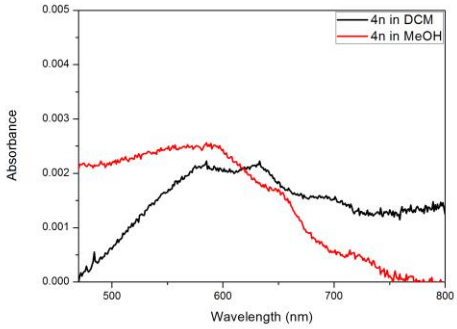
4n in DCM
4n in MeOH
Absorbance
Wavelength (nm)

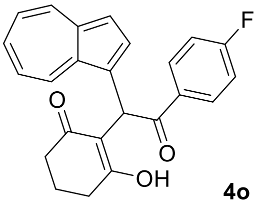
F
O
O
OH

4o

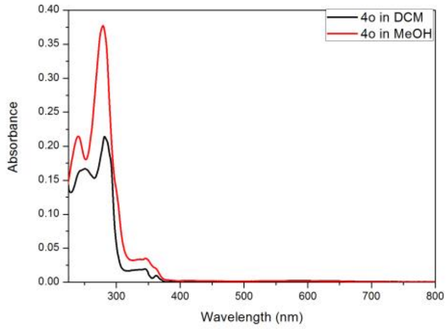
4o in DCM
4o in MeOH
Absorbance
Wavelength (nm)

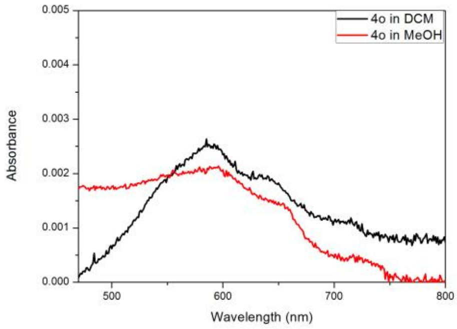
4o in DCM
4o in MeOH
Absorbance
Wavelength (nm)

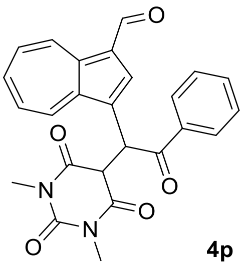

4p

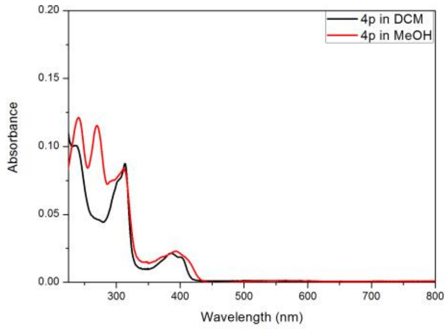
4p in DCM
4p in MeOH
Absorbance
Wavelength (nm)

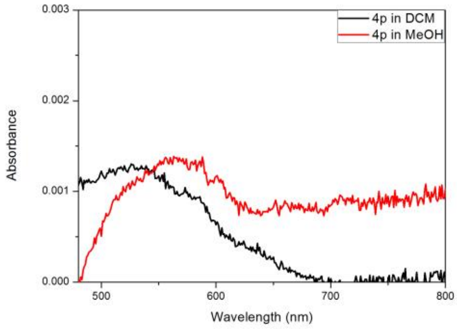
4p in DCM
4p in MeOH
Absorbance
Wavelength (nm)

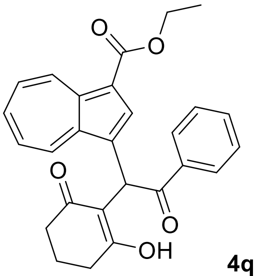

4q

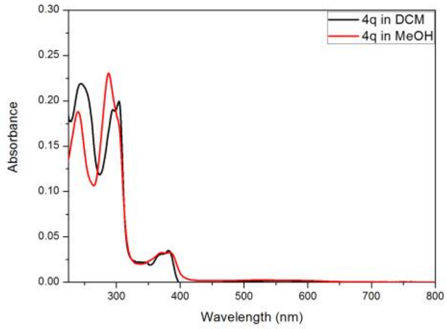
4q in DCM
4q in MeOH
Absorbance
Wavelength (nm)

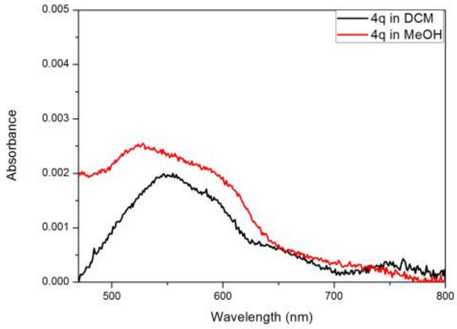
4q in DCM
4q in MeOH
Absorbance
Wavelength (nm)

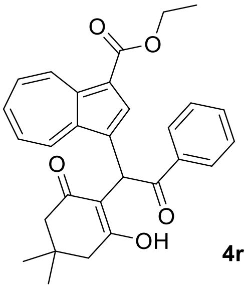

4r

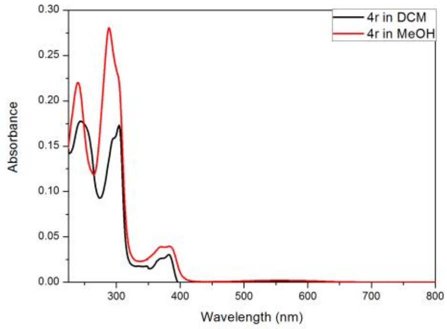
4r in DCM
4r in MeOH
Absorbance
Wavelength (nm)

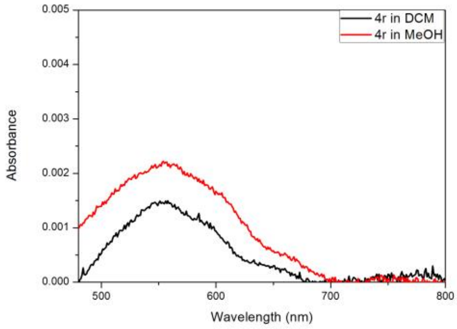
4r in DCM
4r in MeOH
Absorbance
Wavelength (nm)

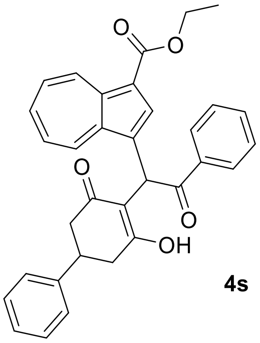

4s

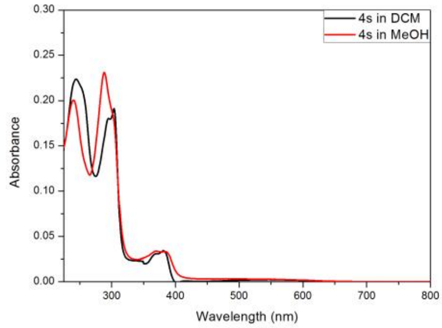
4s in DCM
4s in MeOH
Absorbance
Wavelength (nm)

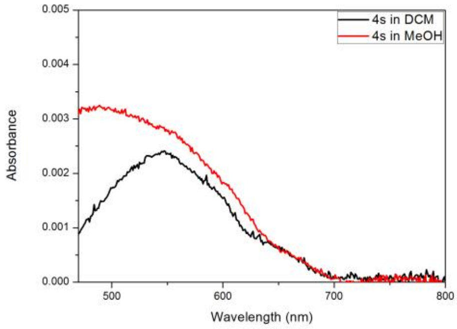
4s in DCM
4s in MeOH
Absorbance
Wavelength (nm)

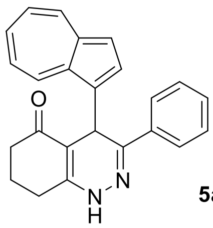

5a

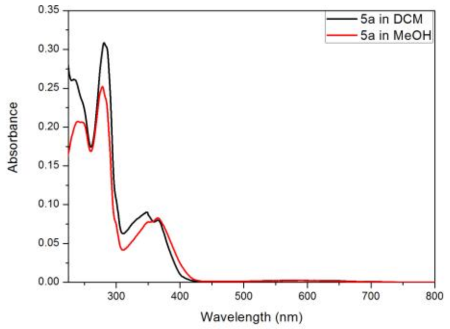
5a in DCM
5a in MeOH
Absorbance
Wavelength (nm)

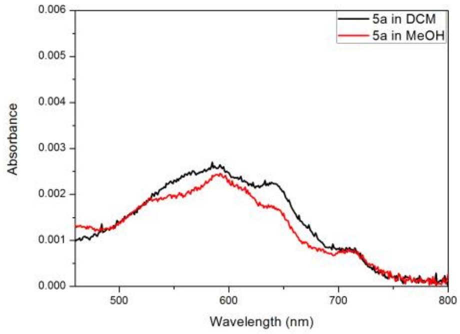
5a in DCM
5a in MeOH
Absorbance
Wavelength (nm)

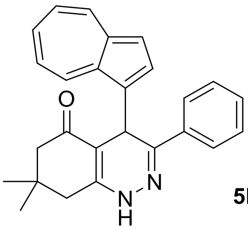

5b

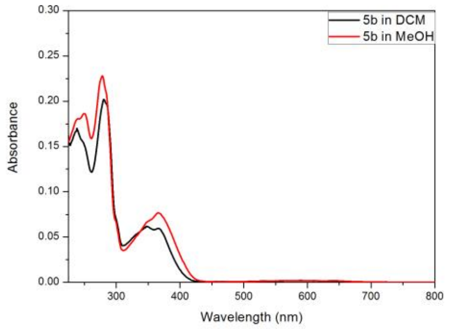
5b in DCM
5b in MeOH
Absorbance
Wavelength (nm)

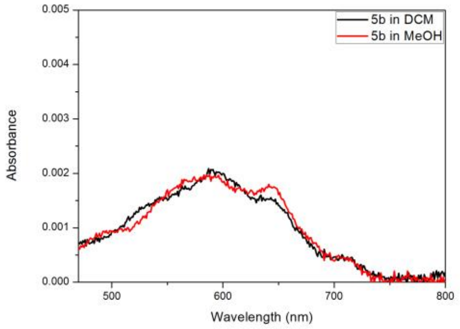
5b in DCM
5b in MeOH
Absorbance
Wavelength (nm)

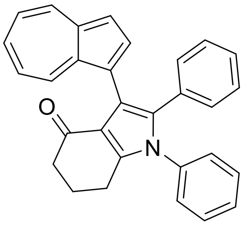

7a

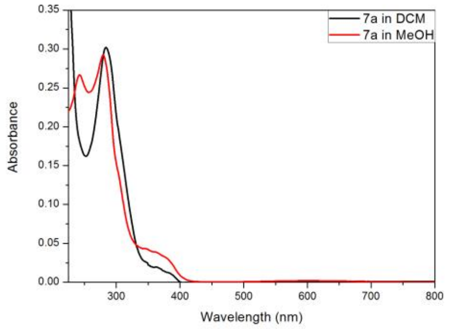
7a in DCM
7a in MeOH
Absorbance
Wavelength (nm)

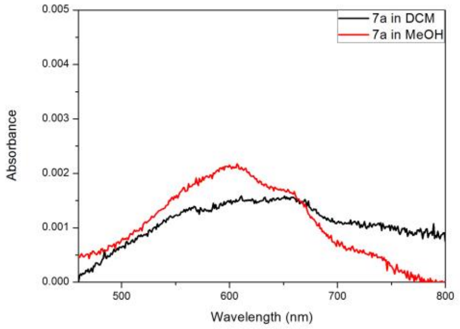
7a in DCM
7a in MeOH
Absorbance
Wavelength (nm)

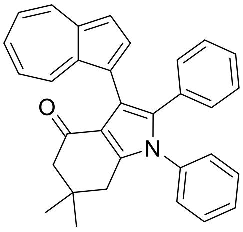

7b

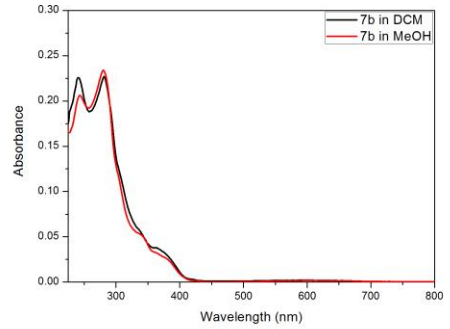
7b in DCM
7b in MeOH
Absorbance
Wavelength (nm)

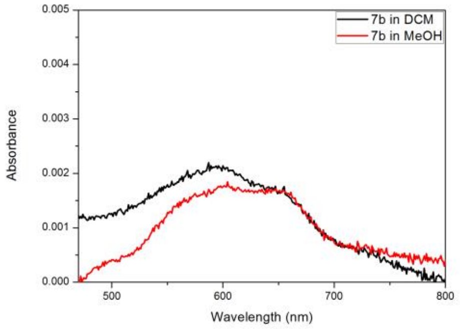
7b in DCM
7b in MeOH
Absorbance
Wavelength (nm)

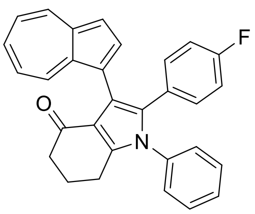 **7c**	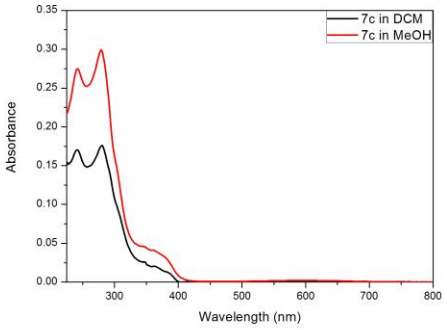 7c in DCM 7c in MeOH Absorbance Wavelength (nm)	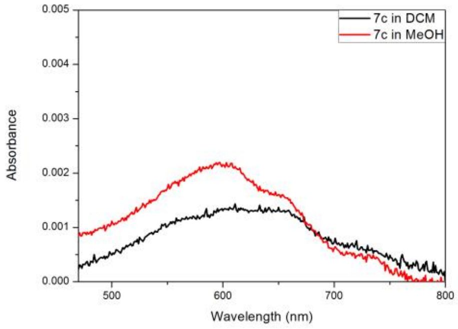 7c in DCM 7c in MeOH Absorbance Wavelength (nm)
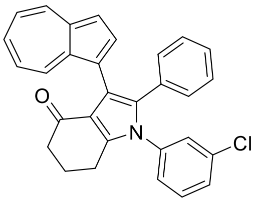 **7d**	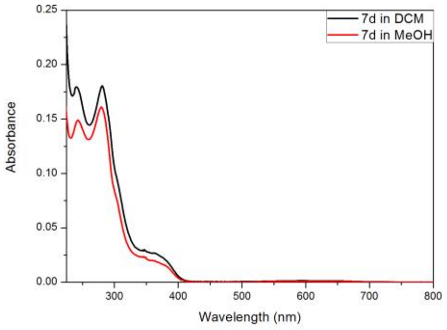 7d in DCM 7d in MeOH Absorbance Wavelength (nm)	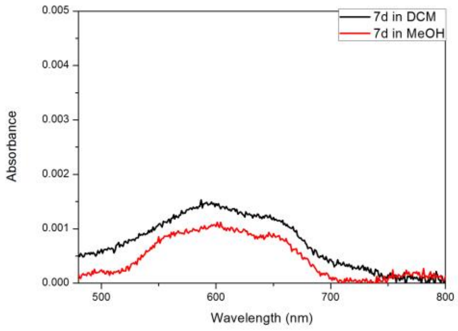 7d in DCM 7d in MeOH Absorbance Wavelength (nm)
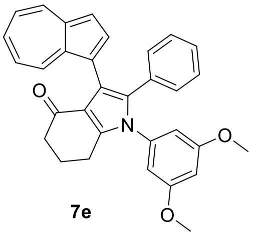 **7e**	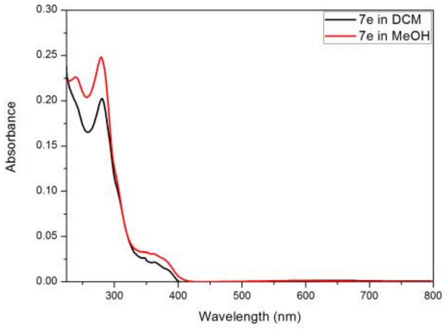 7e in DCM 7e in MeOH Absorbance Wavelength (nm)	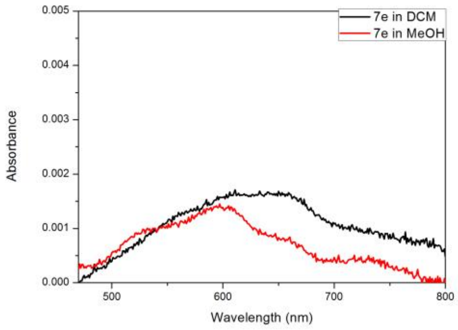 7e in DCM 7e in MeOH Absorbance Wavelength (nm)
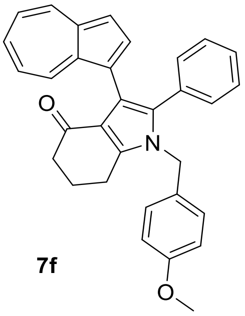 **7f**	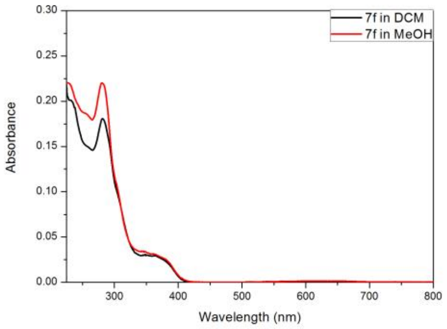 7f in DCM 7f in MeOH Absorbance Wavelength (nm)	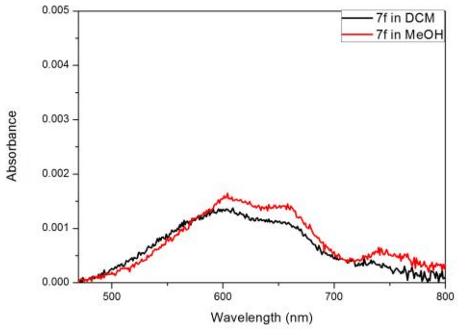 7f in DCM 7f in MeOH Absorbance Wavelength (nm)

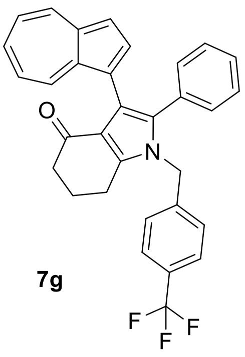 **7g**	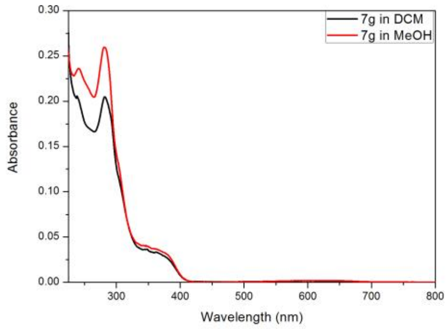 7g in DCM<br>7g in MeOH<br>Absorbance<br>Wavelength (nm)	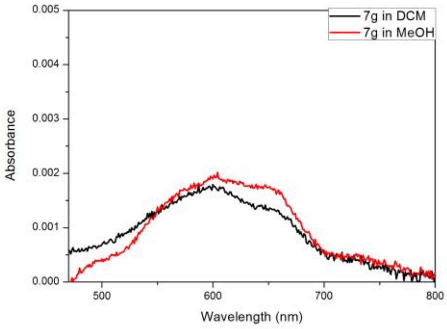 7g in DCM<br>7g in MeOH<br>Absorbance<br>Wavelength (nm)
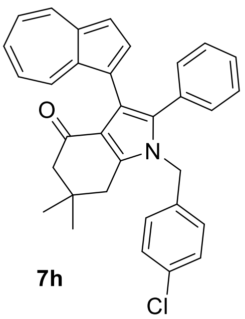 **7h**	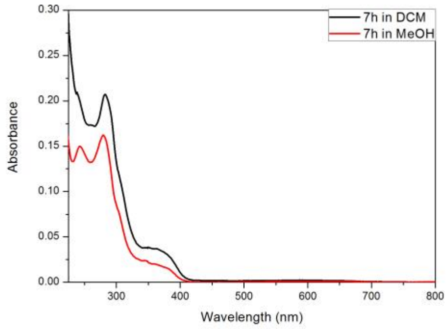 7h in DCM<br>7h in MeOH<br>Absorbance<br>Wavelength (nm)	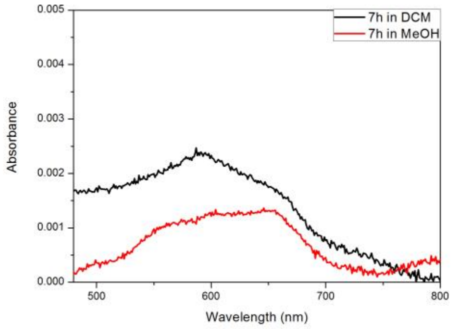 7h in DCM<br>7h in MeOH<br>Absorbance<br>Wavelength (nm)

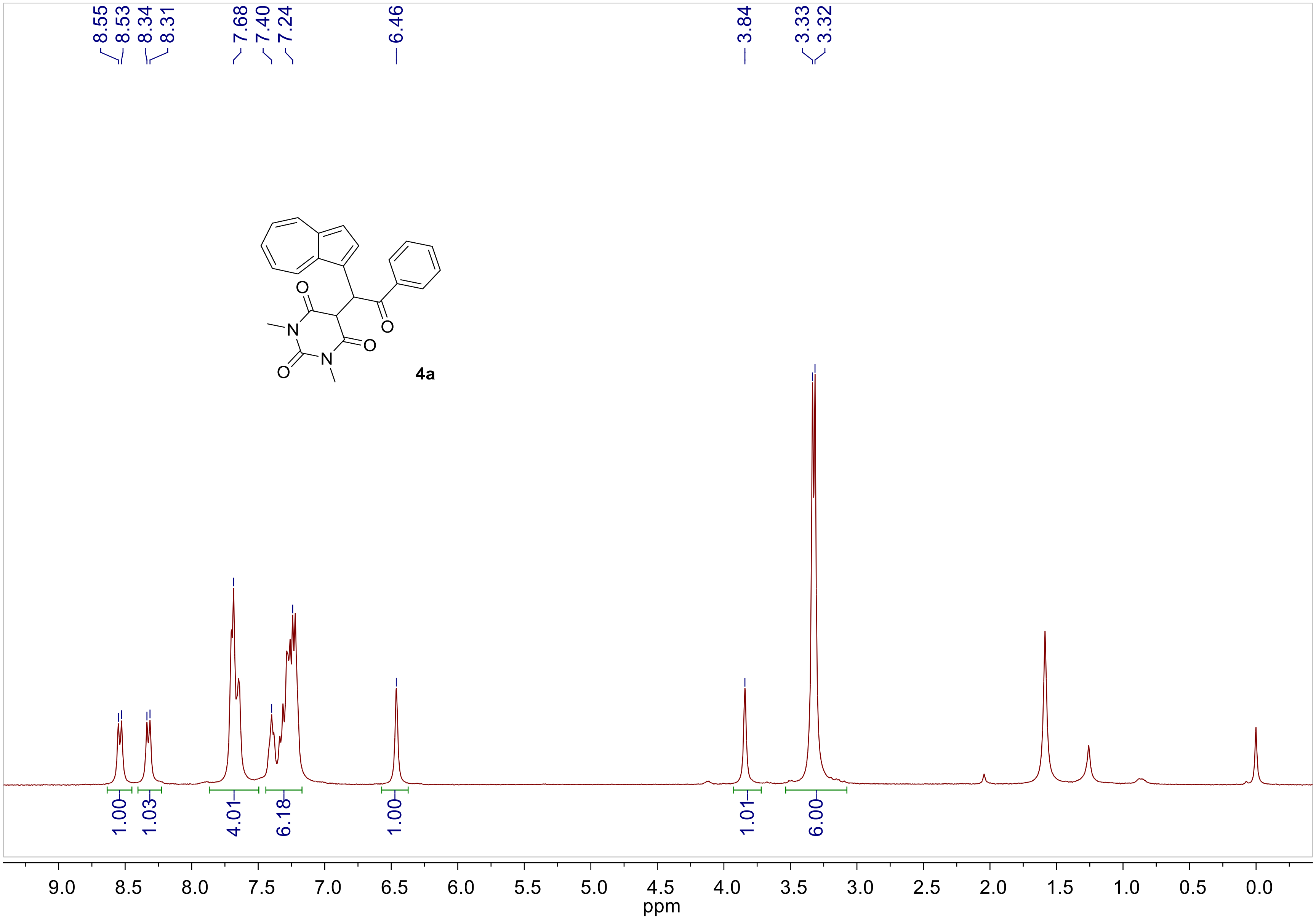
8.55
8.53
8.34
8.31
7.68
7.40
7.24
6.46
3.84
3.33
3.32
O
N
N
O
O
O
4a
1.00
1.03
4.01
6.18
1.00
1.01
6.00
9.0 8.5 8.0 7.5 7.0 6.5 6.0 5.5 5.0 4.5 4.0 3.5 3.0 2.5 2.0 1.5 1.0 0.5 0.0
ppm

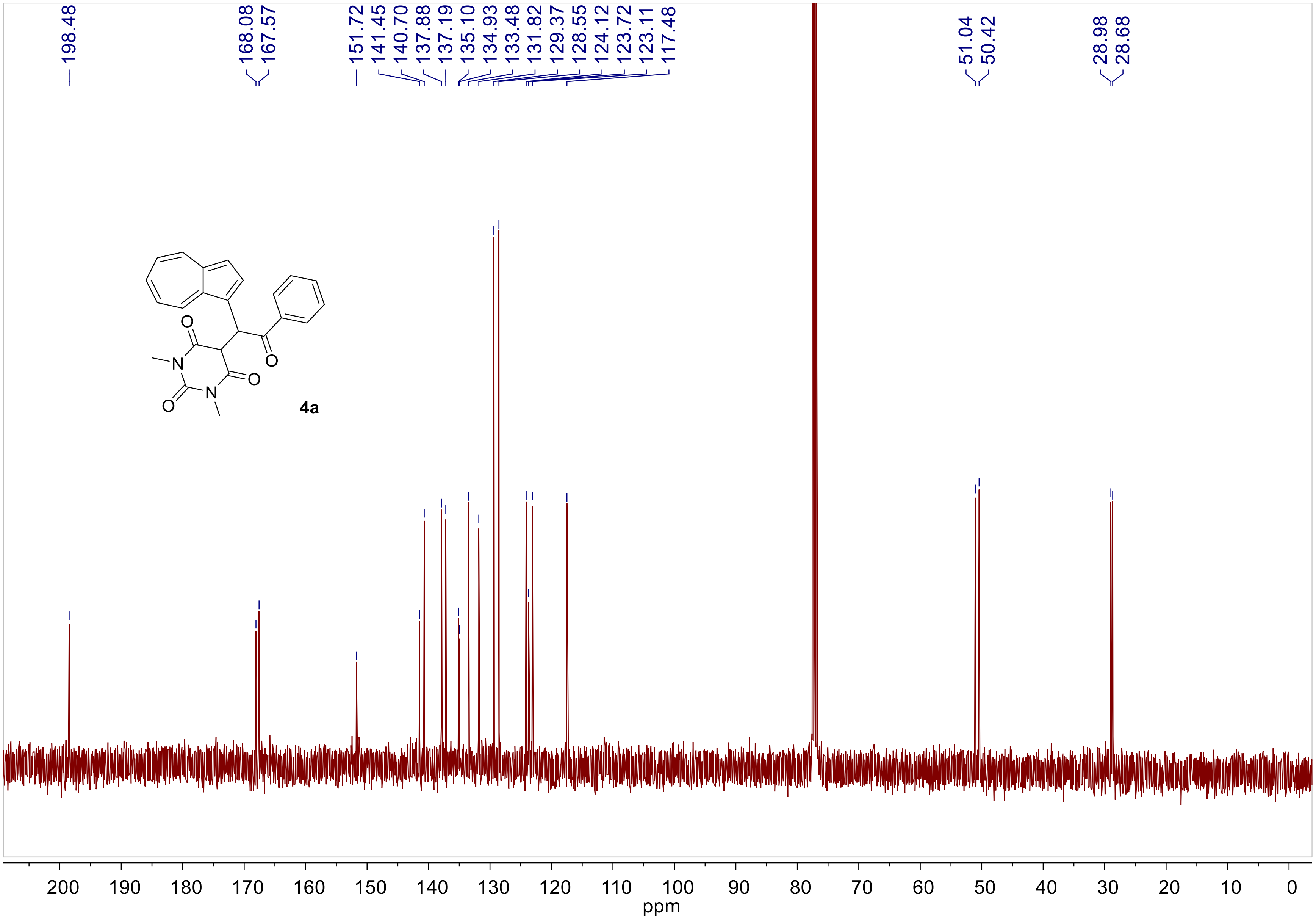
198.48
168.08
167.57
151.72
141.45
140.70
137.88
137.19
135.10
134.93
133.48
131.82
129.37
128.55
124.12
123.72
123.11
117.48
51.04
50.42
28.98
28.68
4a
ppm

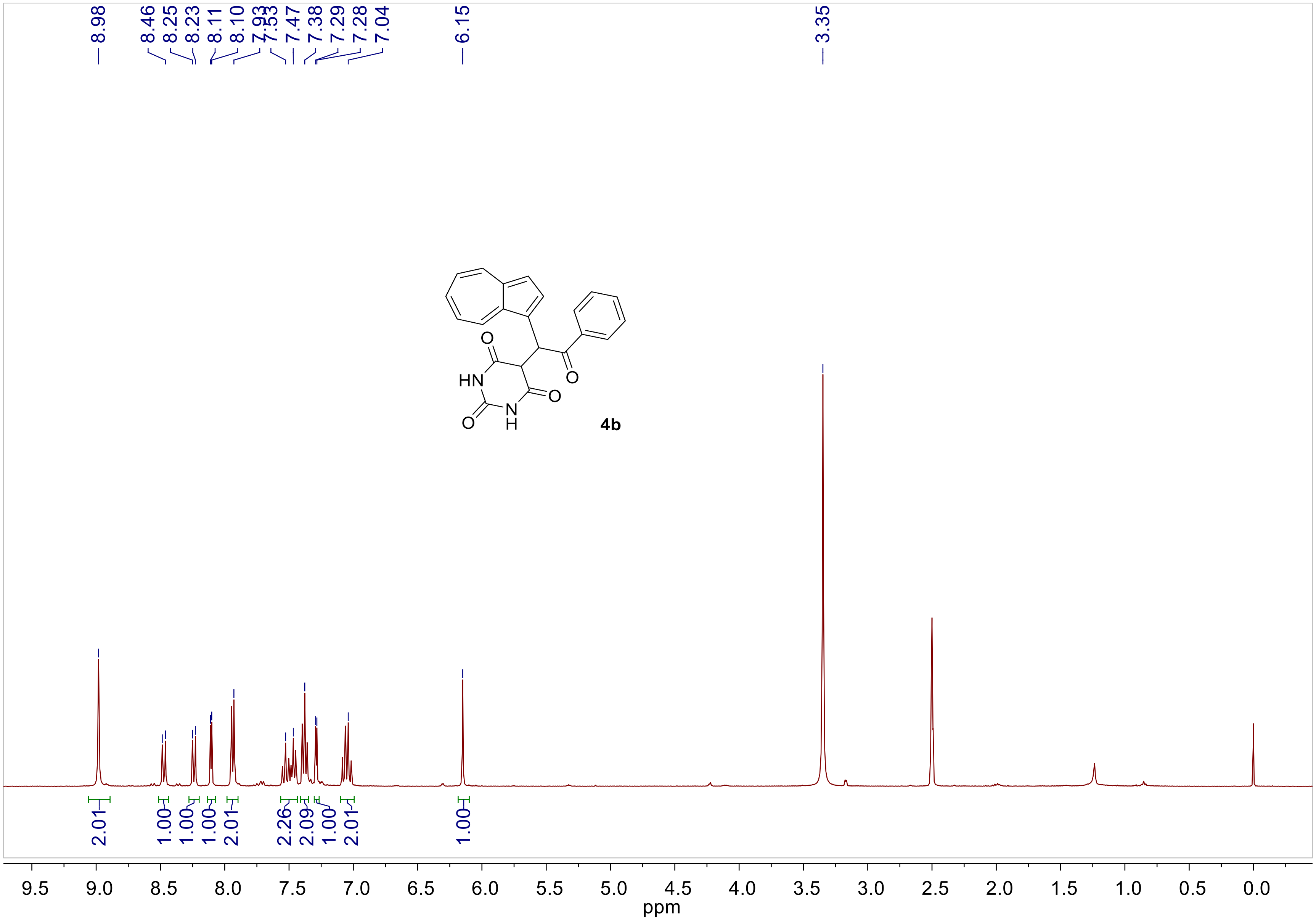

8.98
8.46
8.25
8.23
8.11
8.10
7.93
7.53
7.47
7.38
7.29
7.28
7.04
6.15
3.35
O
HN
O
N
H
O
O
4b
2.01
1.00
1.00
1.00
2.01
2.26
2.09
1.00
2.01
1.00
9.5
9.0
8.5
8.0
7.5
7.0
6.5
6.0
5.5
5.0
4.5
4.0
3.5
3.0
2.5
2.0
1.5
1.0
0.5
0.0
ppm

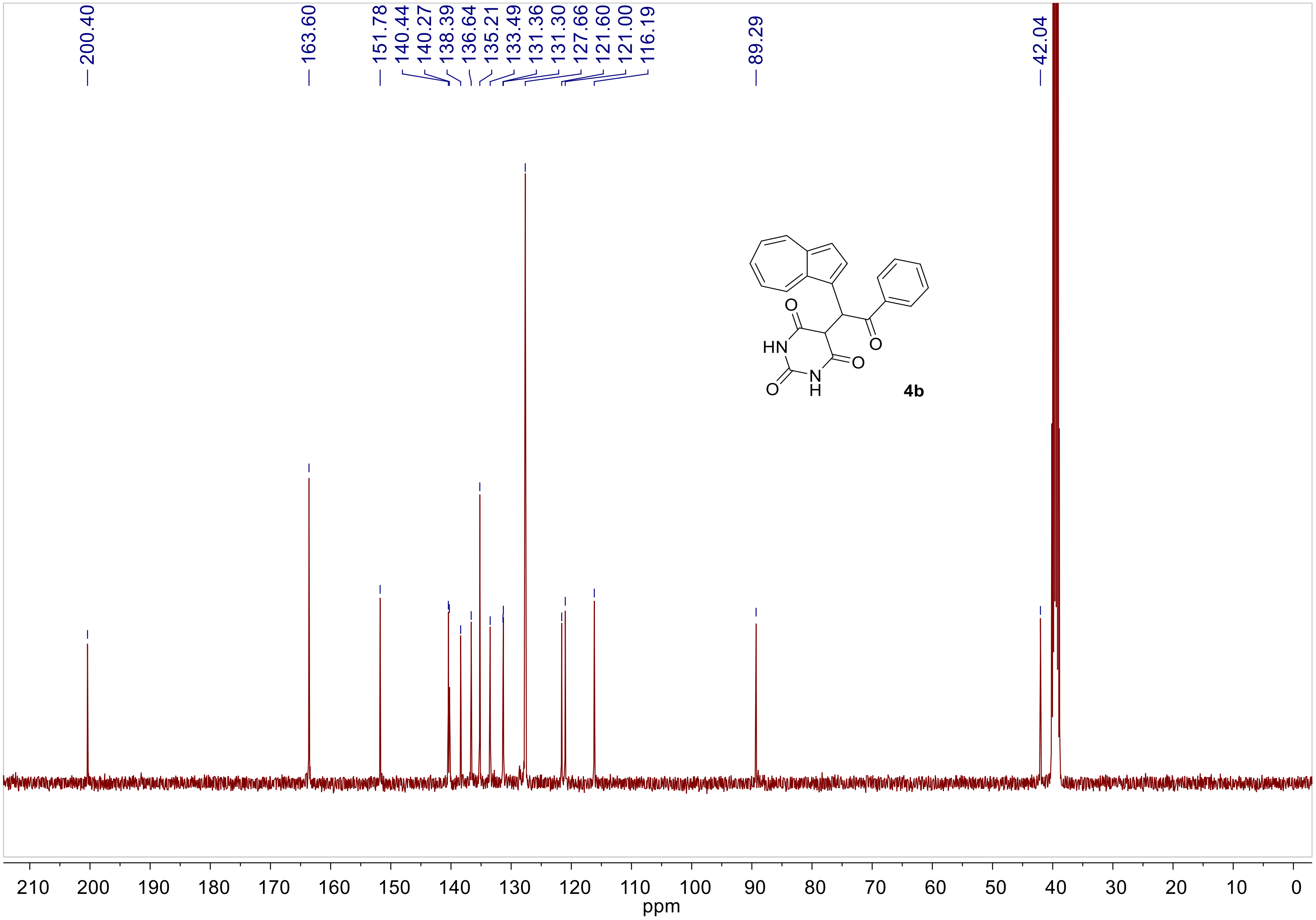

200.40
163.60
151.78
140.44
140.27
138.39
136.64
135.21
133.49
131.36
131.30
127.66
121.60
121.00
116.19
89.29
42.04
HN
O
N
H
4b
210 200 190 180 170 160 150 140 130 120 110 100 90 80 70 60 50 40 30 20 10 0
ppm

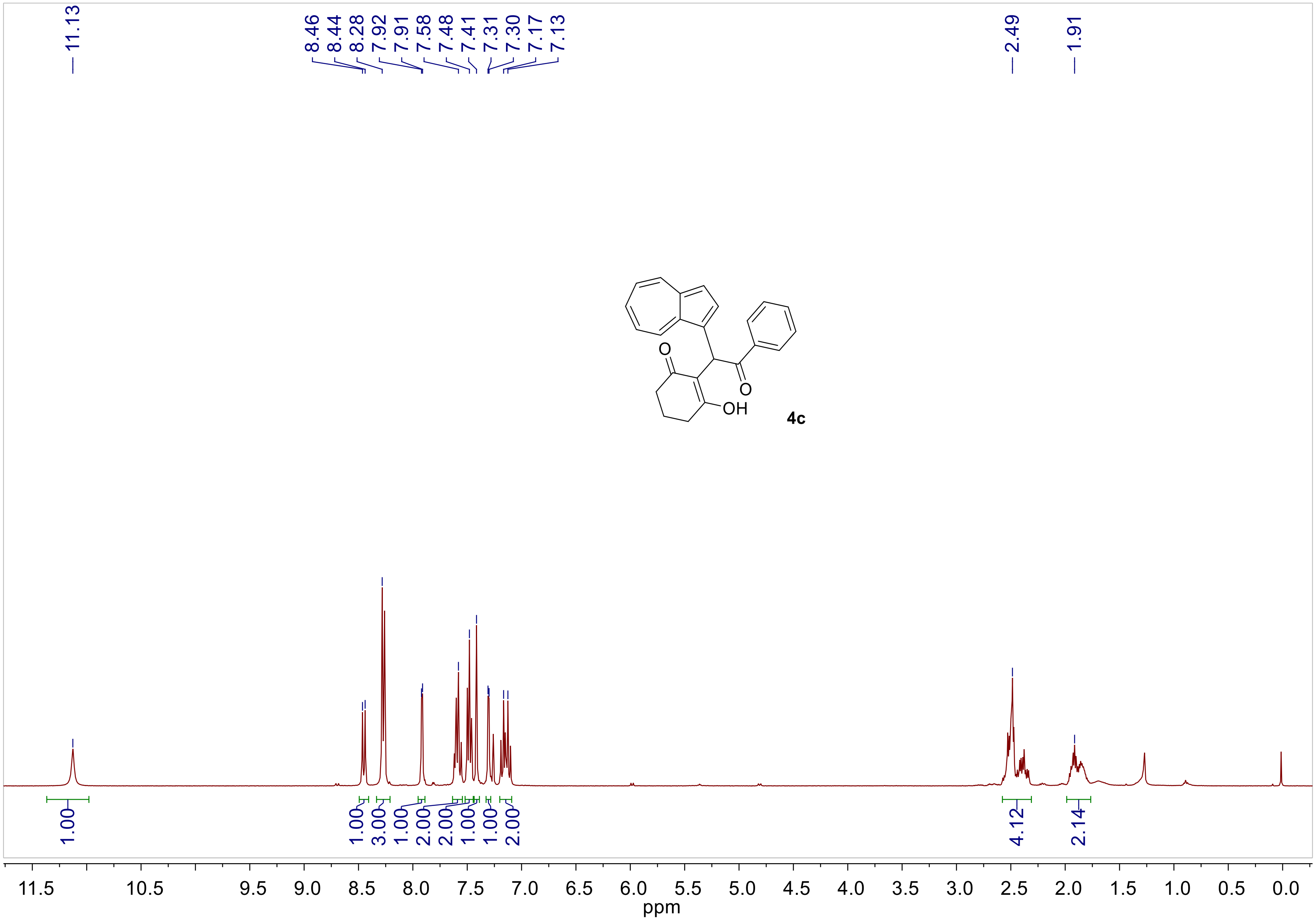
11.13
8.46
8.44
8.28
7.92
7.91
7.58
7.48
7.41
7.31
7.30
7.17
7.13
2.49
1.91
O
O
OH
4c
1.00
1.00
3.00
1.00
2.00
2.00
1.00
1.00
2.00
4.12
2.14
11.5
10.5
9.5
9.0
8.5
8.0
7.5
7.0
6.5
6.0
5.5
5.0
4.5
4.0
3.5
3.0
2.5
2.0
1.5
1.0
0.5
0.0
ppm

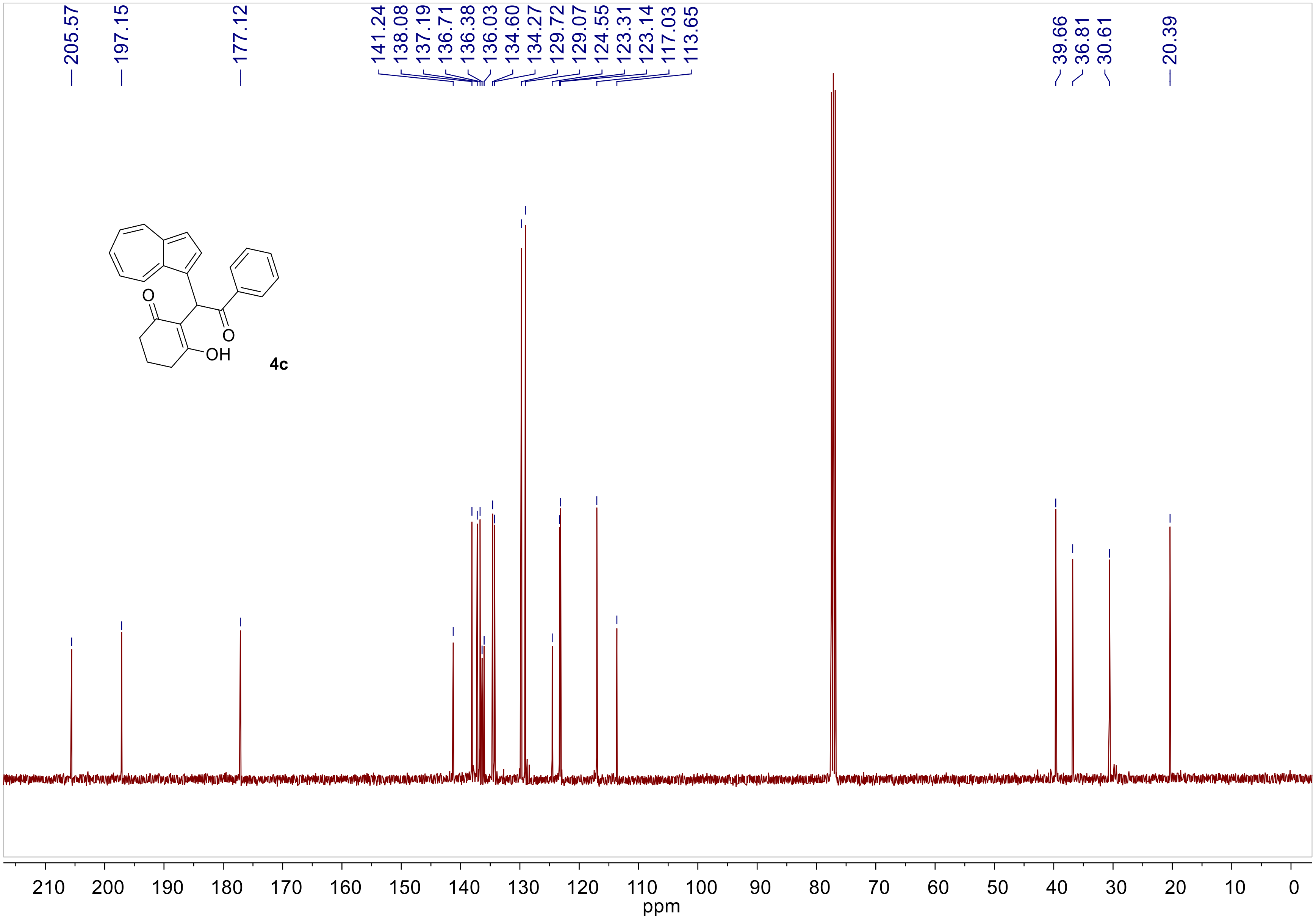
205.57
197.15
177.12
141.24
138.08
137.19
136.71
136.38
136.03
134.60
134.27
129.72
129.07
124.55
123.31
123.14
117.03
113.65
39.66
36.81
30.61
20.39
O
O
OH
4c
210 200 190 180 170 160 150 140 130 120 110 100 90 80 70 60 50 40 30 20 10 0
ppm

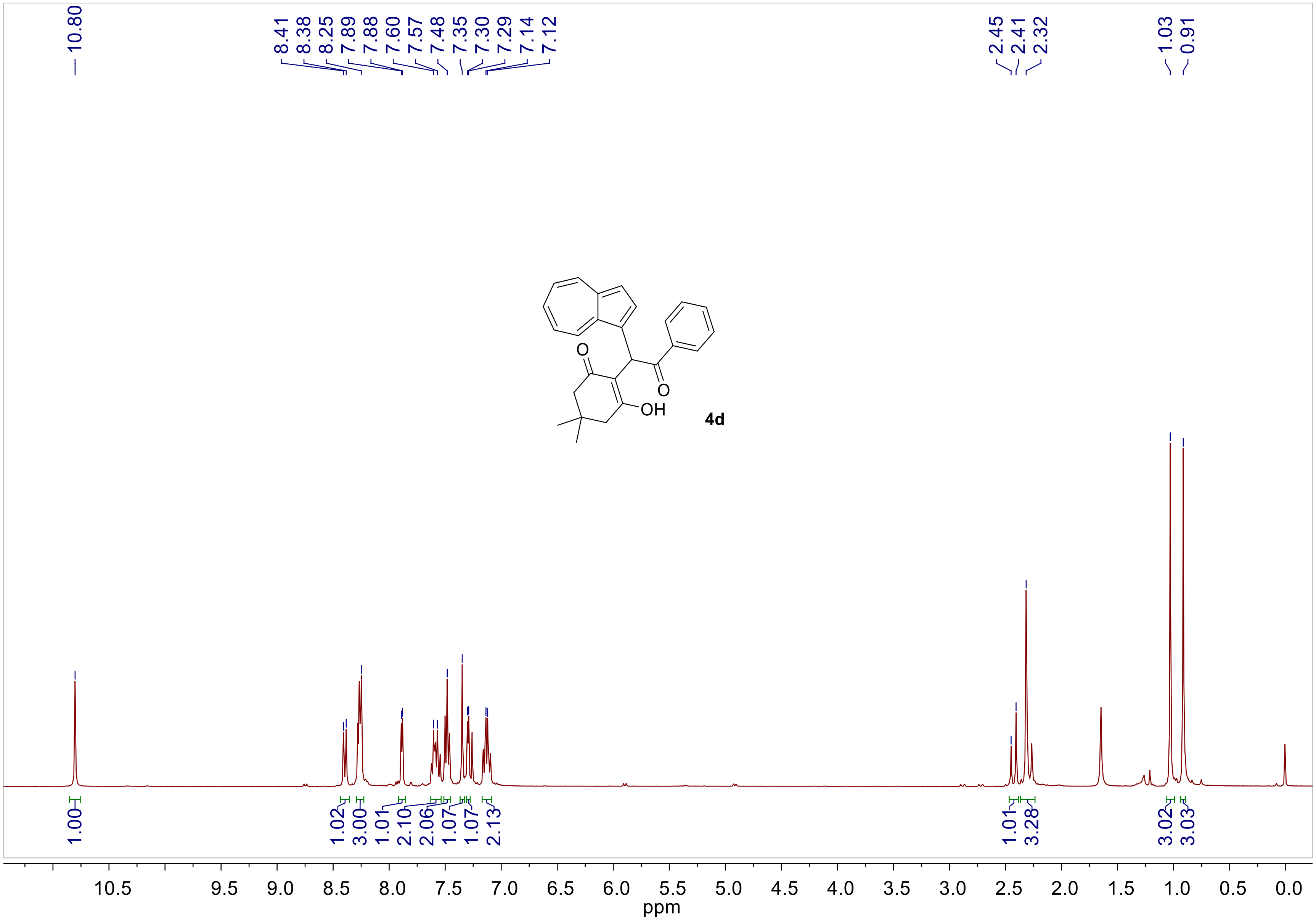
10.80
8.41 8.38 8.25 7.89 7.88 7.60 7.57 7.48 7.35 7.30 7.29 7.14 7.12
2.45 2.41 2.32
1.03 0.91
O
O
OH
4d
1.00 1.02 3.00 1.01 2.10 2.06 1.07 1.07 2.13
1.01 3.28
3.02 3.03
10.5 9.5 9.0 8.5 8.0 7.5 7.0 6.5 6.0 5.5 5.0 4.5 4.0 3.5 3.0 2.5 2.0 1.5 1.0 0.5 0.0
ppm

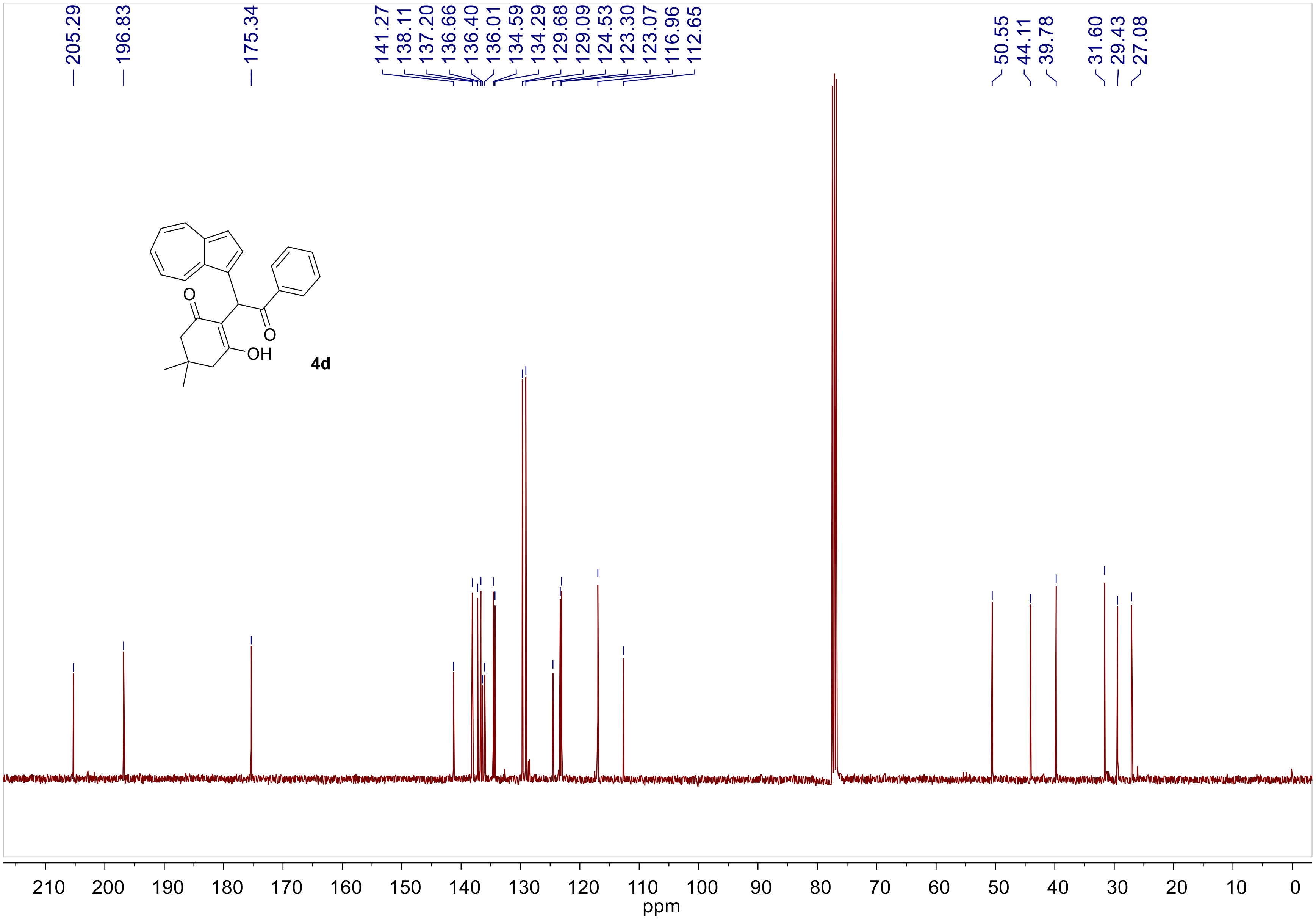
205.29
196.83
175.34
141.27
138.11
137.20
136.66
136.40
136.01
134.59
134.29
129.68
129.09
124.53
123.30
123.07
116.96
112.65
50.55
44.11
39.78
31.60
29.43
27.08
O
O
OH
4d
210
200
190
180
170
160
150
140
130
120
110
100
90
80
70
60
50
40
30
20
10
0
ppm

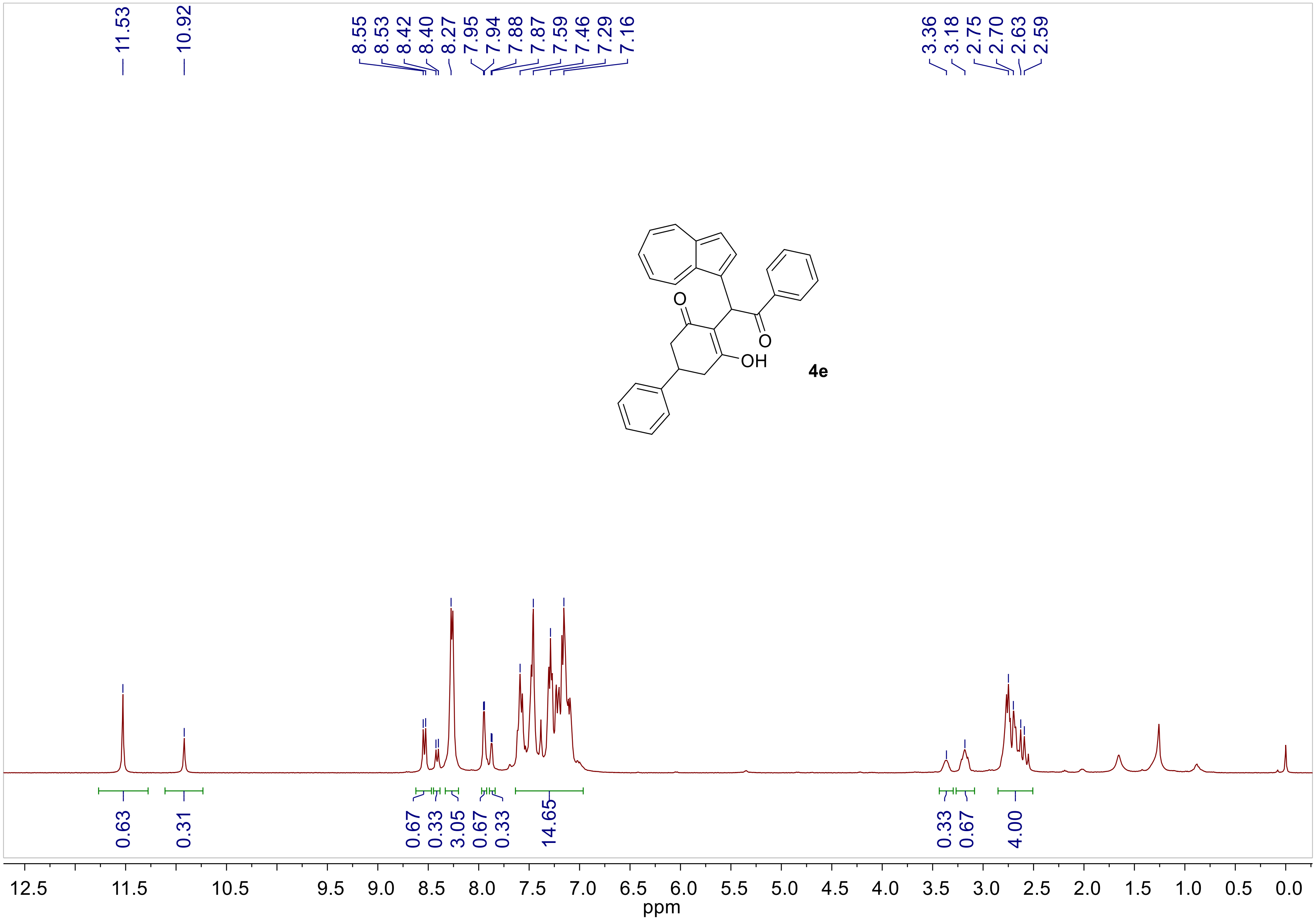
11.53
10.92
8.55
8.53
8.42
8.40
8.27
7.95
7.94
7.88
7.87
7.59
7.46
7.29
7.16
3.36
3.18
2.75
2.70
2.63
2.59
O
O
OH
4e
0.63
0.31
0.67
0.33
3.05
0.67
0.33
14.65
0.33
0.67
4.00
12.5
11.5
10.5
9.5
9.0
8.5
8.0
7.5
7.0
6.5
6.0
5.5
5.0
4.5
4.0
3.5
3.0
2.5
2.0
1.5
1.0
0.5
0.0
ppm

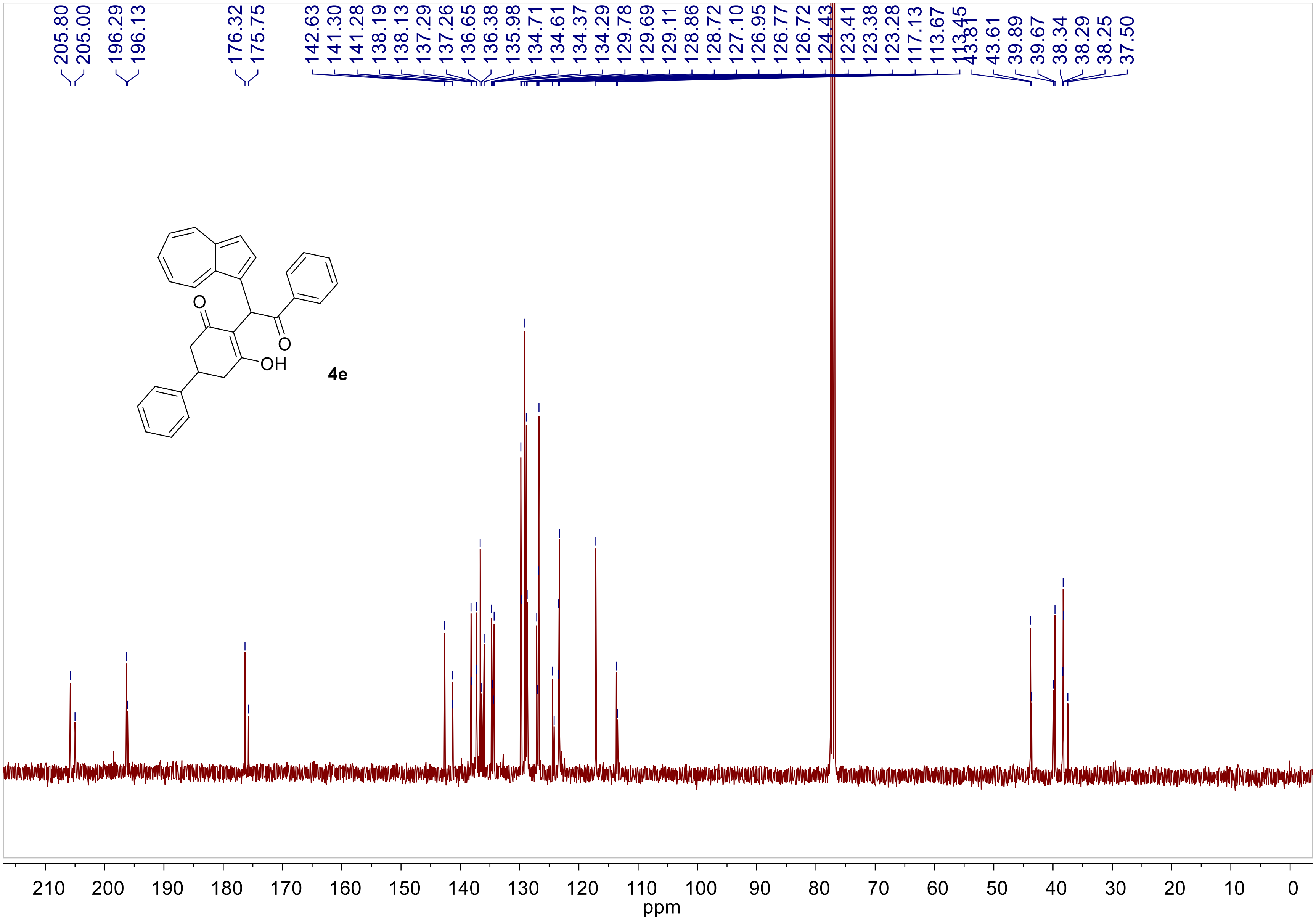
205.80
205.00
196.29
196.13
176.32
175.75
142.63
141.30
141.28
138.19
138.13
137.29
137.26
136.65
136.38
135.98
134.71
134.61
134.37
134.29
129.78
129.69
129.11
128.86
128.72
127.10
126.95
126.77
126.72
124.43
123.41
123.38
123.28
117.13
113.67
113.45
43.81
43.61
39.89
39.67
38.34
38.29
38.25
37.50
O
O
OH
4e
210 200 190 180 170 160 150 140 130 120 110 100 90 80 70 60 50 40 30 20 10 0
ppm

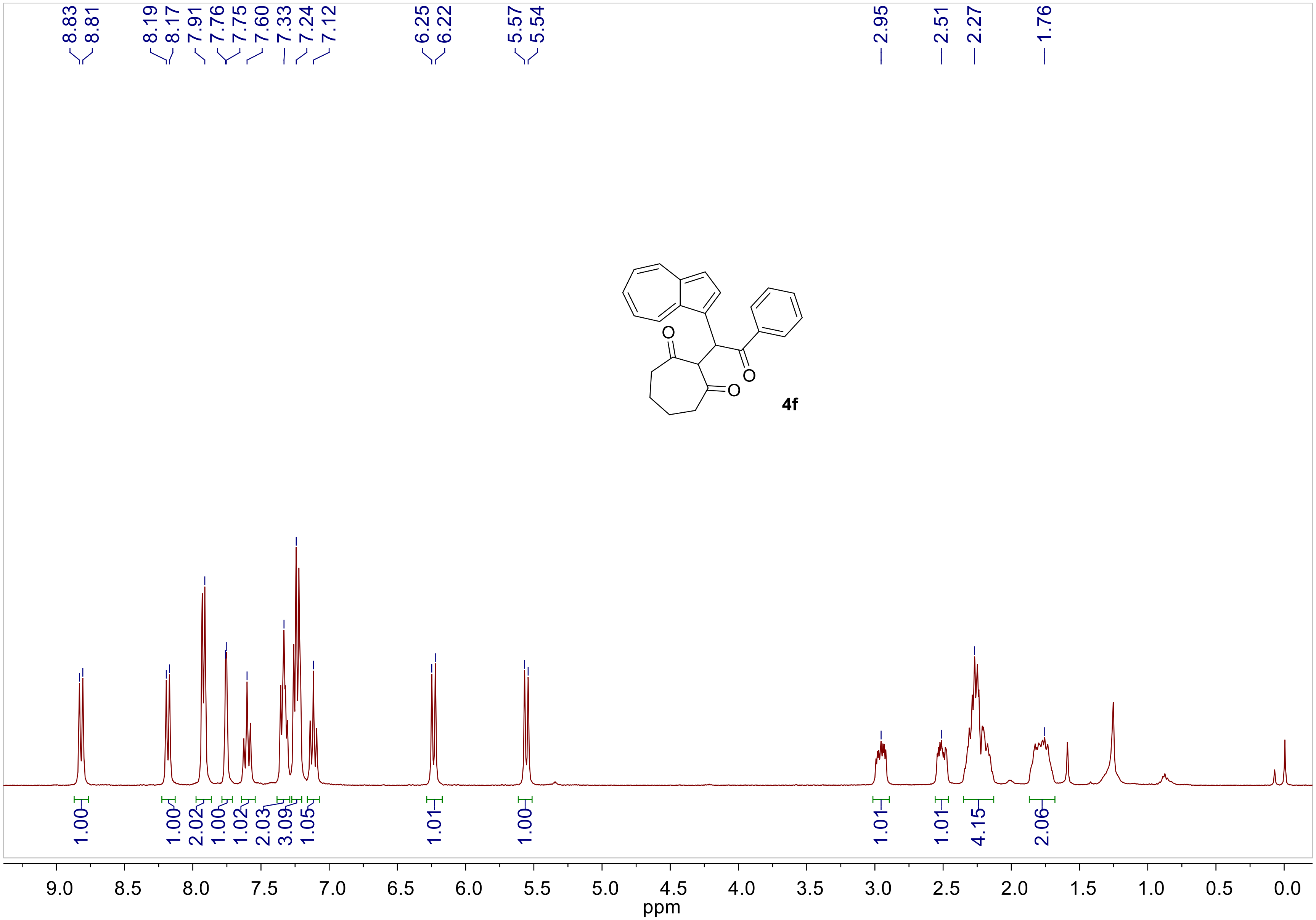
8.83
8.81
8.19
8.17
7.91
7.76
7.75
7.60
7.33
7.24
7.12
6.25
6.22
5.57
5.54
2.95
2.51
2.27
1.76
4f
1.00
1.00
2.02
1.00
1.02
2.03
3.09
1.05
1.01
1.00
1.01
1.01
4.15
2.06
9.0
8.5
8.0
7.5
7.0
6.5
6.0
5.5
5.0
4.5
4.0
3.5
3.0
2.5
2.0
1.5
1.0
0.5
0.0
ppm

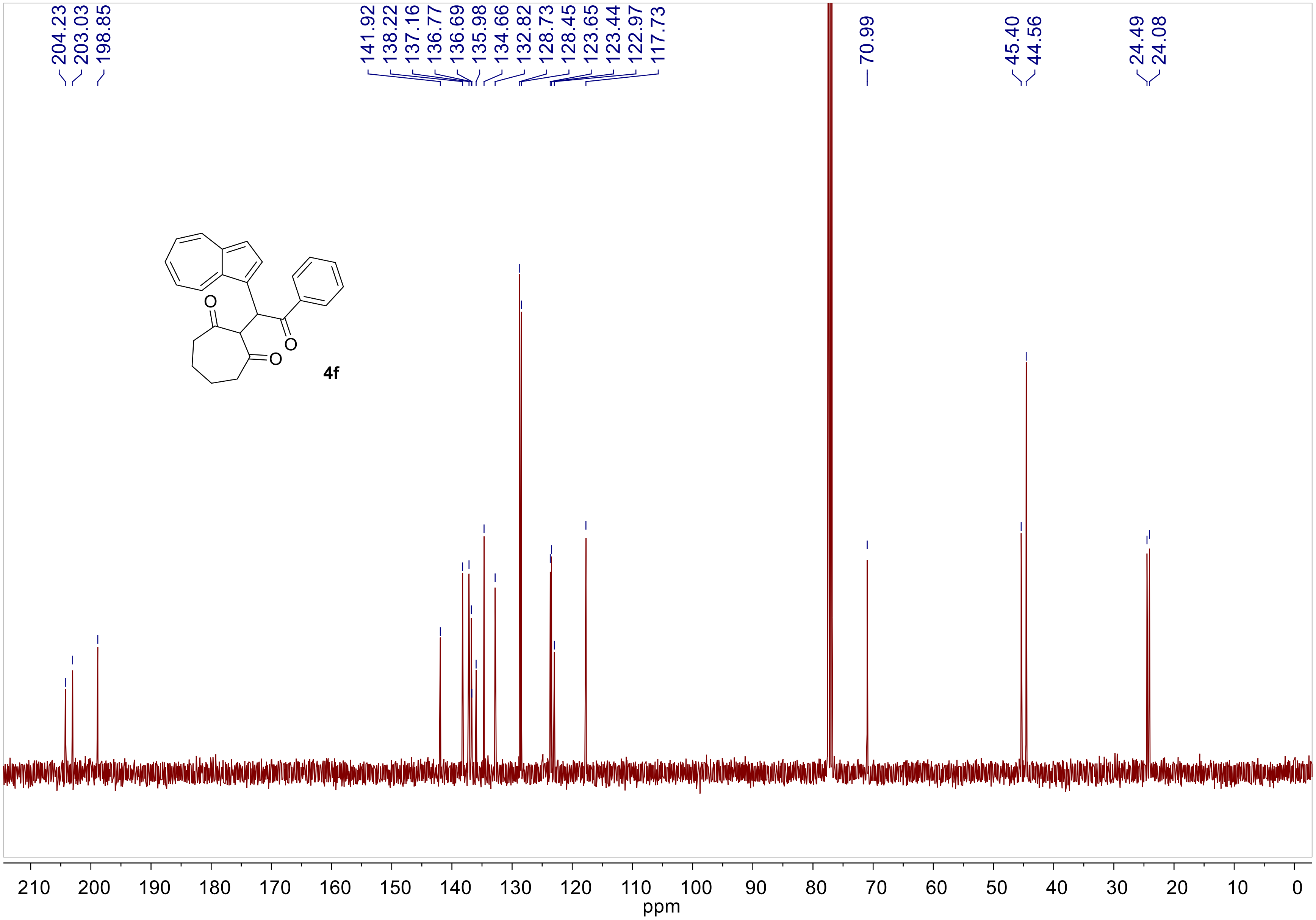
204.23
203.03
198.85
141.92
138.22
137.16
136.77
136.69
135.98
134.66
132.82
128.73
128.45
123.65
123.44
122.97
117.73
70.99
45.40
44.56
24.49
24.08
4f
210 200 190 180 170 160 150 140 130 120 110 100 90 80 70 60 50 40 30 20 10 0
ppm

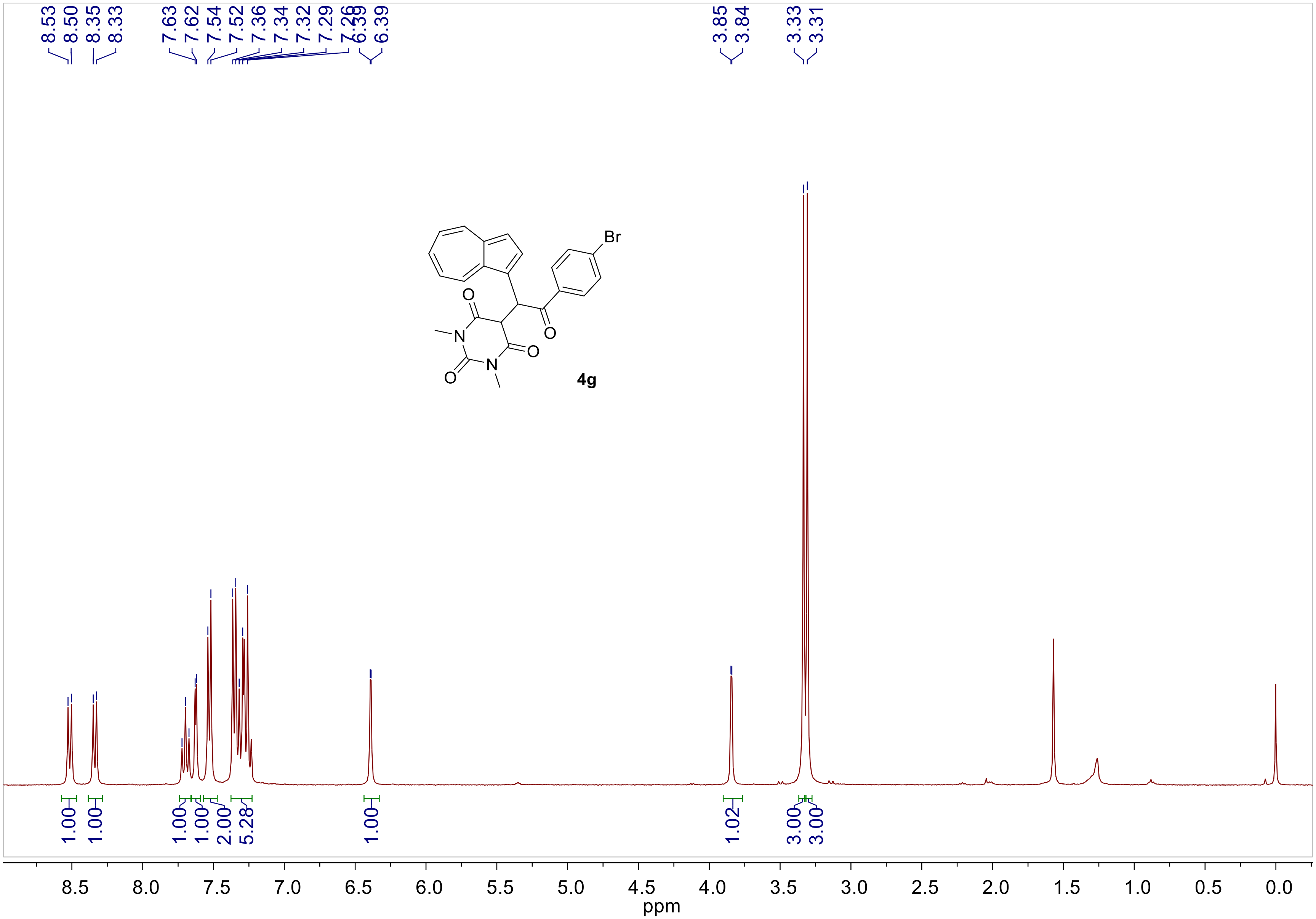
8.53
8.50
8.35
8.33
7.63
7.62
7.54
7.52
7.36
7.34
7.32
7.29
7.26
6.39
6.39
3.85
3.84
3.33
3.31
Br
O
O
N
N
O
O
4g
1.00
1.00
1.00
1.00
2.00
5.28
1.00
1.02
3.00
3.00
8.5
8.0
7.5
7.0
6.5
6.0
5.5
5.0
4.5
4.0
3.5
3.0
2.5
2.0
1.5
1.0
0.5
0.0
ppm

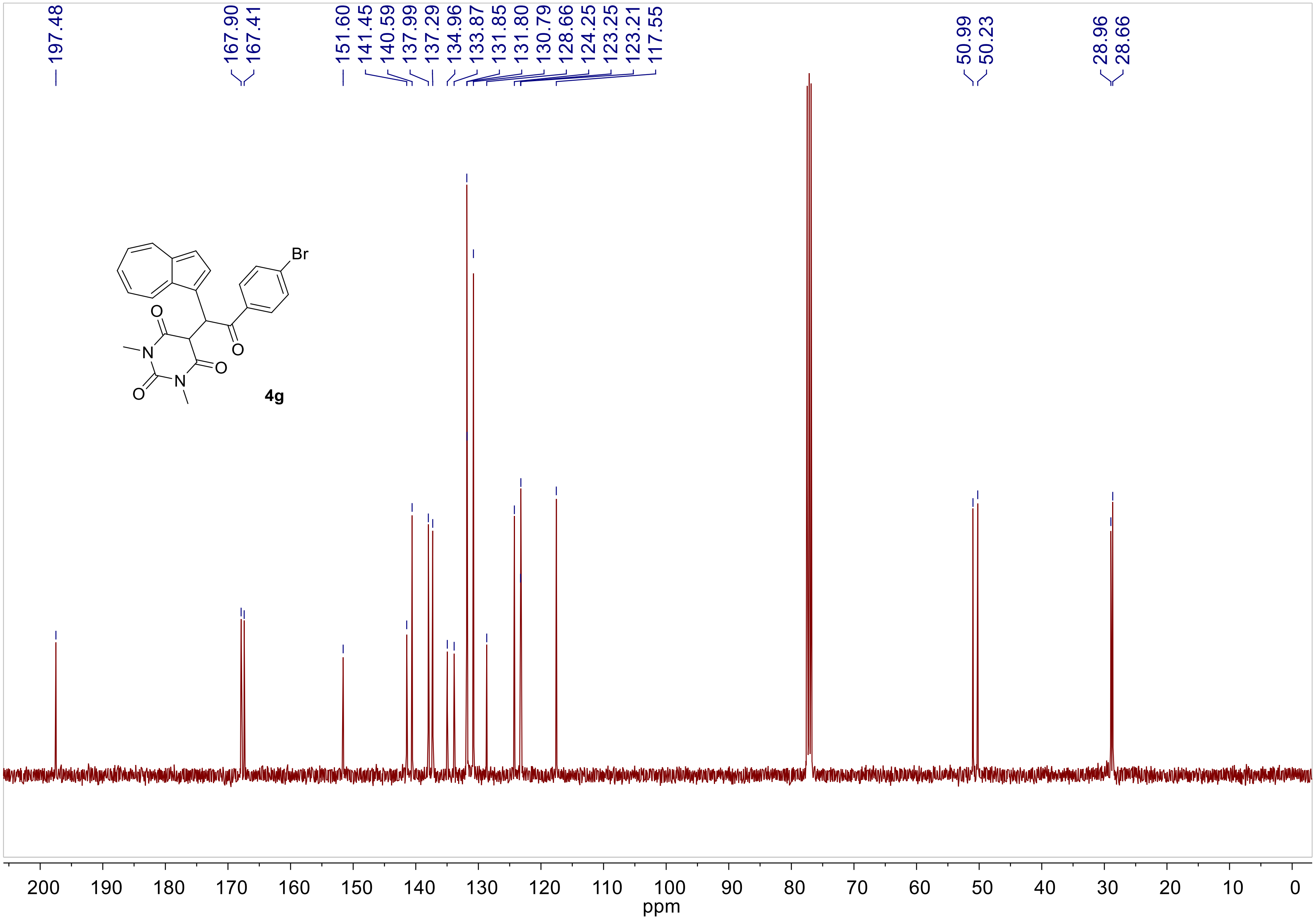
197.48
167.90
167.41
151.60
141.45
140.59
137.99
137.29
134.96
133.87
131.85
131.80
130.79
128.66
124.25
123.25
123.21
117.55
50.99
50.23
28.96
28.66
Br
O
N
4g
200 190 180 170 160 150 140 130 120 110 100 90 80 70 60 50 40 30 20 10 0
ppm

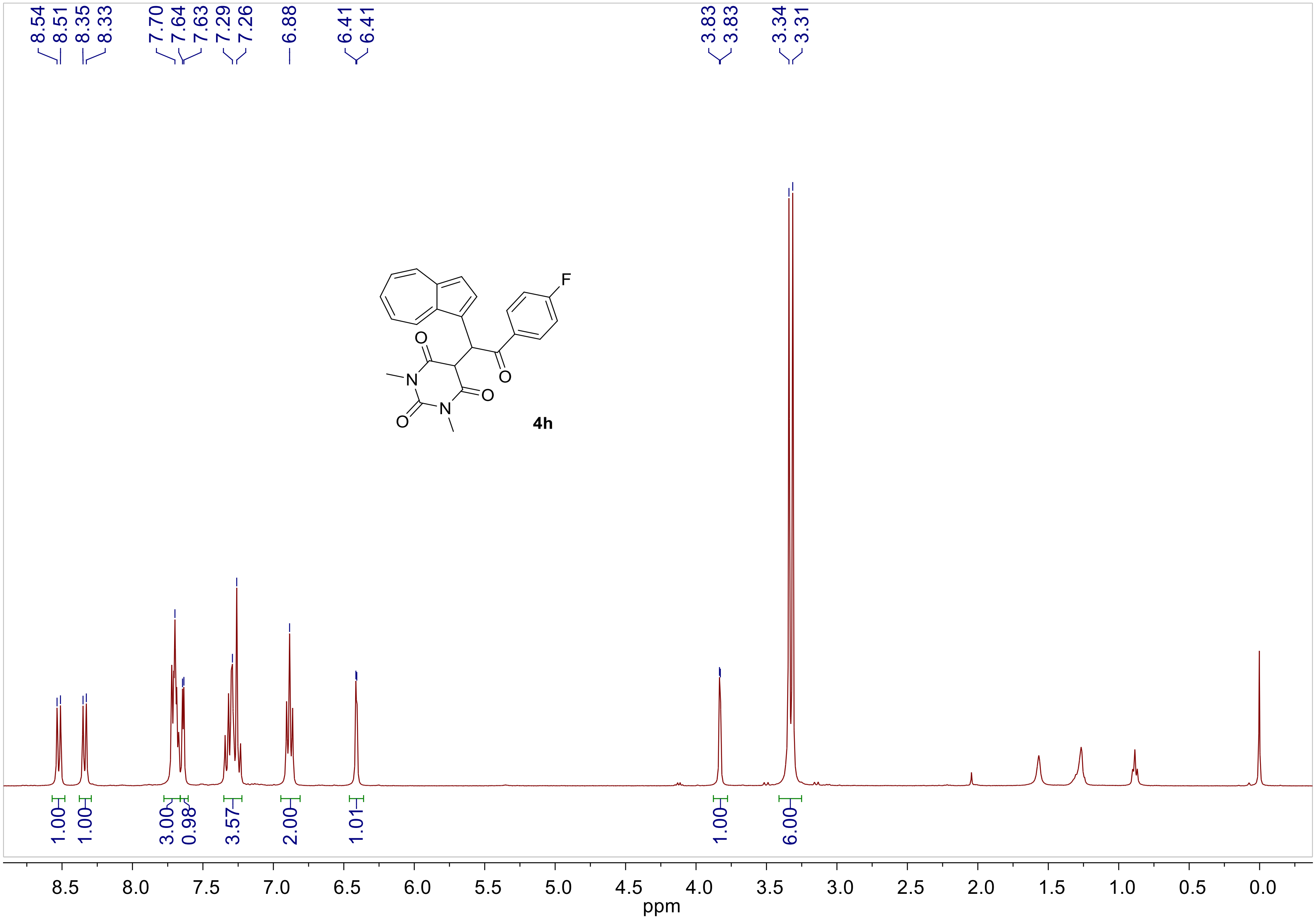
8.54
8.51
8.35
8.33
7.70
7.64
7.63
7.29
7.26
6.88
6.41
6.41
3.83
3.83
3.34
3.31
F
O
N
4h
1.00
1.00
3.00
0.98
3.57
2.00
1.01
1.00
6.00
8.5 8.0 7.5 7.0 6.5 6.0 5.5 5.0 4.5 4.0 3.5 3.0 2.5 2.0 1.5 1.0 0.5 0.0
ppm

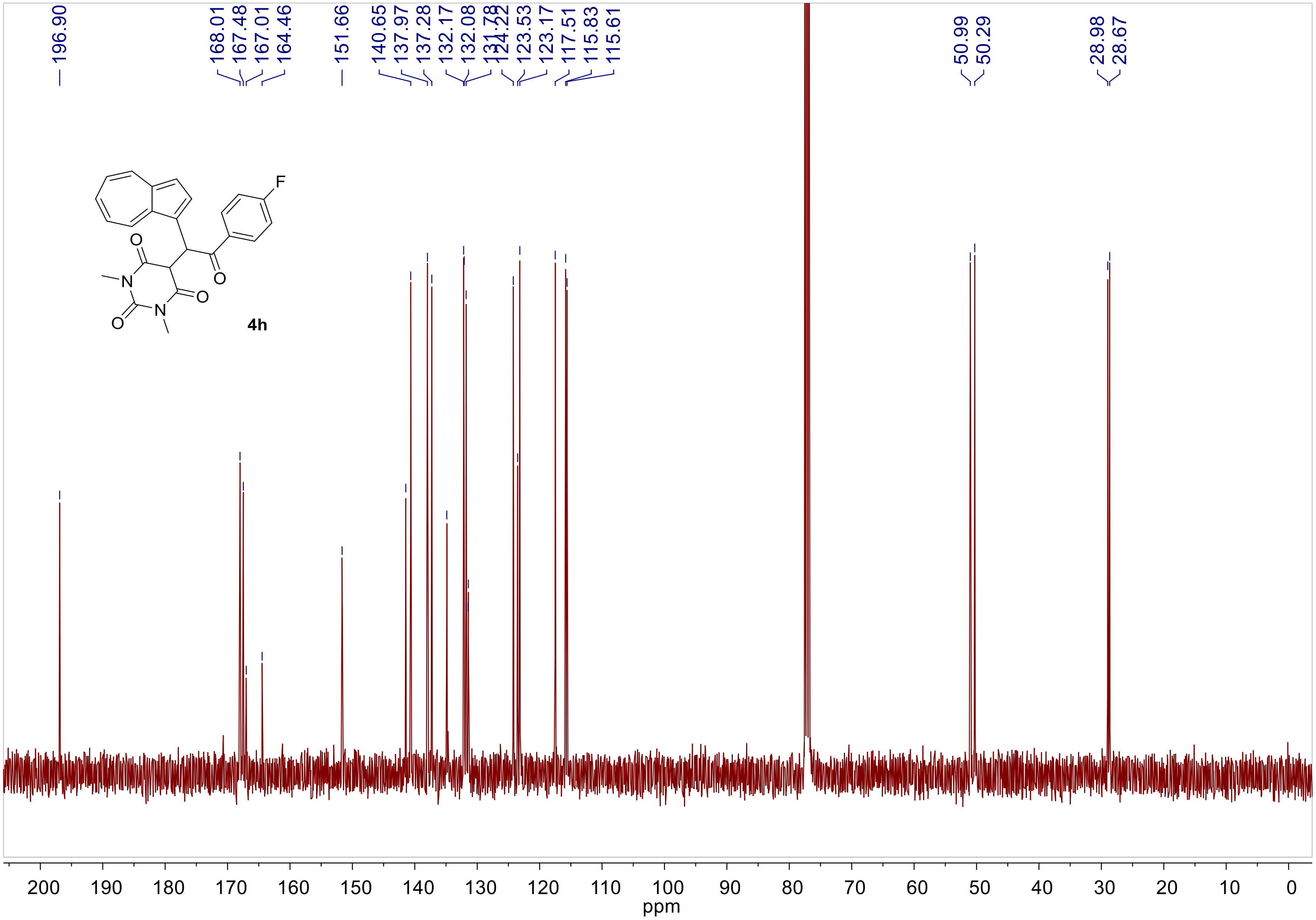
196.90
168.01
167.48
167.01
164.46
151.66
140.65
137.97
137.28
132.17
132.08
131.78
124.22
123.53
123.17
117.51
115.83
115.61
50.99
50.29
28.98
28.67
F
O
N
O
N
O
O
4h
200 190 180 170 160 150 140 130 120 110 100 90 80 70 60 50 40 30 20 10 0
ppm

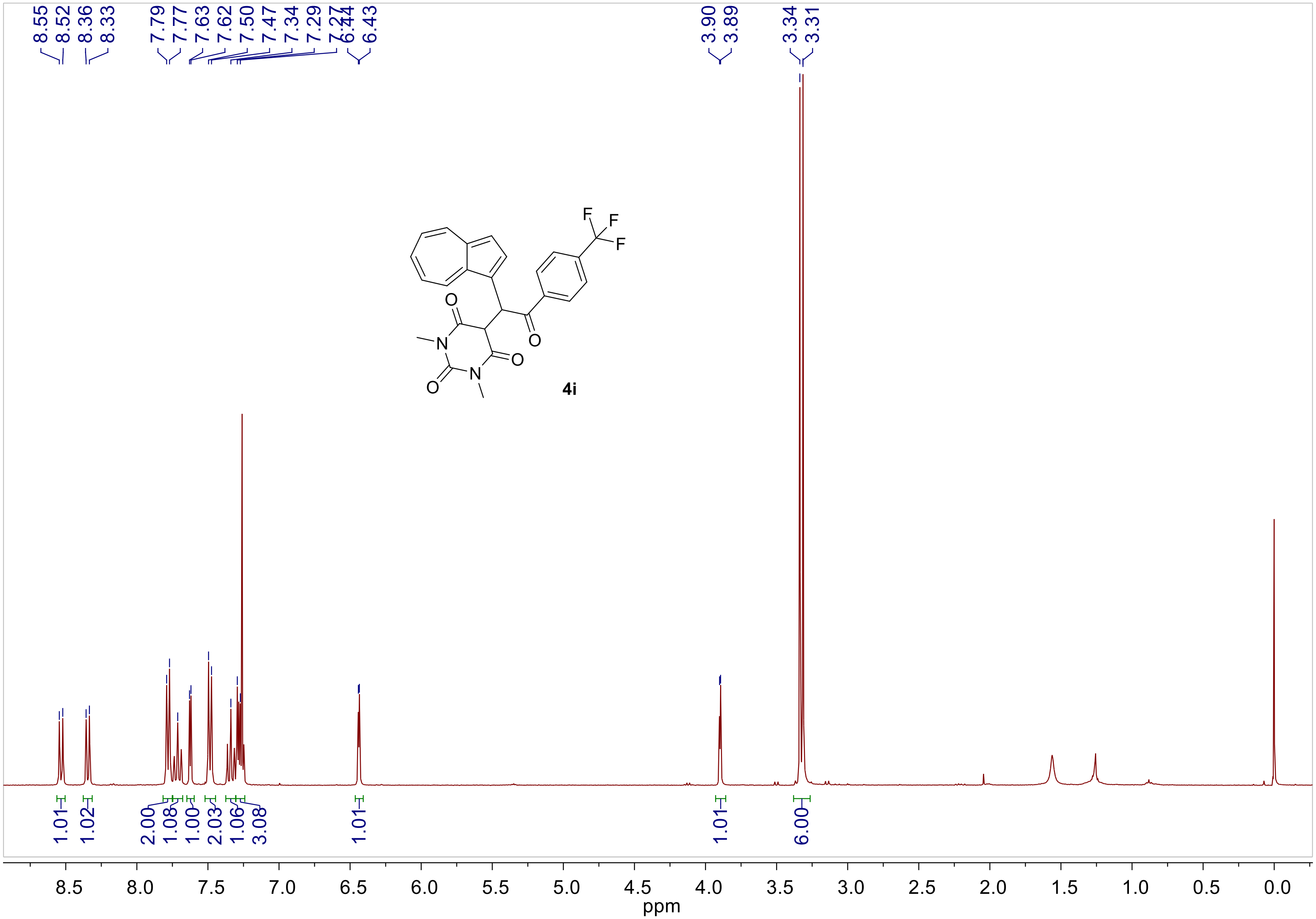
8.55
8.52
8.36
8.33
7.79
7.77
7.63
7.62
7.50
7.47
7.34
7.29
7.27
6.44
6.43
3.90
3.89
3.34
3.31
F
F
F
O
O
N
N
O
O
4i
1.01
1.02
2.00
1.08
1.00
2.03
1.06
3.08
1.01
1.01
6.00
8.5
8.0
7.5
7.0
6.5
6.0
5.5
5.0
4.5
4.0
3.5
3.0
2.5
2.0
1.5
1.0
0.5
0.0
ppm

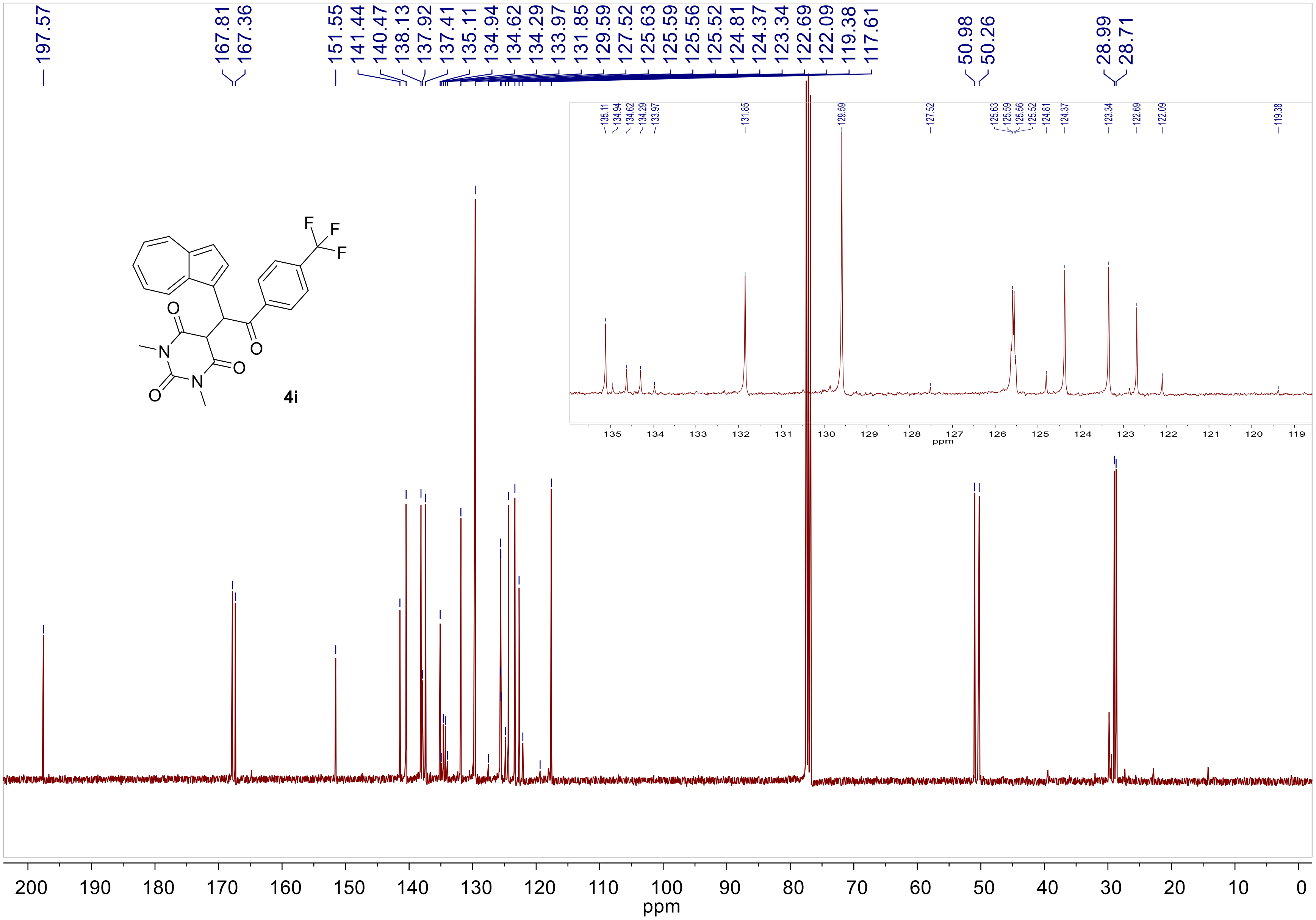
197.57
167.81
167.36
151.55
141.44
140.47
138.13
137.92
137.41
135.11
134.94
134.62
134.29
133.97
131.85
129.59
127.52
125.63
125.59
125.56
125.52
124.81
124.37
123.34
122.69
122.09
119.38
117.61
50.98
50.26
28.99
28.71
4i
F
F
F
O
N
N
ppm

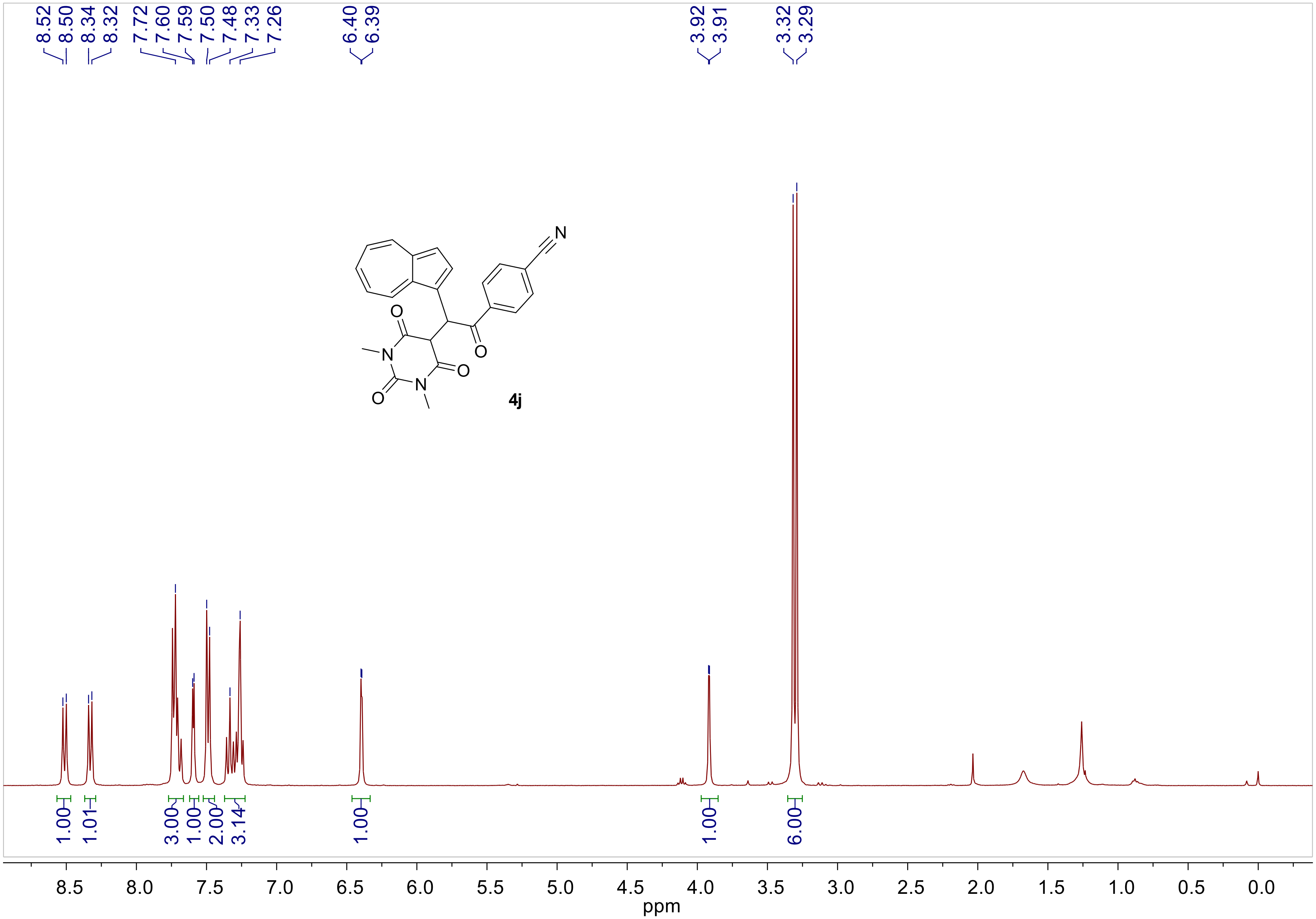
8.52
8.50
8.34
8.32
7.72
7.60
7.59
7.50
7.48
7.33
7.26
6.40
6.39
3.92
3.91
3.32
3.29
N
O
N
N
O
O
O
4j
1.00
1.01
3.00
1.00
2.00
3.14
1.00
1.00
6.00
8.5
8.0
7.5
7.0
6.5
6.0
5.5
5.0
4.5
4.0
3.5
3.0
2.5
2.0
1.5
1.0
0.5
0.0
ppm

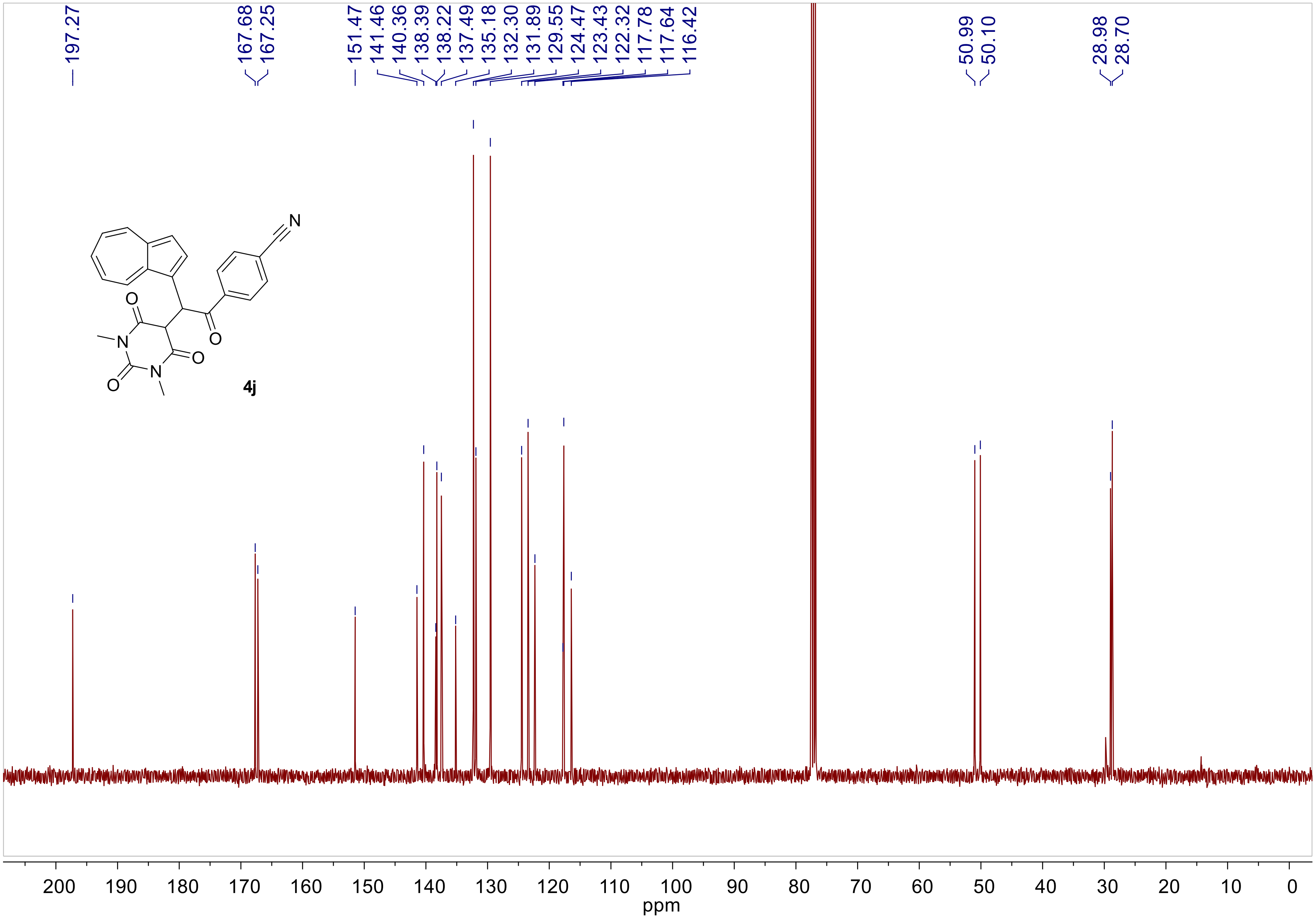
197.27
167.68
167.25
151.47
141.46
140.36
138.39
138.22
137.49
135.18
132.30
131.89
129.55
124.47
123.43
122.32
117.78
117.64
116.42
50.99
50.10
28.98
28.70
N
O
O
O
O
N
N
4j
200 190 180 170 160 150 140 130 120 110 100 90 80 70 60 50 40 30 20 10 0
ppm

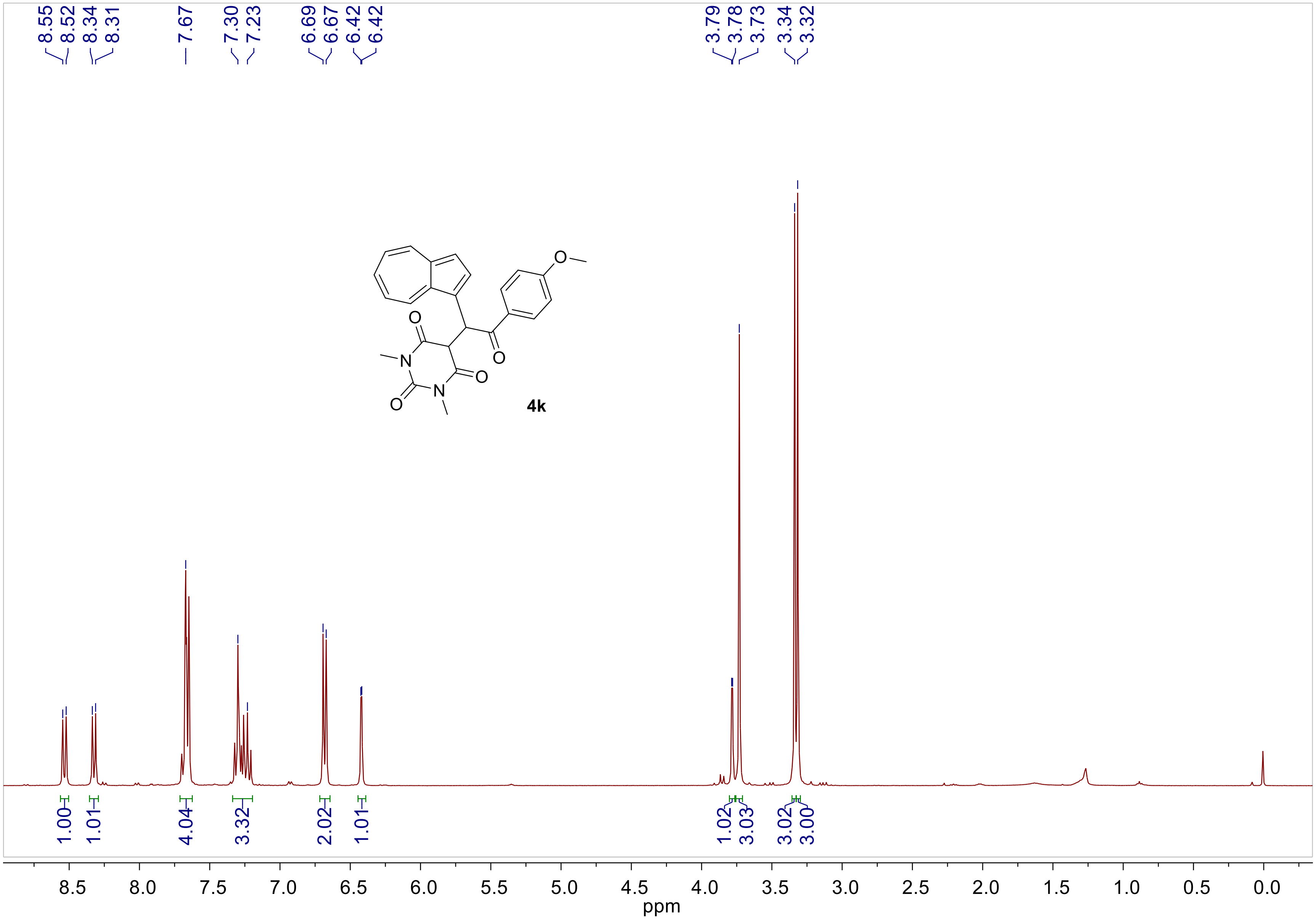
8.55
8.52
8.34
8.31
7.67
7.30
7.23
6.69
6.67
6.42
6.42
3.79
3.78
3.73
3.34
3.32
O
N
N
4k
1.00
1.01
4.04
3.32
2.02
1.01
1.02
3.03
3.02
3.00
8.5
8.0
7.5
7.0
6.5
6.0
5.5
5.0
4.5
4.0
3.5
3.0
2.5
2.0
1.5
1.0
0.5
0.0
ppm

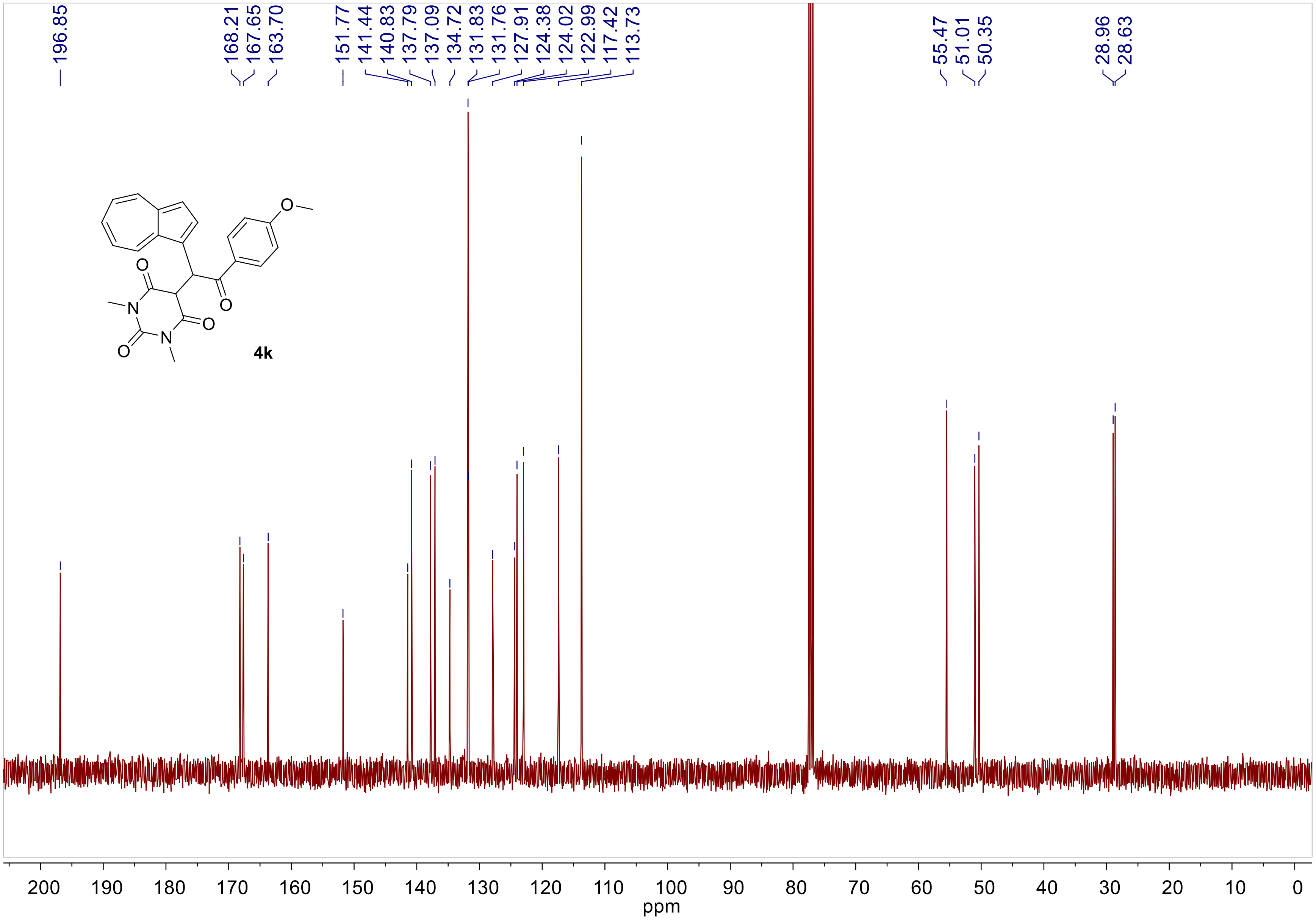
196.85
168.21
167.65
163.70
151.77
141.44
140.83
137.79
137.09
134.72
131.83
131.76
127.91
124.38
124.02
122.99
117.42
113.73
55.47
51.01
50.35
28.96
28.63
4k
200 190 180 170 160 150 140 130 120 110 100 90 80 70 60 50 40 30 20 10 0
ppm

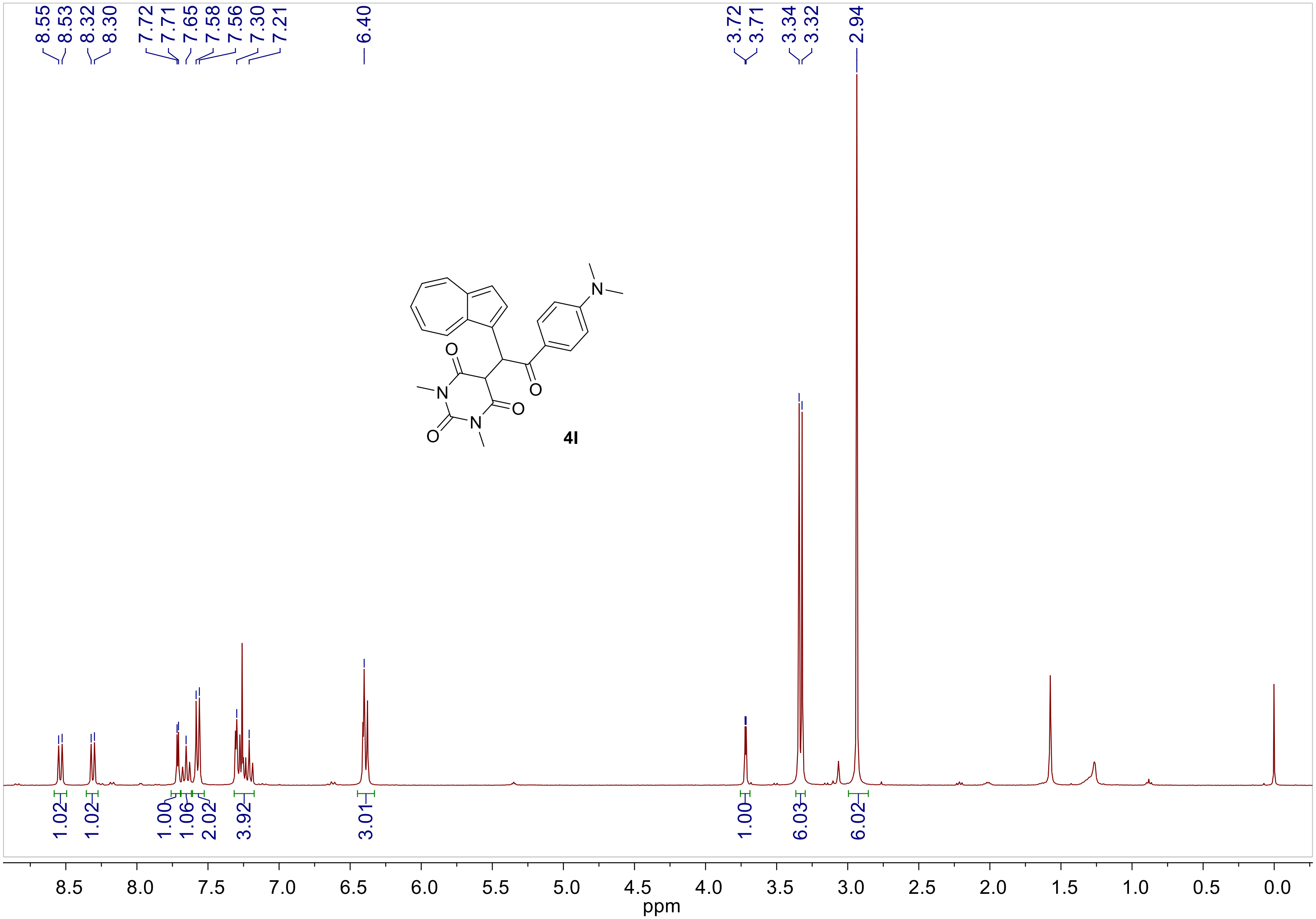
8.55
8.53
8.32
8.30
7.72
7.71
7.65
7.58
7.56
7.30
7.21
6.40
3.72
3.71
3.34
3.32
2.94
4l
1.02
1.02
1.00
1.06
2.02
3.92
3.01
1.00
6.03
6.02
8.5
8.0
7.5
7.0
6.5
6.0
5.5
5.0
4.5
4.0
3.5
3.0
2.5
2.0
1.5
1.0
0.5
0.0
ppm

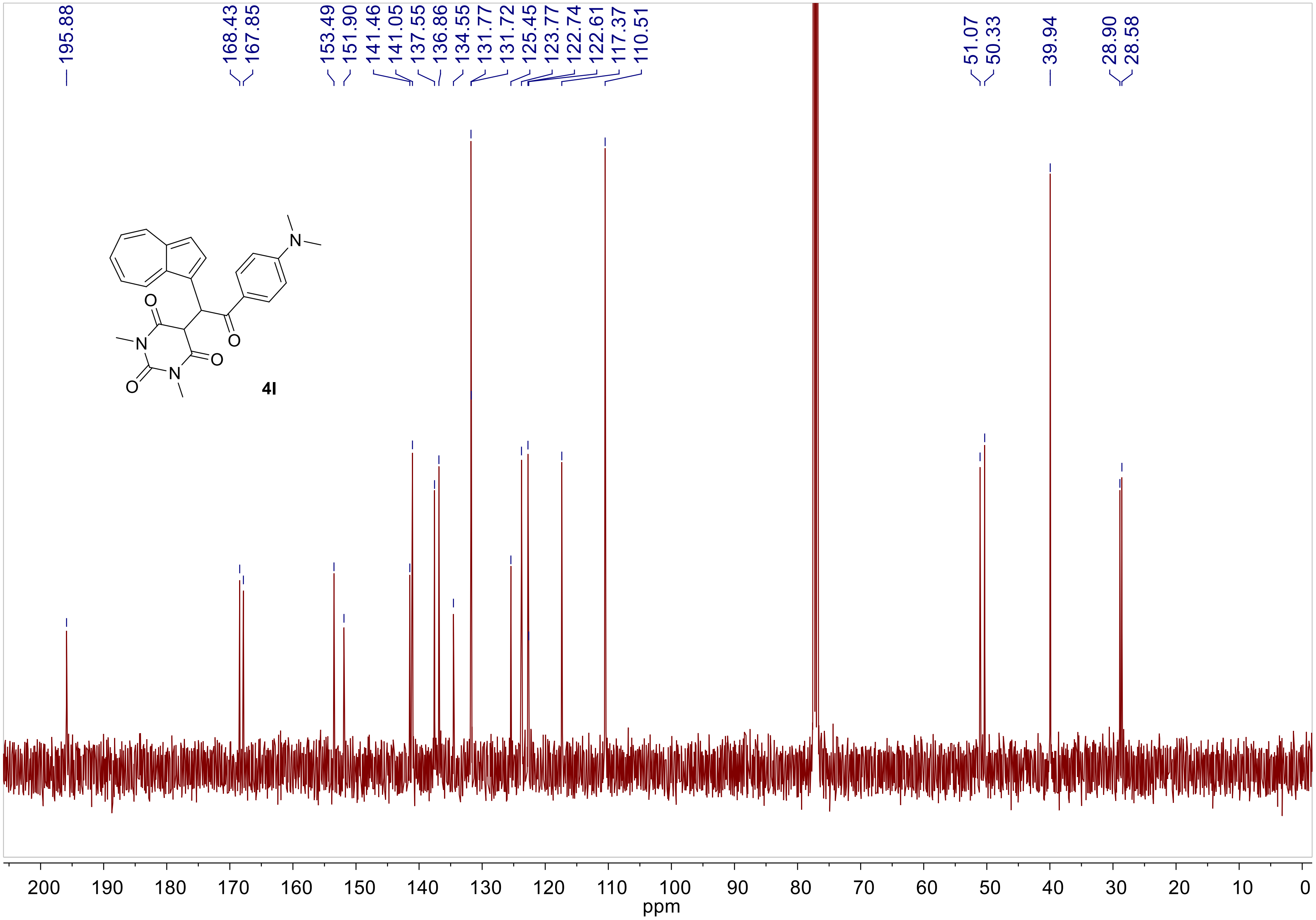
195.88
168.43
167.85
153.49
151.90
141.46
141.05
137.55
136.86
134.55
131.77
131.72
125.45
123.77
122.74
122.61
117.37
110.51
51.07
50.33
39.94
28.90
28.58
4l
200
190
180
170
160
150
140
130
120
110
100
90
80
70
60
50
40
30
20
10
0
ppm

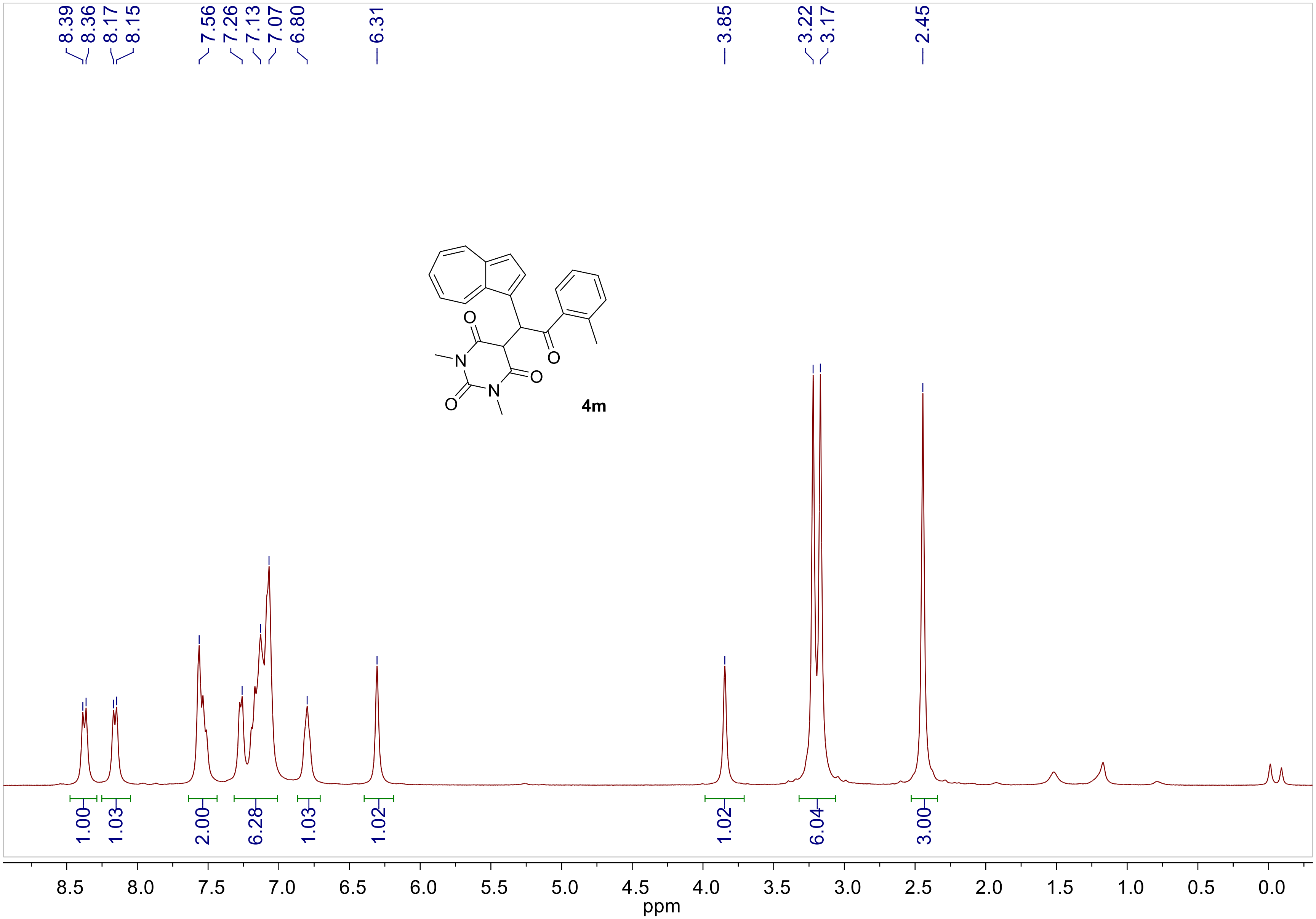
8.39
8.36
8.17
8.15
7.56
7.26
7.13
7.07
6.80
6.31
3.85
3.22
3.17
2.45
O
N
O
N
O
O
4m
1.00
1.03
2.00
6.28
1.03
1.02
1.02
6.04
3.00
8.5
8.0
7.5
7.0
6.5
6.0
5.5
5.0
4.5
4.0
3.5
3.0
2.5
2.0
1.5
1.0
0.5
0.0
ppm

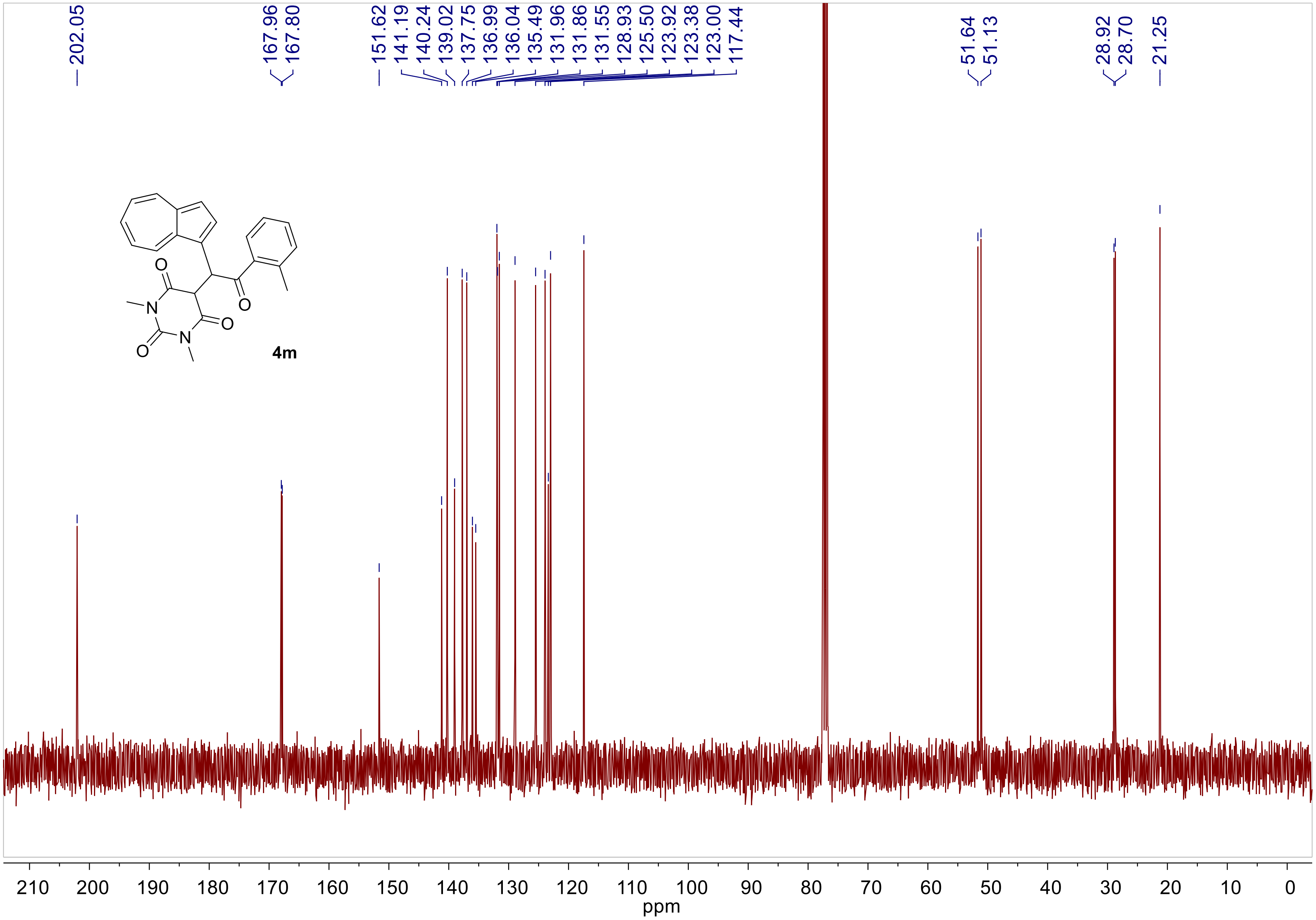
202.05
167.96
167.80
151.62
141.19
140.24
139.02
137.75
136.99
136.04
135.49
131.96
131.86
131.55
128.93
125.50
123.92
123.38
123.00
117.44
51.64
51.13
28.92
28.70
21.25
O
N
N
O
O
O
4m
210
200
190
180
170
160
150
140
130
120
110
100
90
80
70
60
50
40
30
20
10
0
ppm

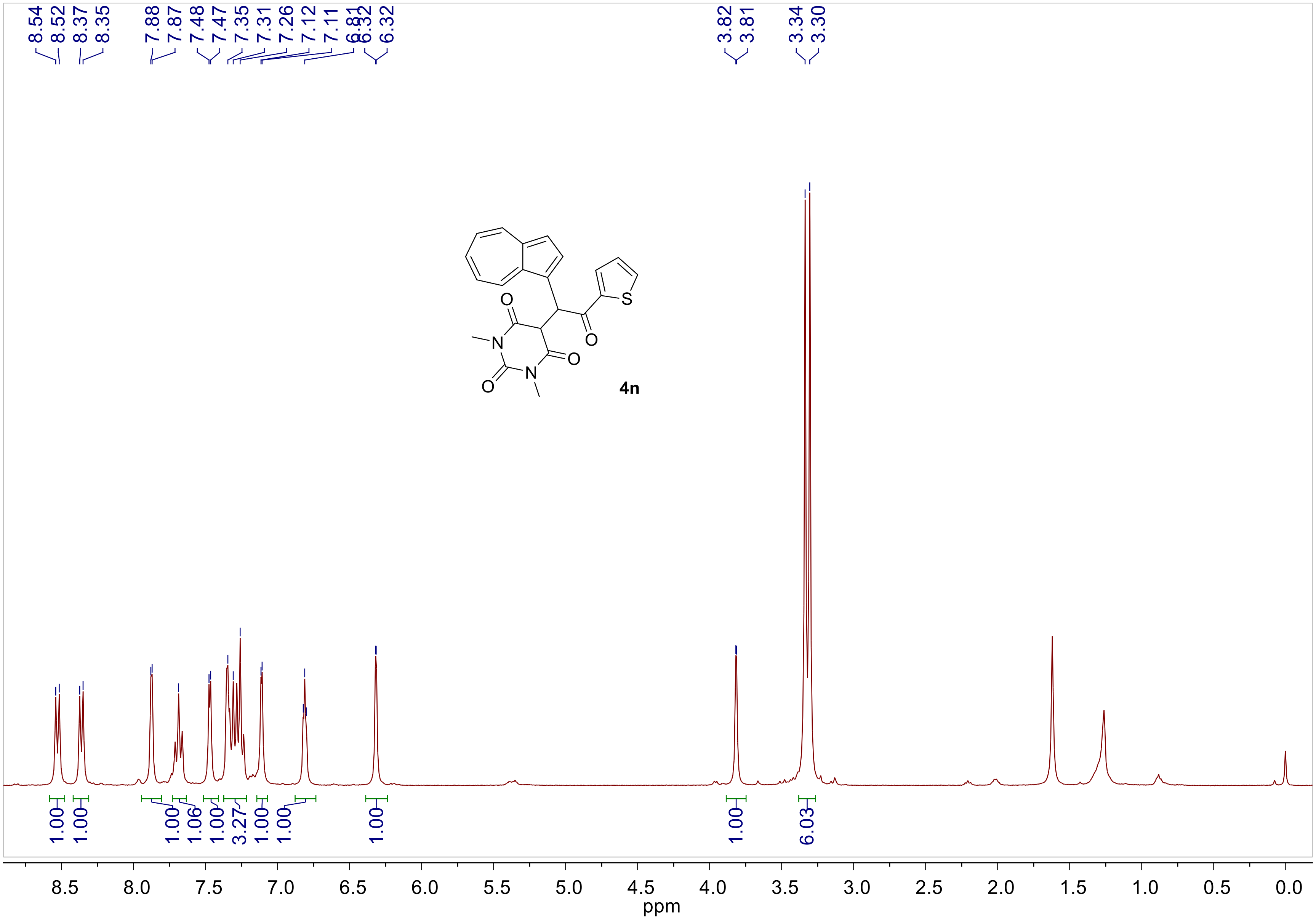

8.54
8.52
8.37
8.35
7.88
7.87
7.48
7.47
7.35
7.31
7.26
7.12
7.11
6.81
6.32
6.32
3.82
3.81
3.34
3.30
O
O
S
N
N
O
O
4n
1.00
1.00
1.00
1.06
1.00
3.27
1.00
1.00
1.00
1.00
6.03
8.5
8.0
7.5
7.0
6.5
6.0
5.5
5.0
4.5
4.0
3.5
3.0
2.5
2.0
1.5
1.0
0.5
0.0
ppm

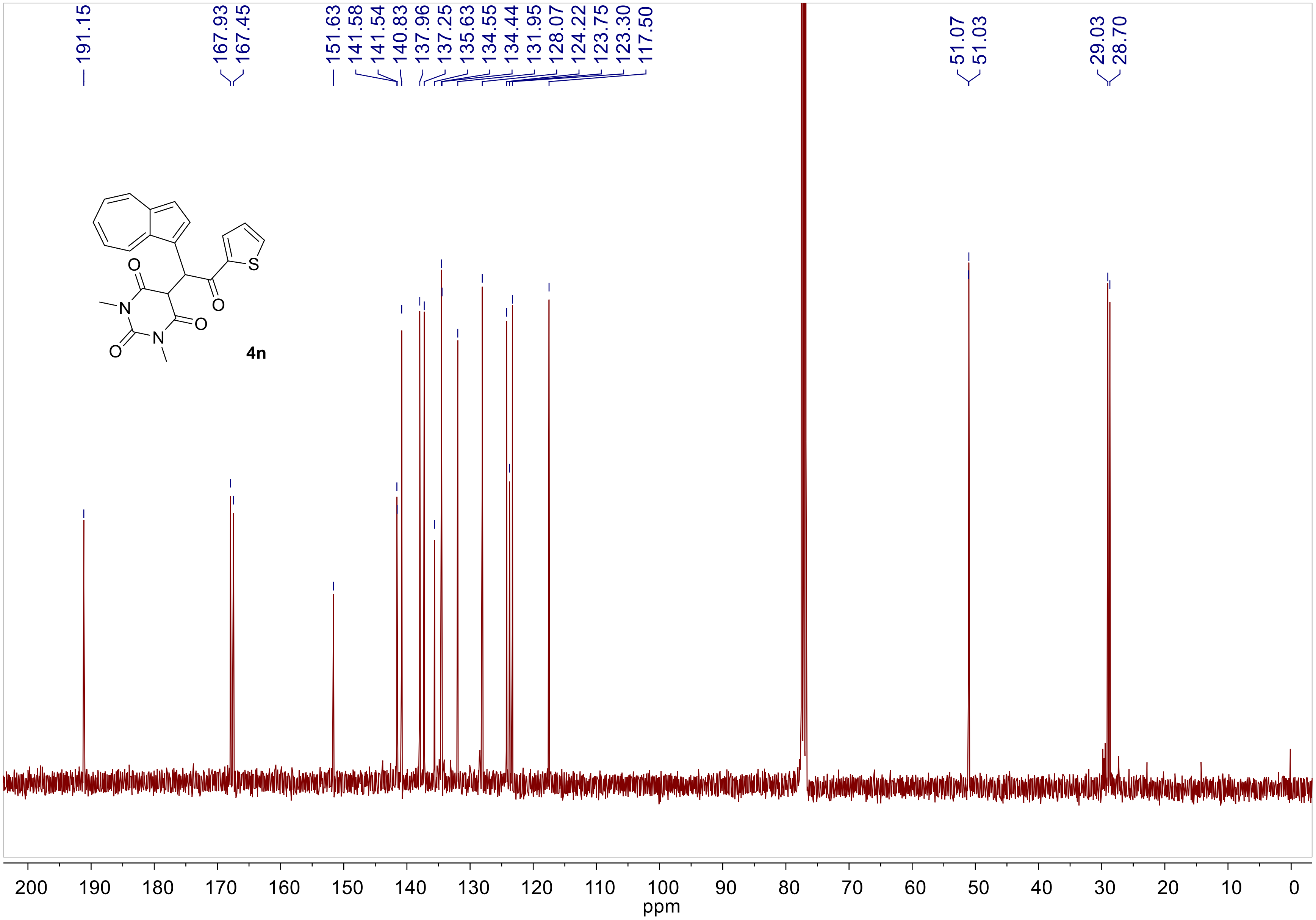
191.15
167.93
167.45
151.63
141.58
141.54
140.83
137.96
137.25
135.63
134.55
134.44
131.95
128.07
124.22
123.75
123.30
117.50
51.07
51.03
29.03
28.70
4n
200 190 180 170 160 150 140 130 120 110 100 90 80 70 60 50 40 30 20 10 0
ppm

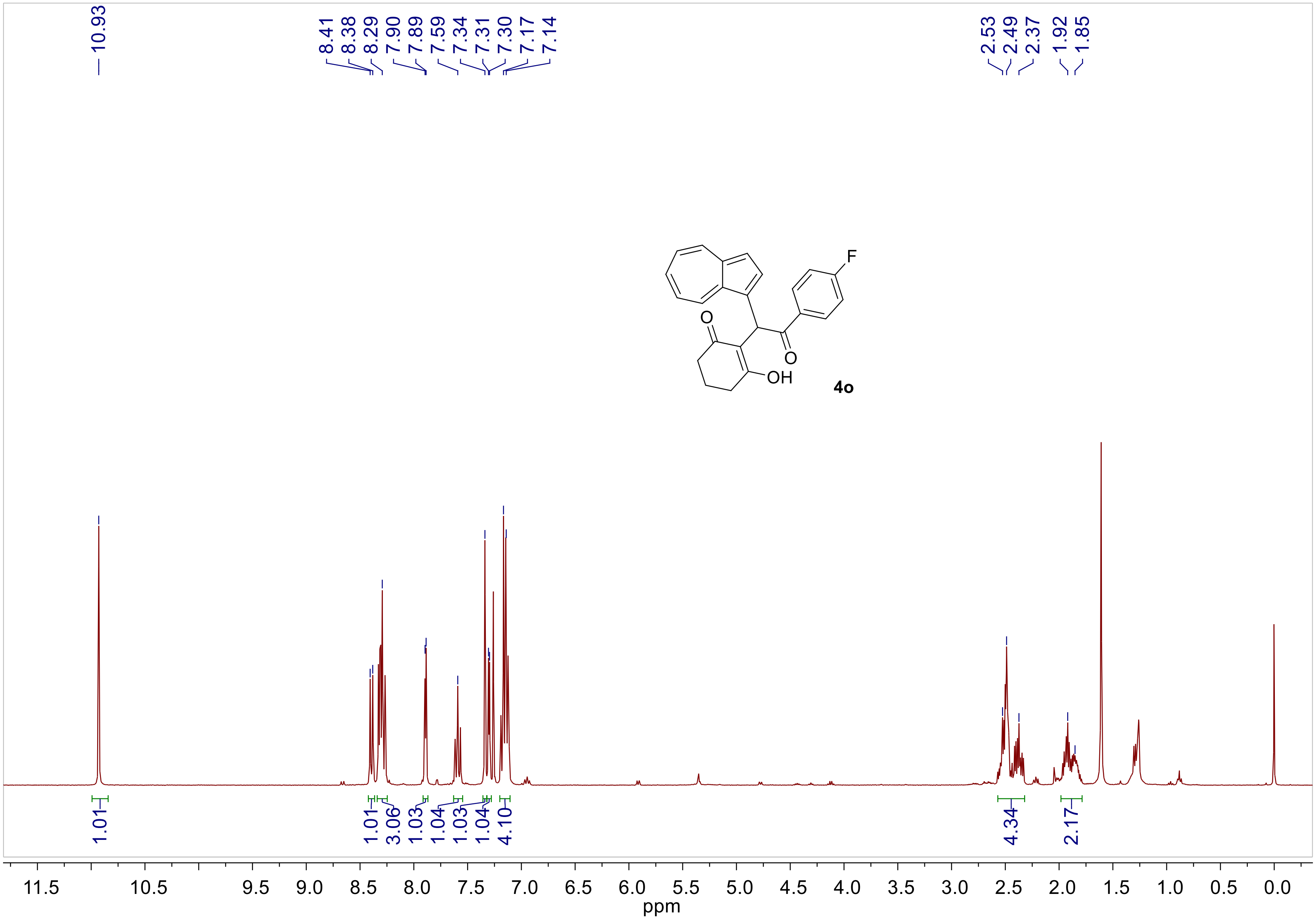
10.93
8.41
8.38
8.29
7.90
7.89
7.59
7.34
7.31
7.30
7.17
7.14
2.53
2.49
2.37
1.92
1.85
F
O
O
OH
4o
1.01
1.01
3.06
1.03
1.04
1.03
1.04
4.10
4.34
2.17
11.5
10.5
9.5
9.0
8.5
8.0
7.5
7.0
6.5
6.0
5.5
5.0
4.5
4.0
3.5
3.0
2.5
2.0
1.5
1.0
0.5
0.0
ppm

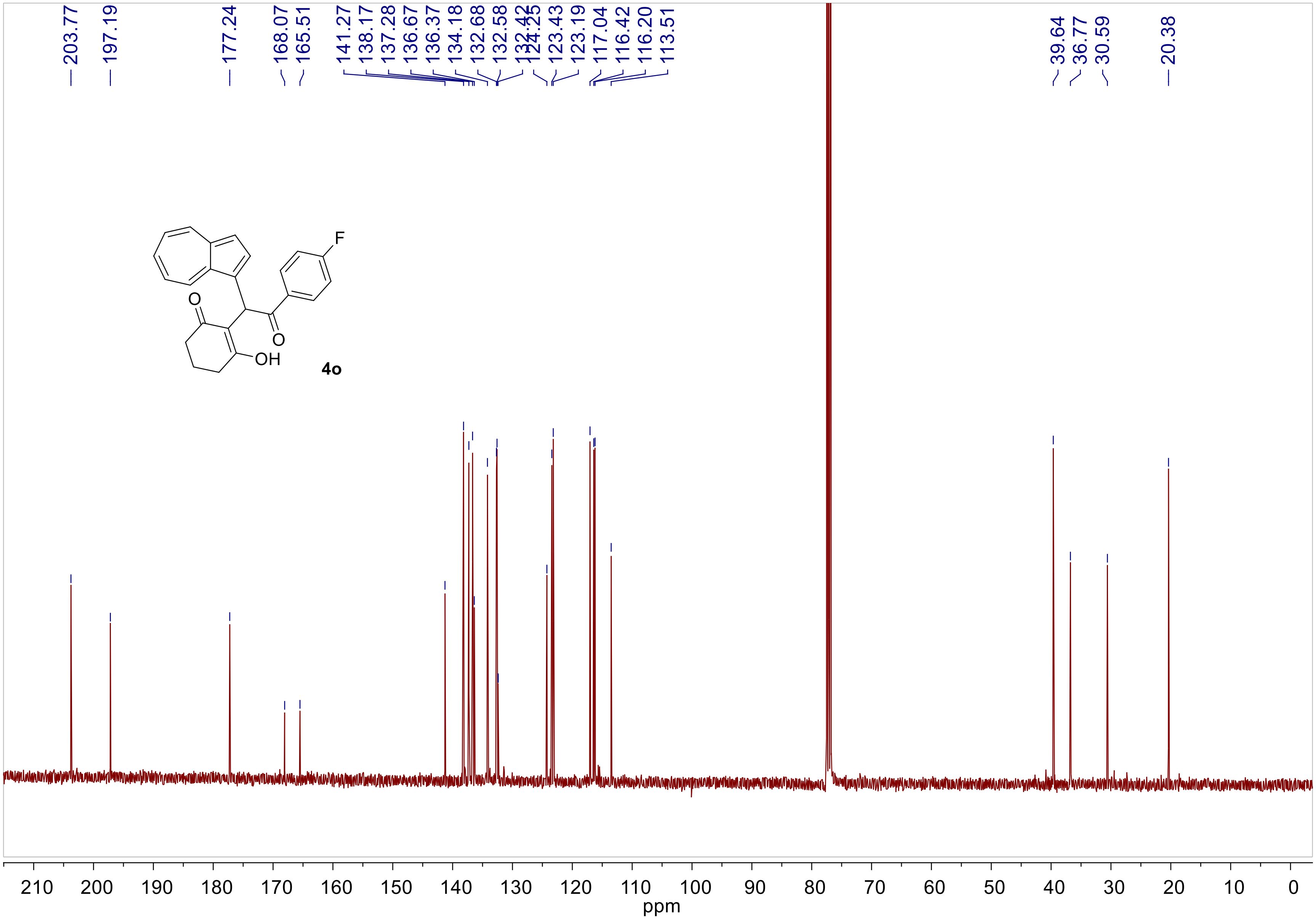
203.77
197.19
177.24
168.07
165.51
141.27
138.17
137.28
136.67
136.37
134.18
132.68
132.58
132.42
124.25
123.43
123.19
117.04
116.42
116.20
113.51
39.64
36.77
30.59
20.38
F
O
O
OH
4o
210 200 190 180 170 160 150 140 130 120 110 100 90 80 70 60 50 40 30 20 10 0
ppm

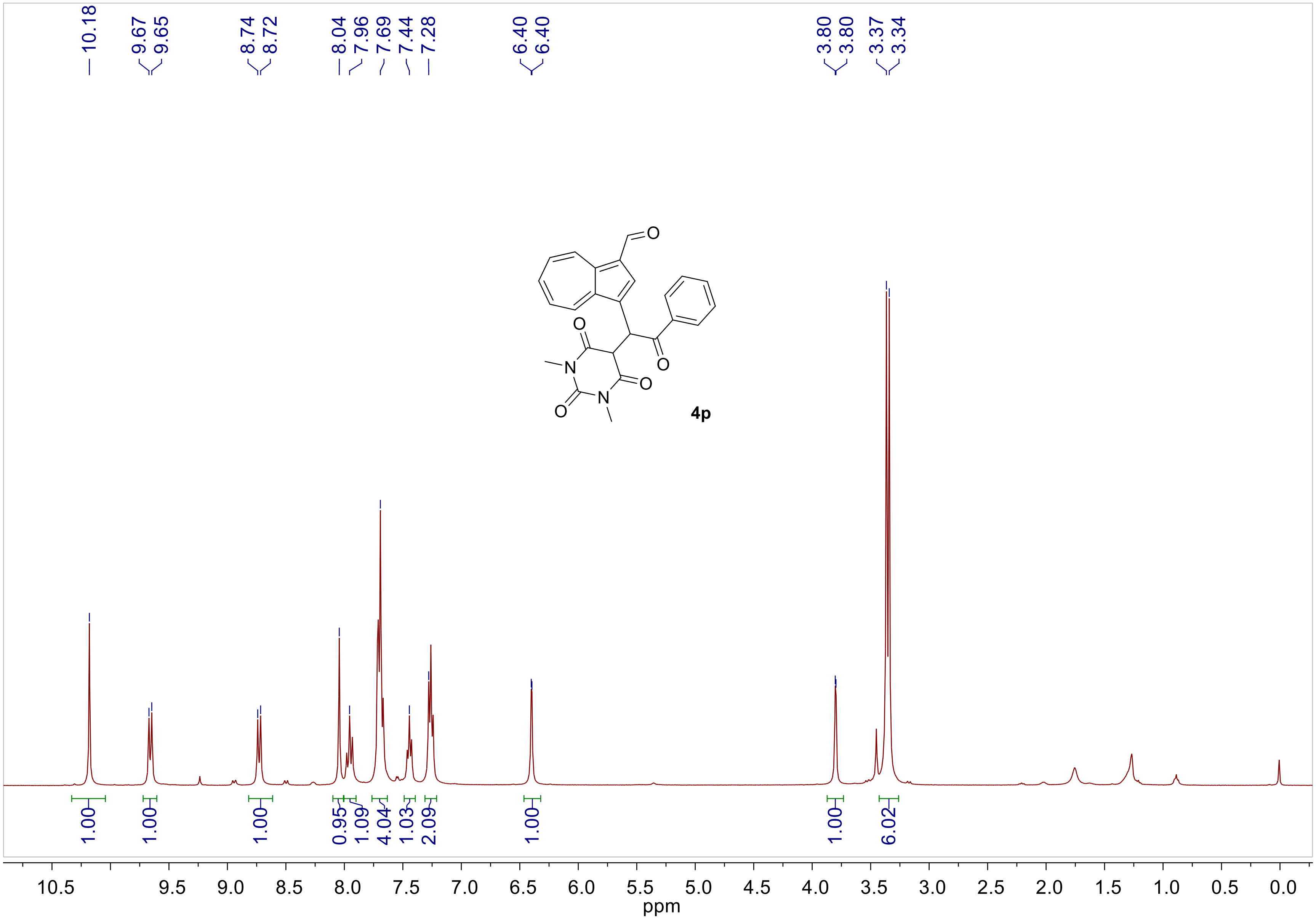
10.18
9.67
9.65
8.74
8.72
8.04
7.96
7.69
7.44
7.28
6.40
6.40
3.80
3.80
3.37
3.34
4p
1.00
1.00
1.00
0.95
1.09
4.04
1.03
2.09
1.00
1.00
6.02
10.5
9.5
9.0
8.5
8.0
7.5
7.0
6.5
6.0
5.5
5.0
4.5
4.0
3.5
3.0
2.5
2.0
1.5
1.0
0.5
0.0
ppm

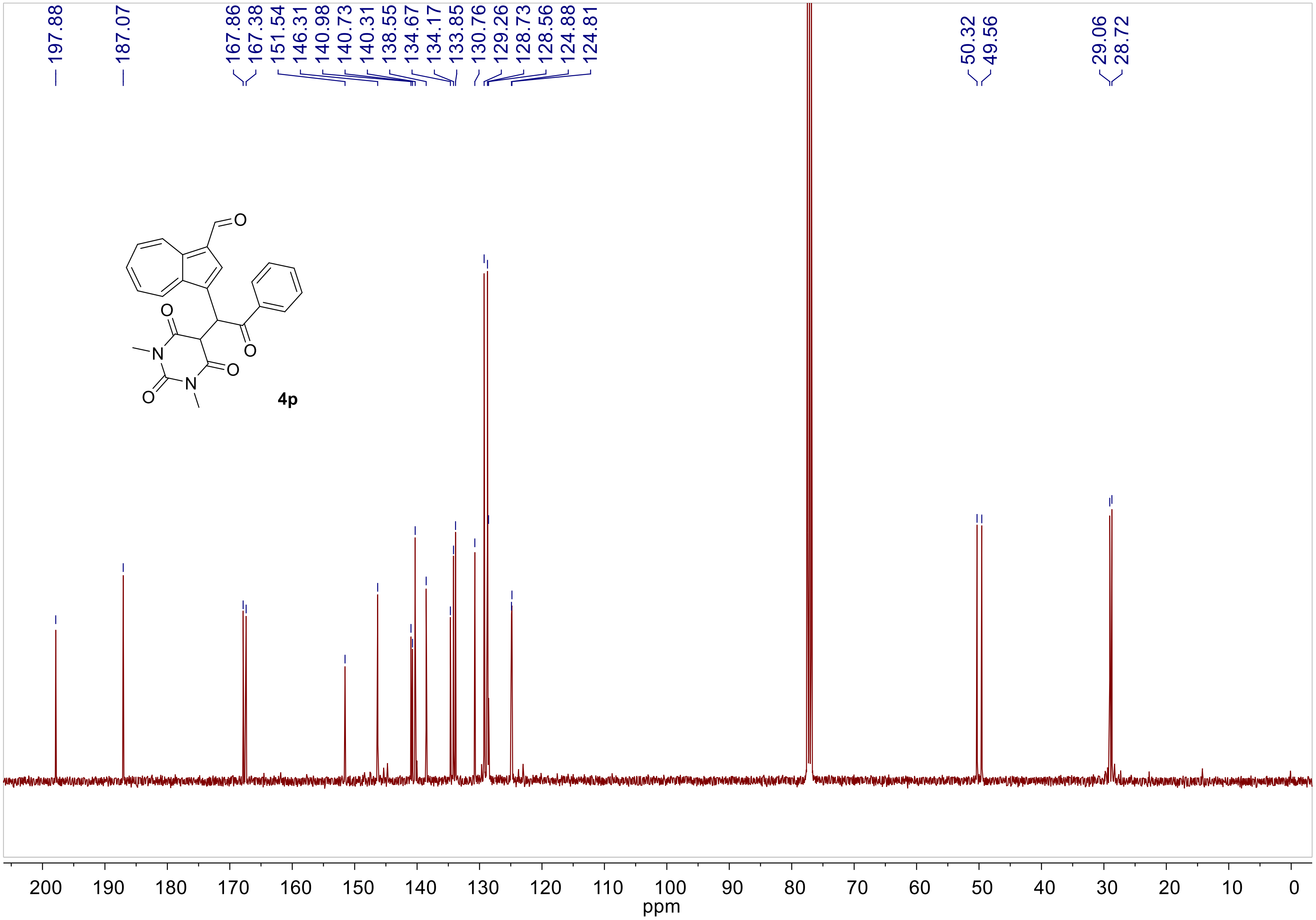
197.88
187.07
167.86
167.38
151.54
146.31
140.98
140.73
140.31
138.55
134.67
134.17
133.85
130.76
129.26
128.73
128.56
124.88
124.81
50.32
49.56
29.06
28.72
O
O
N
N
O
O
O
4p
200 190 180 170 160 150 140 130 120 110 100 90 80 70 60 50 40 30 20 10 0
ppm

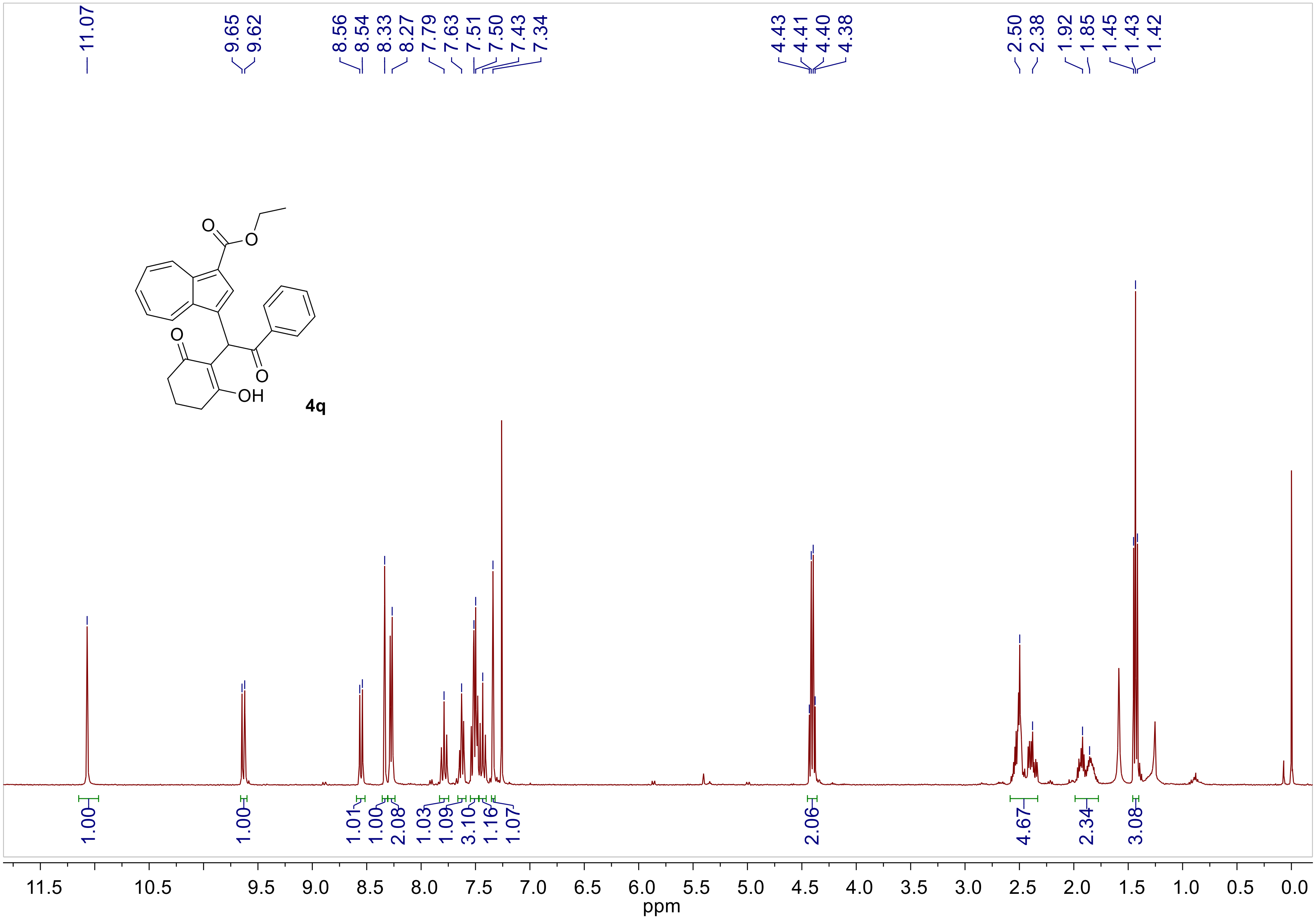
11.07
9.65
9.62
8.56
8.54
8.33
8.27
7.79
7.63
7.51
7.50
7.43
7.34
4.43
4.41
4.40
4.38
2.50
2.38
1.92
1.85
1.45
1.43
1.42
O
O
O
O
OH
4q
1.00
1.00
1.01
1.00
2.08
1.03
1.09
3.10
1.16
1.07
2.06
4.67
2.34
3.08
11.5
10.5
9.5
9.0
8.5
8.0
7.5
7.0
6.5
6.0
5.5
5.0
4.5
4.0
3.5
3.0
2.5
2.0
1.5
1.0
0.5
0.0
ppm

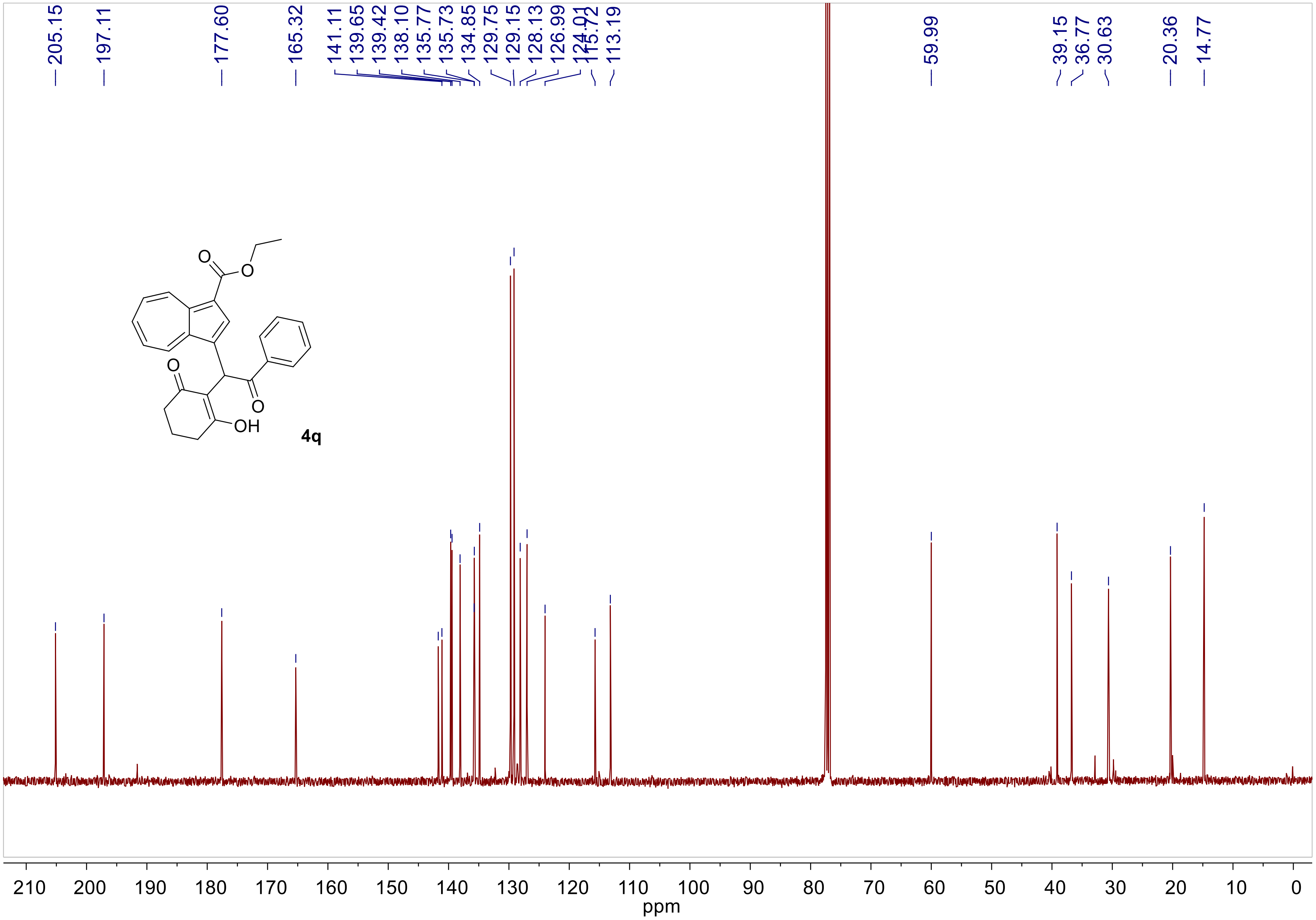
205.15
197.11
177.60
165.32
141.11
139.65
139.42
138.10
135.77
135.73
134.85
129.75
129.15
128.13
126.99
124.01
115.72
113.19
59.99
39.15
36.77
30.63
20.36
14.77
O
O
O
OH
O
4q
210 200 190 180 170 160 150 140 130 120 110 100 90 80 70 60 50 40 30 20 10 0
ppm

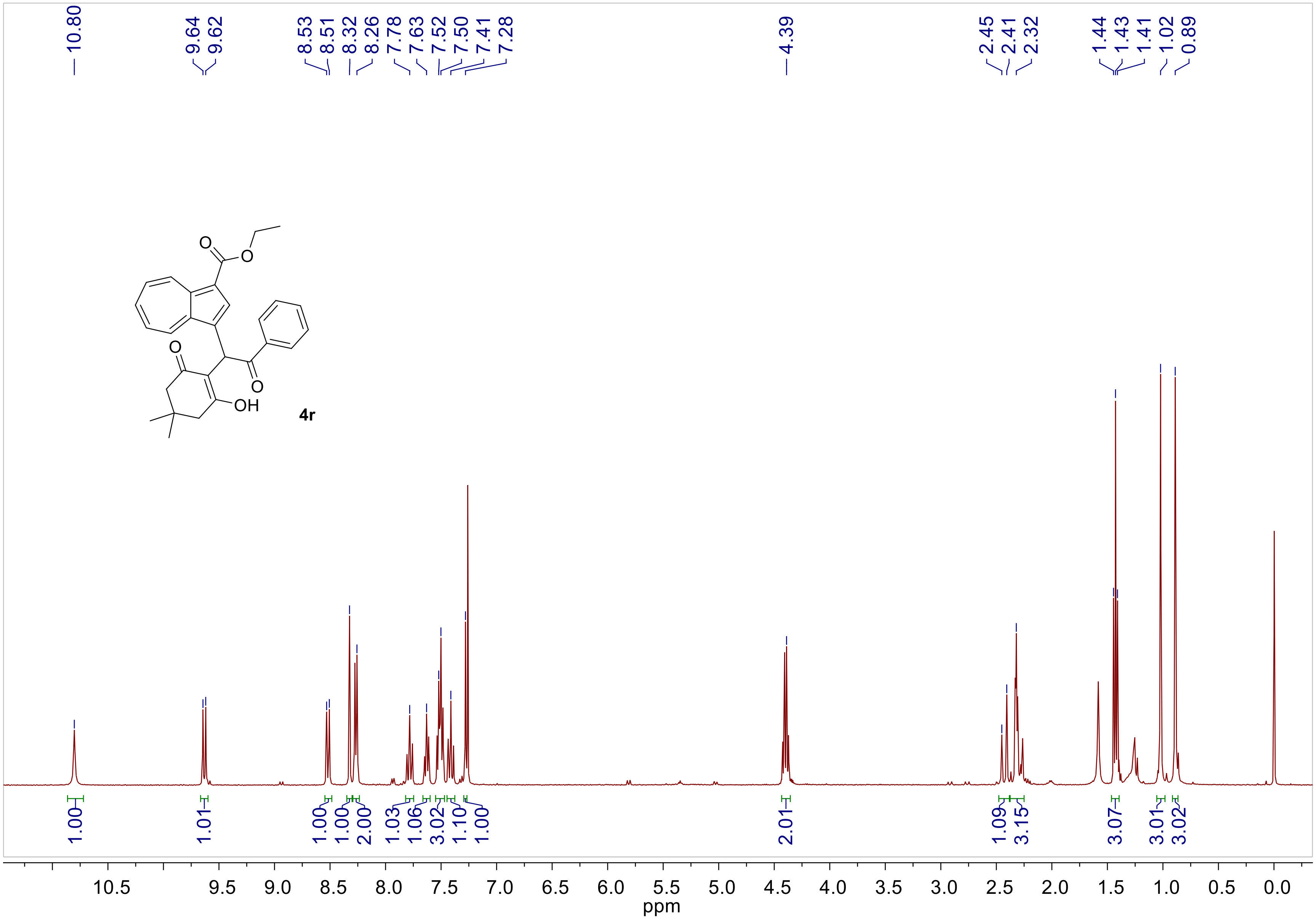
10.80
9.64
9.62
8.53
8.51
8.32
8.26
7.78
7.63
7.52
7.50
7.41
7.28
4.39
2.45
2.41
2.32
1.44
1.43
1.41
1.02
0.89
O
O
O
O
OH
4r
1.00
1.01
1.00
1.00
2.00
1.03
1.06
3.02
1.10
1.00
2.01
1.09
3.15
3.07
3.01
3.02
10.5
9.5
9.0
8.5
8.0
7.5
7.0
6.5
6.0
5.5
5.0
4.5
4.0
3.5
3.0
2.5
2.0
1.5
1.0
0.5
0.0
ppm

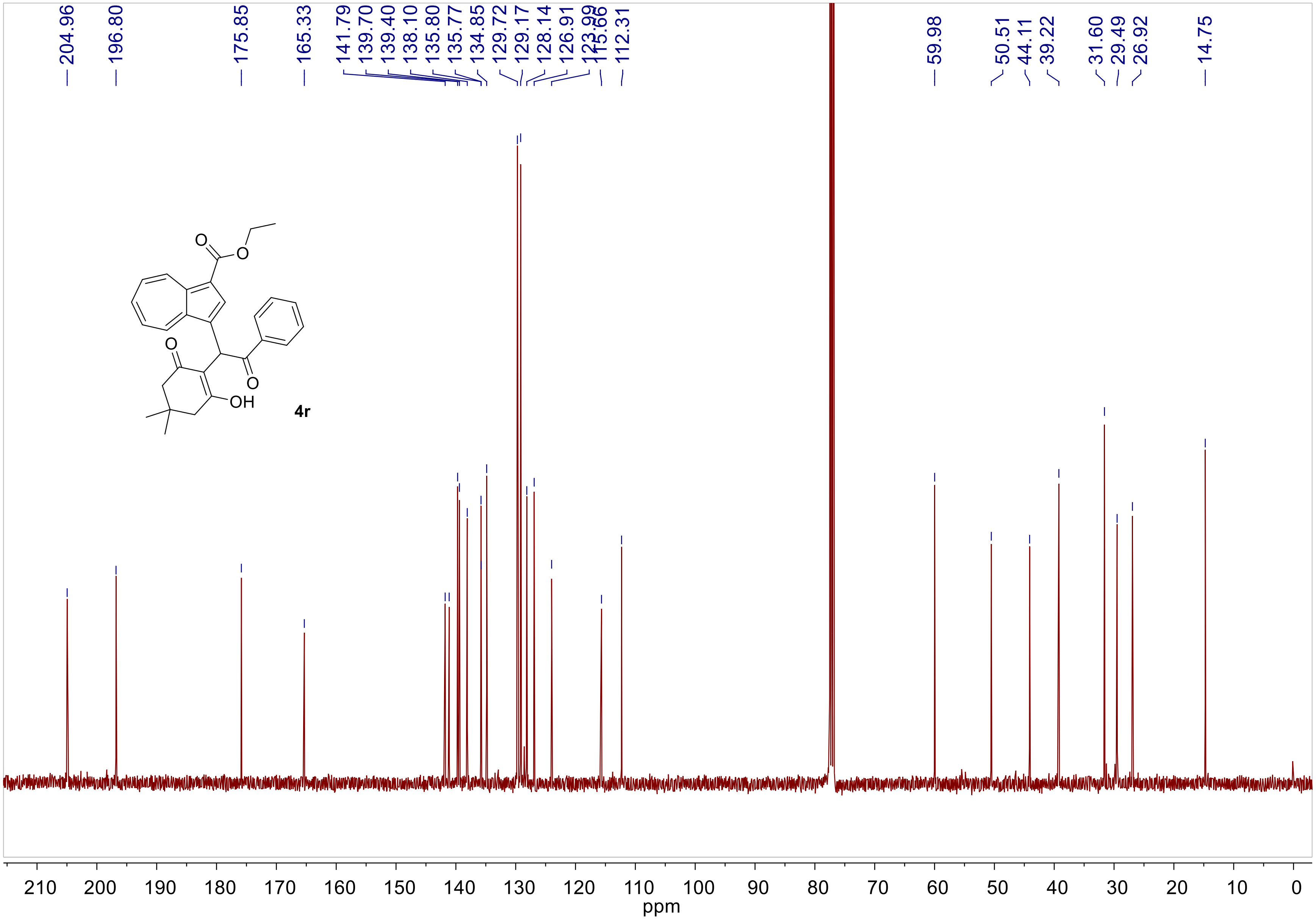
204.96
196.80
175.85
165.33
141.79
139.70
139.40
138.10
135.80
135.77
134.85
129.72
129.17
128.14
126.91
123.99
115.66
112.31
59.98
50.51
44.11
39.22
31.60
29.49
26.92
14.75
O
O
O
O
OH
4r
210
200
190
180
170
160
150
140
130
120
110
100
90
80
70
60
50
40
30
20
10
0
ppm

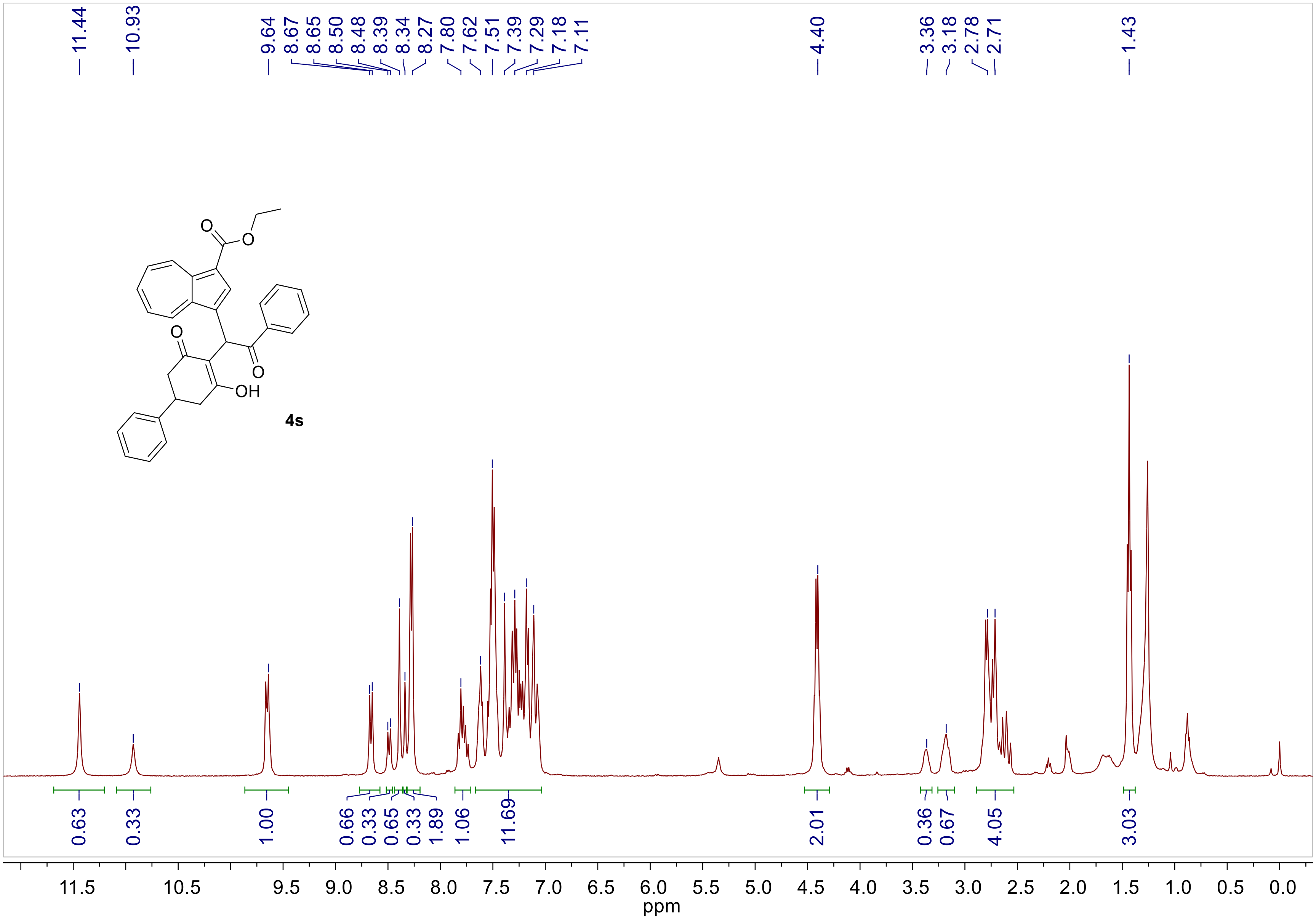
11.44
10.93
9.64
8.67
8.65
8.50
8.48
8.39
8.34
8.27
7.80
7.62
7.51
7.39
7.29
7.18
7.11
4.40
3.36
3.18
2.78
2.71
1.43
O
O
O
O
OH
4s
0.63
0.33
1.00
0.66
0.33
0.65
0.33
1.89
1.06
11.69
2.01
0.36
0.67
4.05
3.03
11.5
10.5
9.5
9.0
8.5
8.0
7.5
7.0
6.5
6.0
5.5
5.0
4.5
4.0
3.5
3.0
2.5
2.0
1.5
1.0
0.5
0.0
ppm

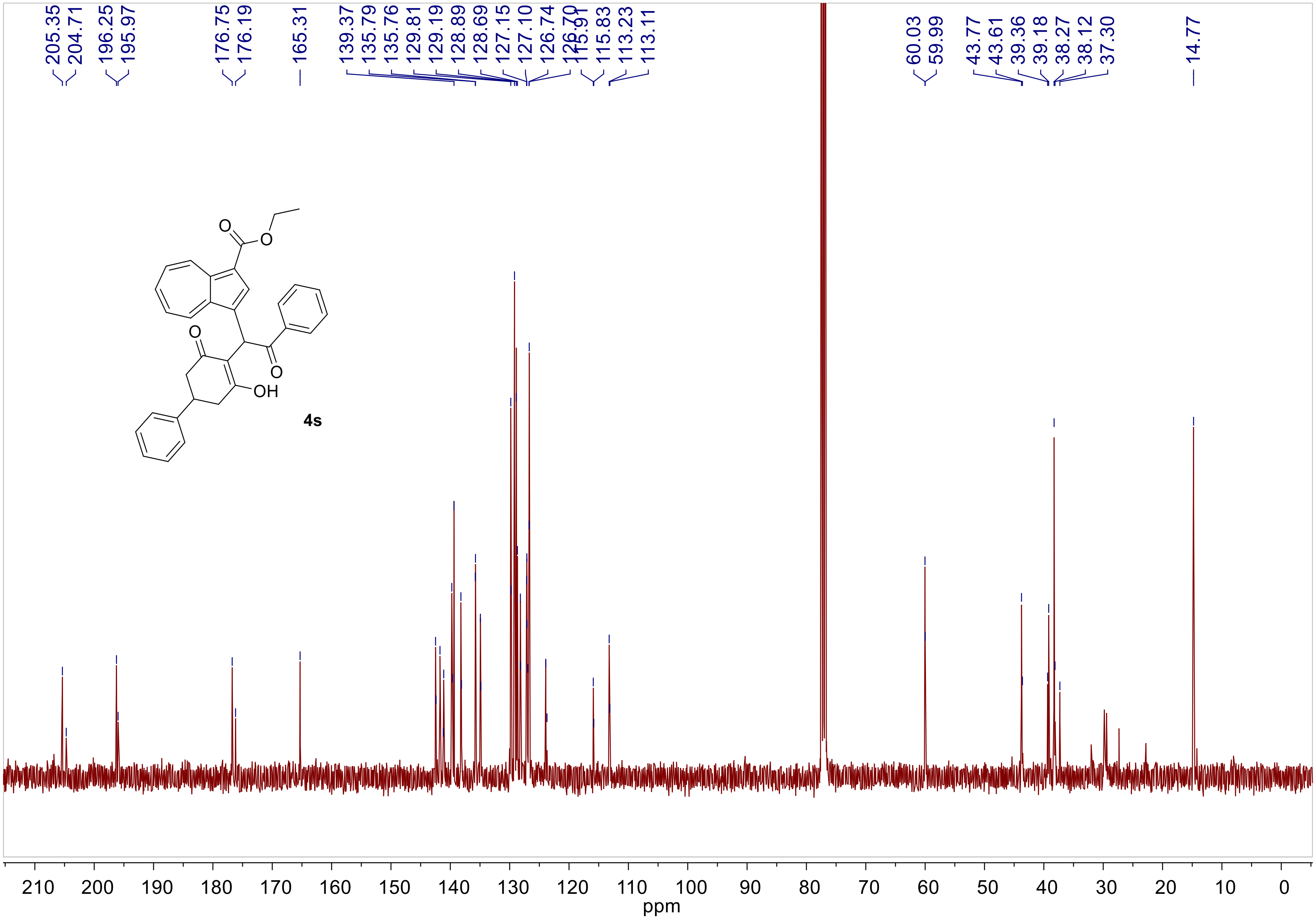

205.35
204.71
196.25
195.97
176.75
176.19
165.31
139.37
135.79
135.76
129.81
129.19
128.89
128.69
127.15
127.10
126.74
126.70
115.91
115.83
113.23
113.11
60.03
59.99
43.77
43.61
39.36
39.18
38.27
38.12
37.30
14.77
O
O
O
O
OH
4s
210
200
190
180
170
160
150
140
130
120
110
100
90
80
70
60
50
40
30
20
10
0
ppm

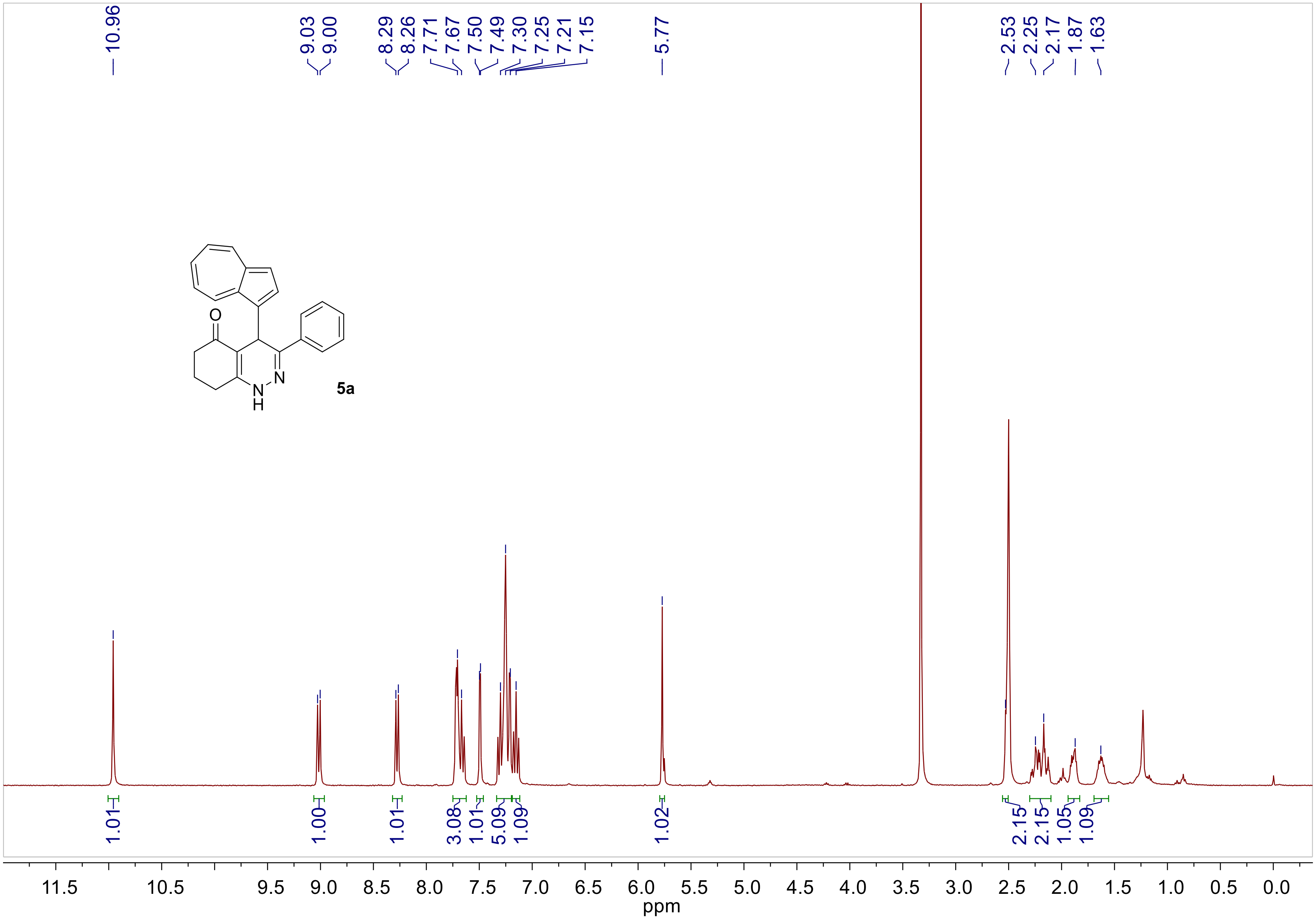
10.96
9.03
9.00
8.29
8.26
7.71
7.67
7.50
7.49
7.30
7.25
7.21
7.15
5.77
2.53
2.25
2.17
1.87
1.63
O
N
N
H
5a
1.01
1.00
1.01
3.08
1.01
5.09
1.09
1.02
2.15
2.15
1.05
1.09
11.5
10.5
9.5
9.0
8.5
8.0
7.5
7.0
6.5
6.0
5.5
5.0
4.5
4.0
3.5
3.0
2.5
2.0
1.5
1.0
0.5
0.0
ppm

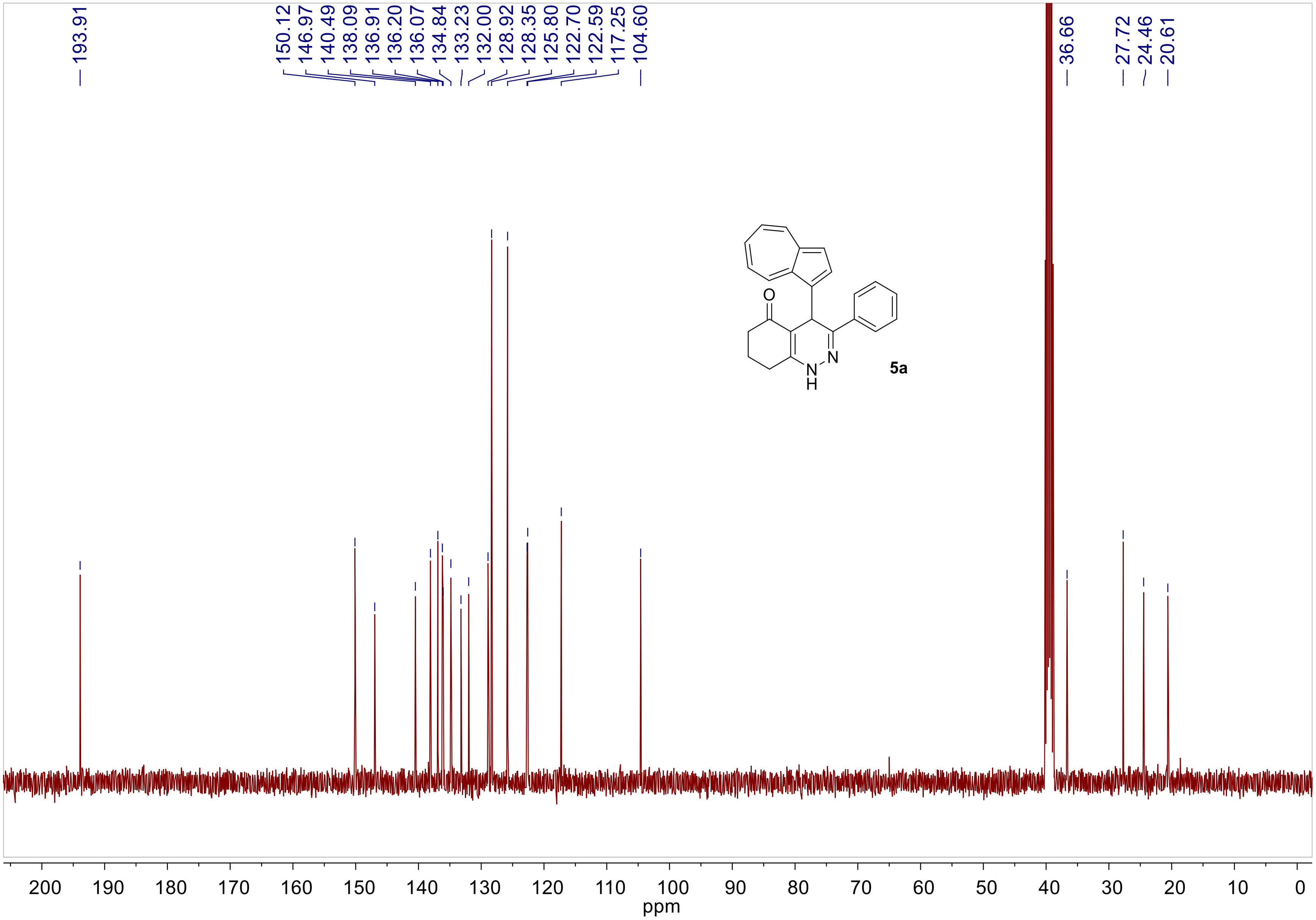
193.91
150.12
146.97
140.49
138.09
136.91
136.20
136.07
134.84
133.23
132.00
128.92
128.35
125.80
122.70
122.59
117.25
104.60
36.66
27.72
24.46
20.61
O
N
N
H
5a
200 190 180 170 160 150 140 130 120 110 100 90 80 70 60 50 40 30 20 10 0
ppm

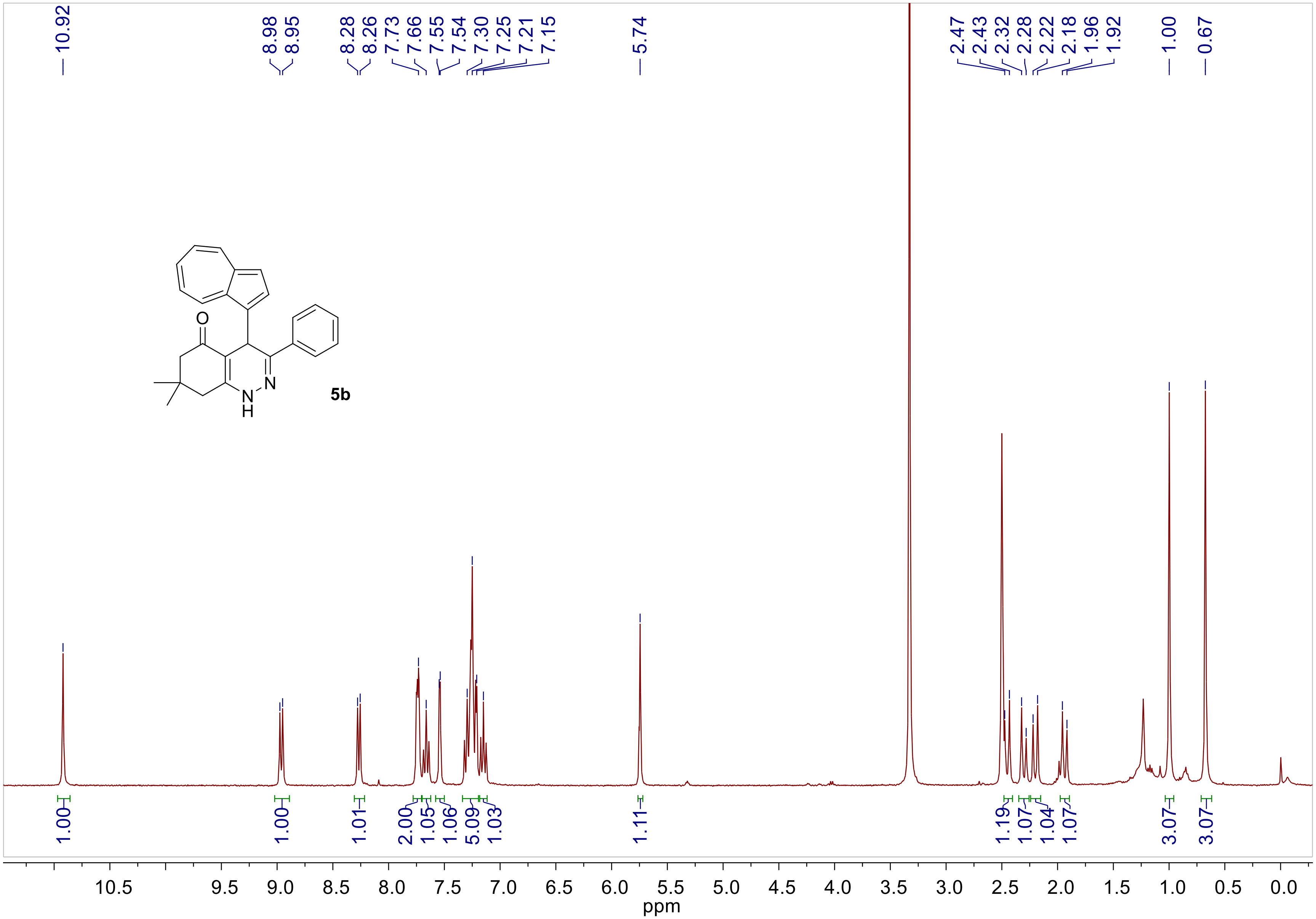

10.92
8.98
8.95
8.28
8.26
7.73
7.66
7.55
7.54
7.30
7.25
7.21
7.15
5.74
2.47
2.43
2.32
2.28
2.22
2.18
1.96
1.92
1.00
0.67
O
N
N
H
5b
1.00
1.00
1.01
2.00
1.05
1.06
5.09
1.03
1.11
1.19
1.07
1.04
1.07
3.07
3.07
10.5
9.5
9.0
8.5
8.0
7.5
7.0
6.5
6.0
5.5
5.0
4.5
4.0
3.5
3.0
2.5
2.0
1.5
1.0
0.5
0.0
ppm

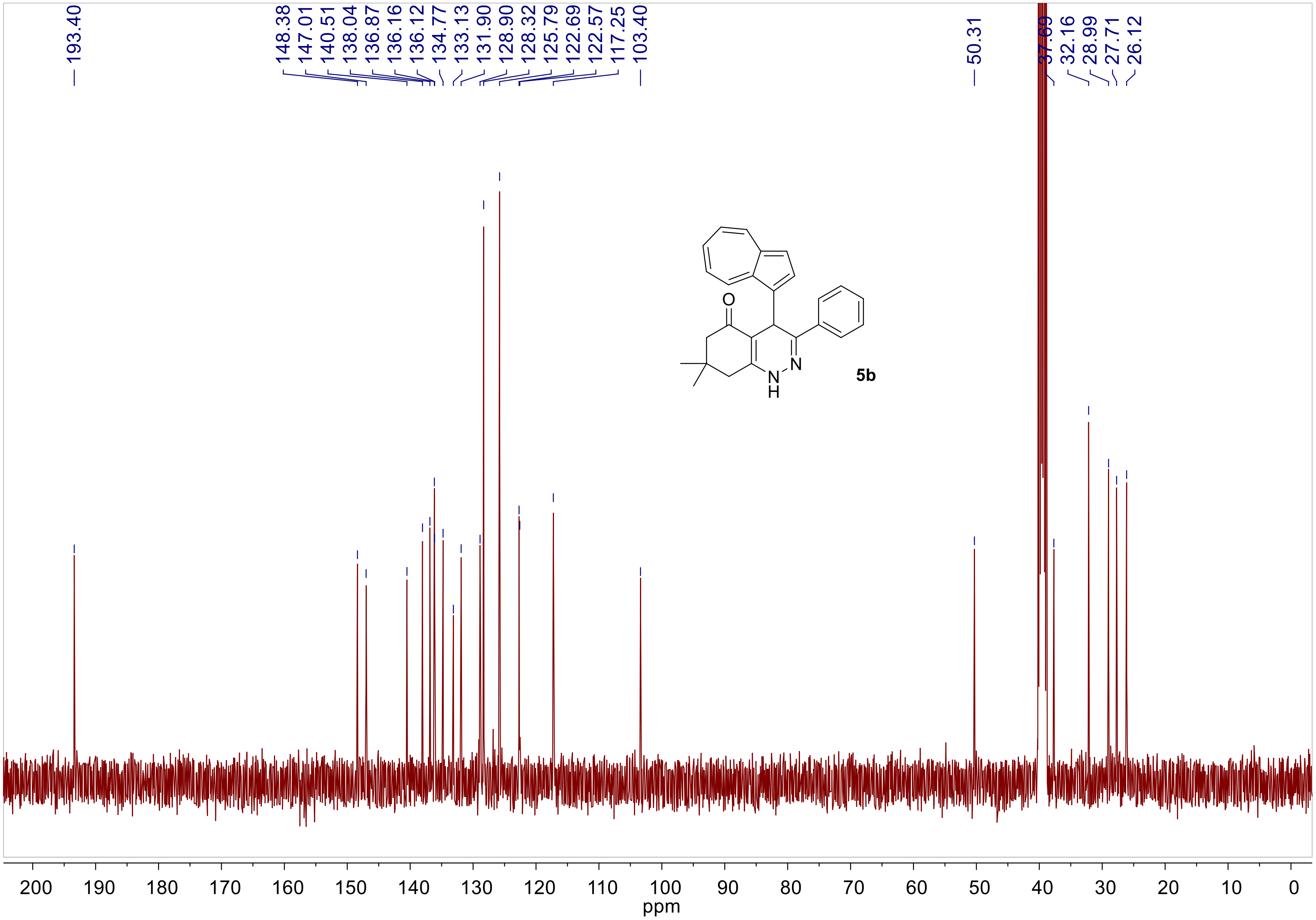
193.40
148.38
147.01
140.51
138.04
136.87
136.16
136.12
134.77
133.13
131.90
128.90
128.32
125.79
122.69
122.57
117.25
103.40
50.31
37.69
32.16
28.99
27.71
26.12
5b
ppm
200
190
180
170
160
150
140
130
120
110
100
90
80
70
60
50
40
30
20
10
0

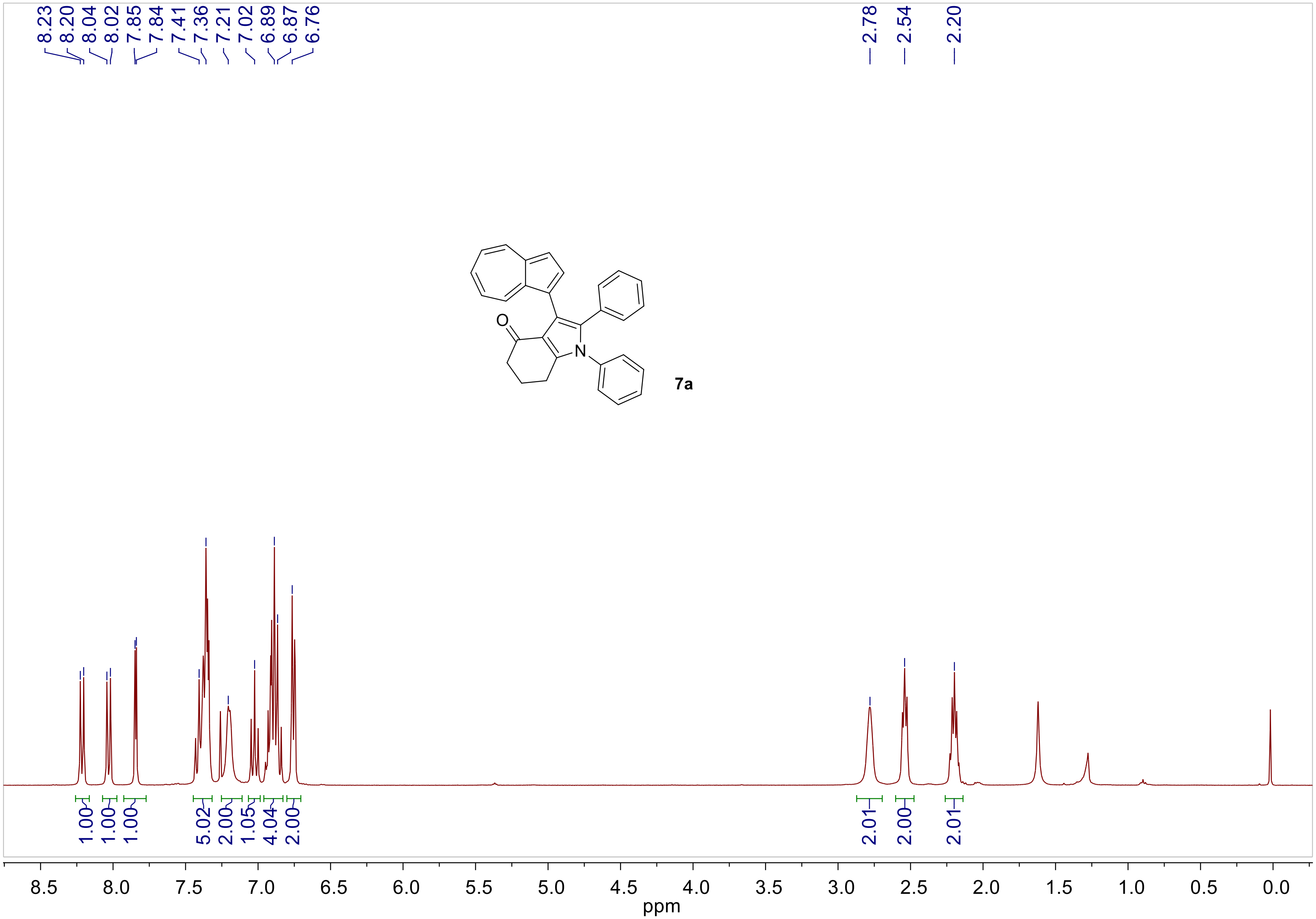
8.23
8.20
8.04
8.02
7.85
7.84
7.41
7.36
7.21
7.02
6.89
6.87
6.76
2.78
2.54
2.20
O
N
7a
1.00
1.00
1.00
5.02
2.00
1.05
4.04
2.00
2.01
2.00
2.01
8.5
8.0
7.5
7.0
6.5
6.0
5.5
5.0
4.5
4.0
3.5
3.0
2.5
2.0
1.5
1.0
0.5
0.0
ppm

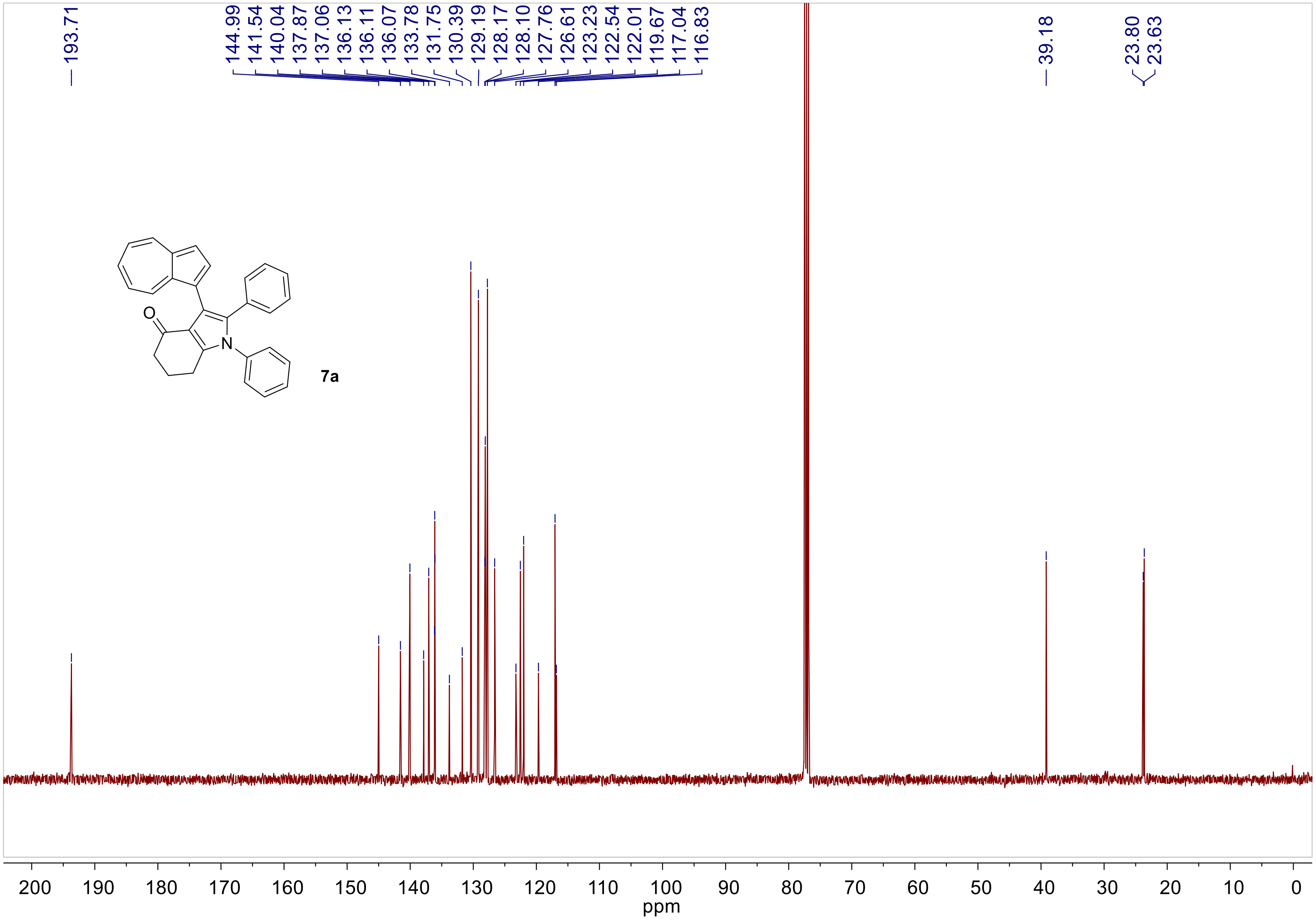

193.71
144.99
141.54
140.04
137.87
137.06
136.13
136.11
136.07
133.78
131.75
130.39
129.19
128.17
128.10
127.76
126.61
123.23
122.54
122.01
119.67
117.04
116.83
39.18
23.80
23.63
O
N
7a
200 190 180 170 160 150 140 130 120 110 100 90 80 70 60 50 40 30 20 10 0
ppm

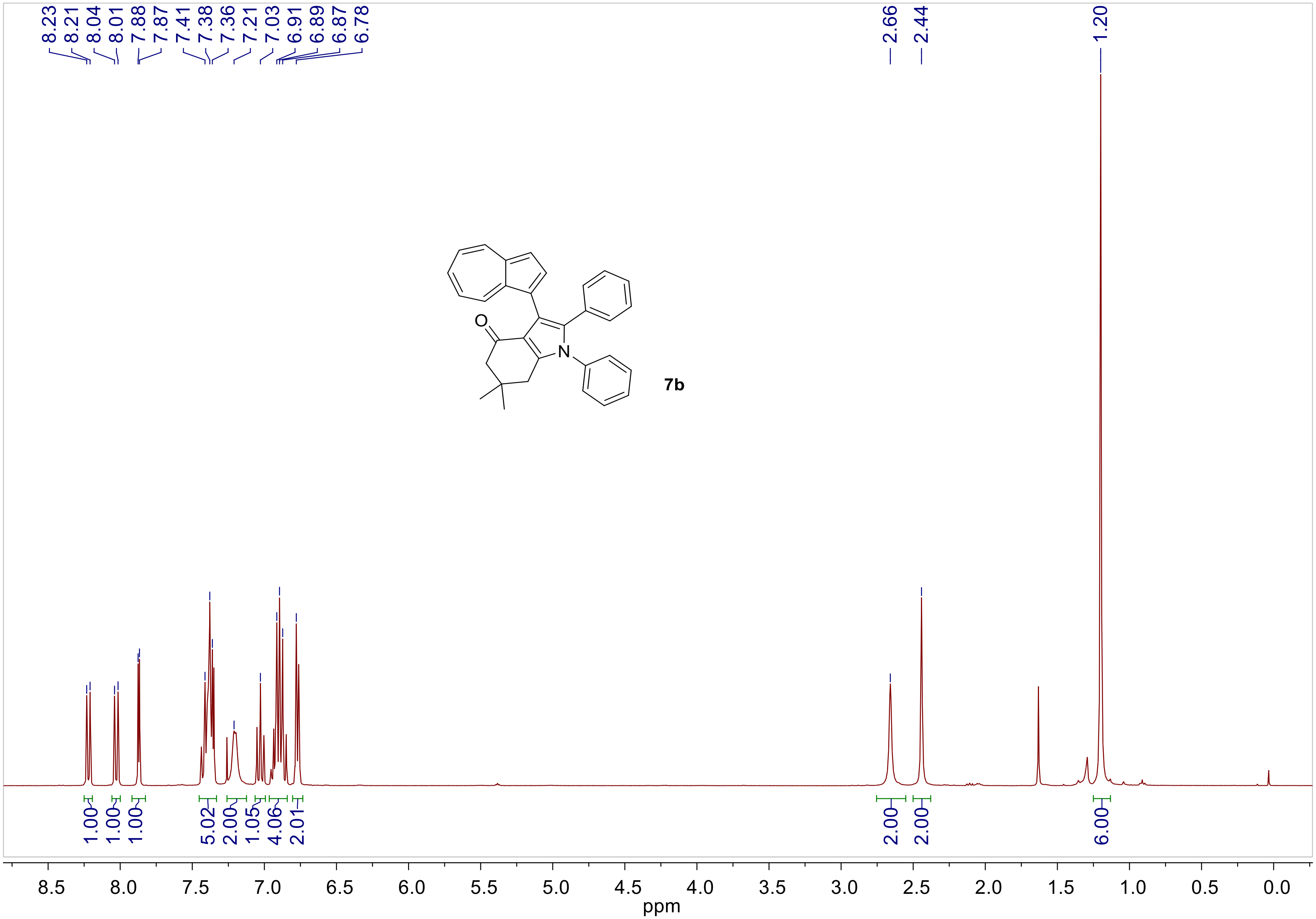
8.23 8.21 8.04 8.01 7.88 7.87 7.41 7.38 7.36 7.21 7.03 6.91 6.89 6.87 6.78
2.66 2.44
1.20
O
N
7b
1.00 1.00 1.00 5.02 2.00 1.05 4.06 2.01
2.00 2.00
6.00
8.5 8.0 7.5 7.0 6.5 6.0 5.5 5.0 4.5 4.0 3.5 3.0 2.5 2.0 1.5 1.0 0.5 0.0
ppm

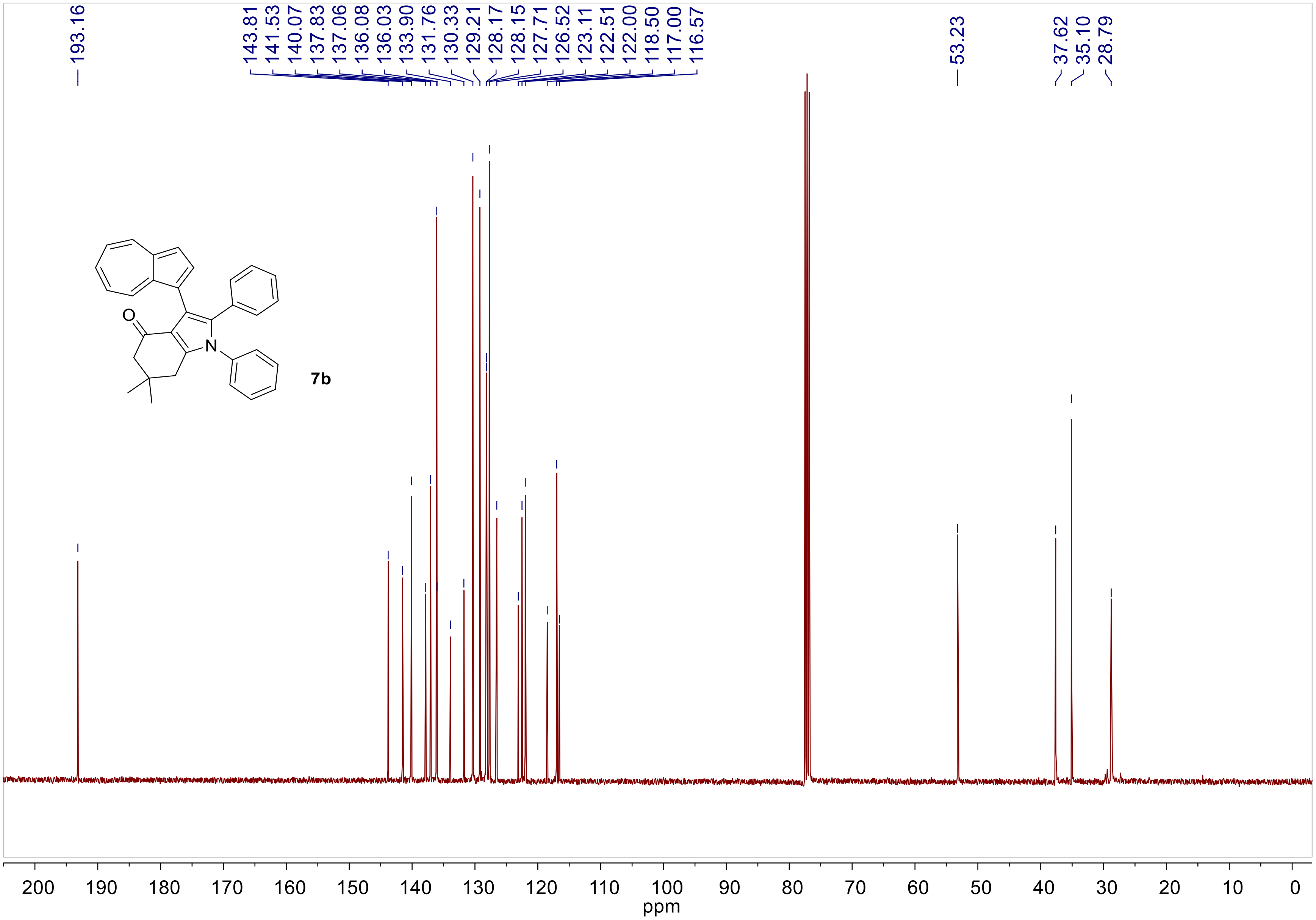
193.16
143.81
141.53
140.07
137.83
137.06
136.08
136.03
133.90
131.76
130.33
129.21
128.17
128.15
127.71
126.52
123.11
122.51
122.00
118.50
117.00
116.57
53.23
37.62
35.10
28.79
O
N
7b
200 190 180 170 160 150 140 130 120 110 100 90 80 70 60 50 40 30 20 10 0
ppm

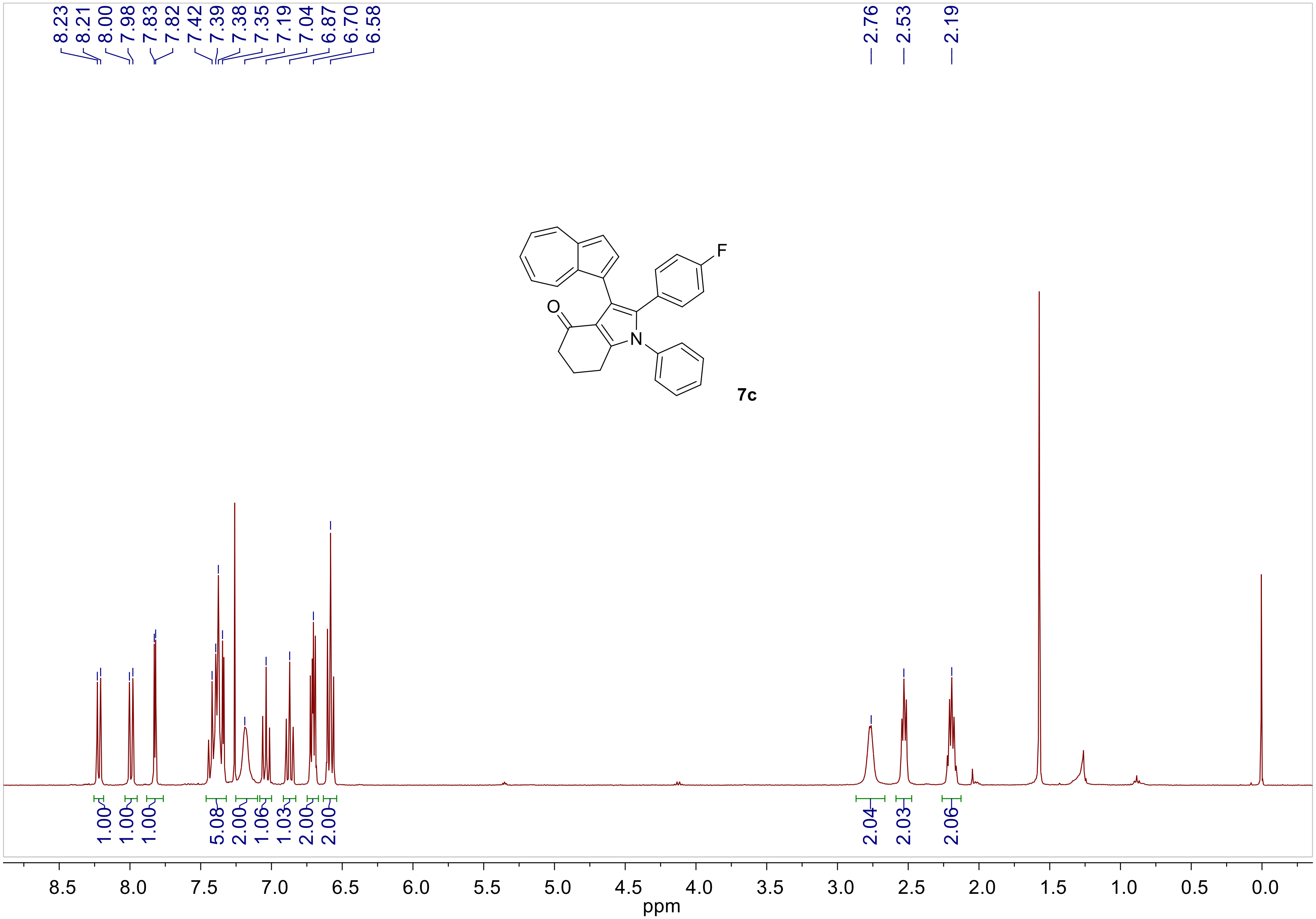

8.23
8.21
8.00
7.98
7.83
7.82
7.42
7.39
7.38
7.35
7.19
7.04
6.87
6.70
6.58
2.76
2.53
2.19
O
N
F
7c
1.00
1.00
1.00
5.08
2.00
1.06
1.03
2.00
2.00
2.04
2.03
2.06
8.5
8.0
7.5
7.0
6.5
6.0
5.5
5.0
4.5
4.0
3.5
3.0
2.5
2.0
1.5
1.0
0.5
0.0
ppm

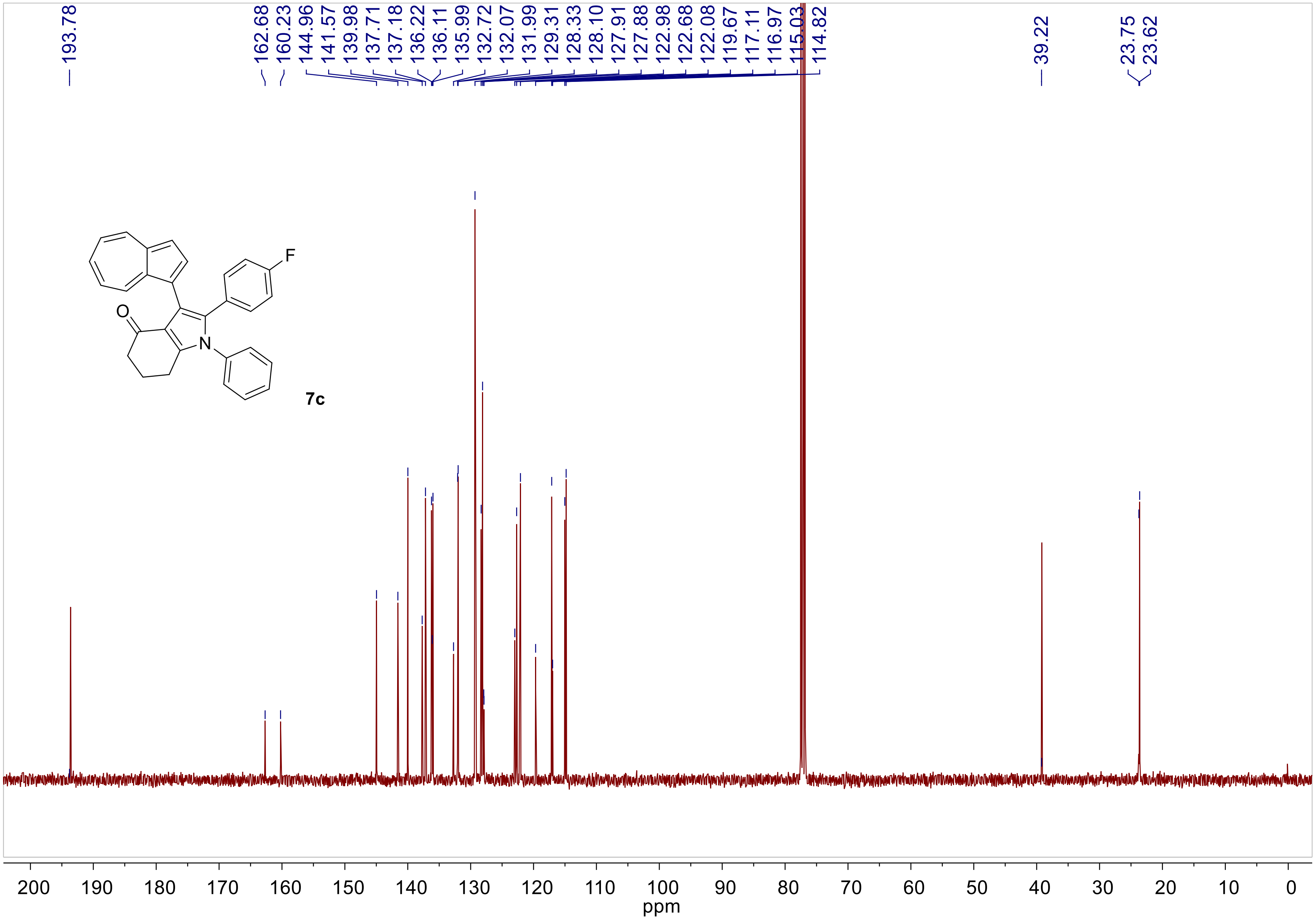
193.78
162.68
160.23
144.96
141.57
139.98
137.71
137.18
136.22
136.11
135.99
132.72
132.07
131.99
129.31
128.33
128.10
127.91
127.88
122.98
122.68
122.08
119.67
117.11
116.97
115.03
114.82
39.22
23.75
23.62
F
O
N
7c
200 190 180 170 160 150 140 130 120 110 100 90 80 70 60 50 40 30 20 10 0
ppm

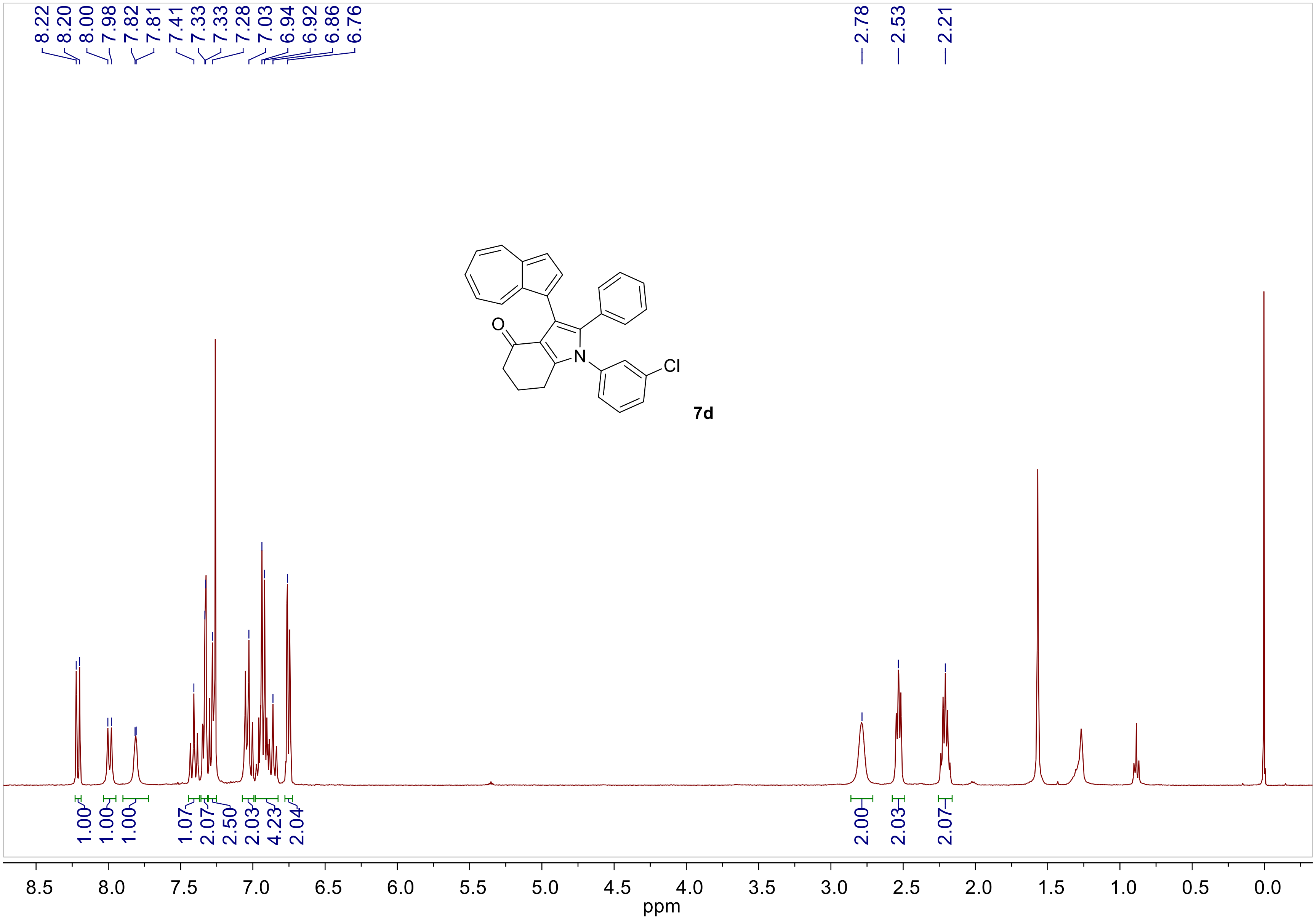
8.22
8.20
8.00
7.98
7.82
7.81
7.41
7.33
7.33
7.28
7.03
6.94
6.92
6.86
6.76
2.78
2.53
2.21
O
N
Cl
7d
1.00
1.00
1.00
1.07
2.07
2.50
2.03
4.23
2.04
2.00
2.03
2.07
8.5
8.0
7.5
7.0
6.5
6.0
5.5
5.0
4.5
4.0
3.5
3.0
2.5
2.0
1.5
1.0
0.5
0.0
ppm

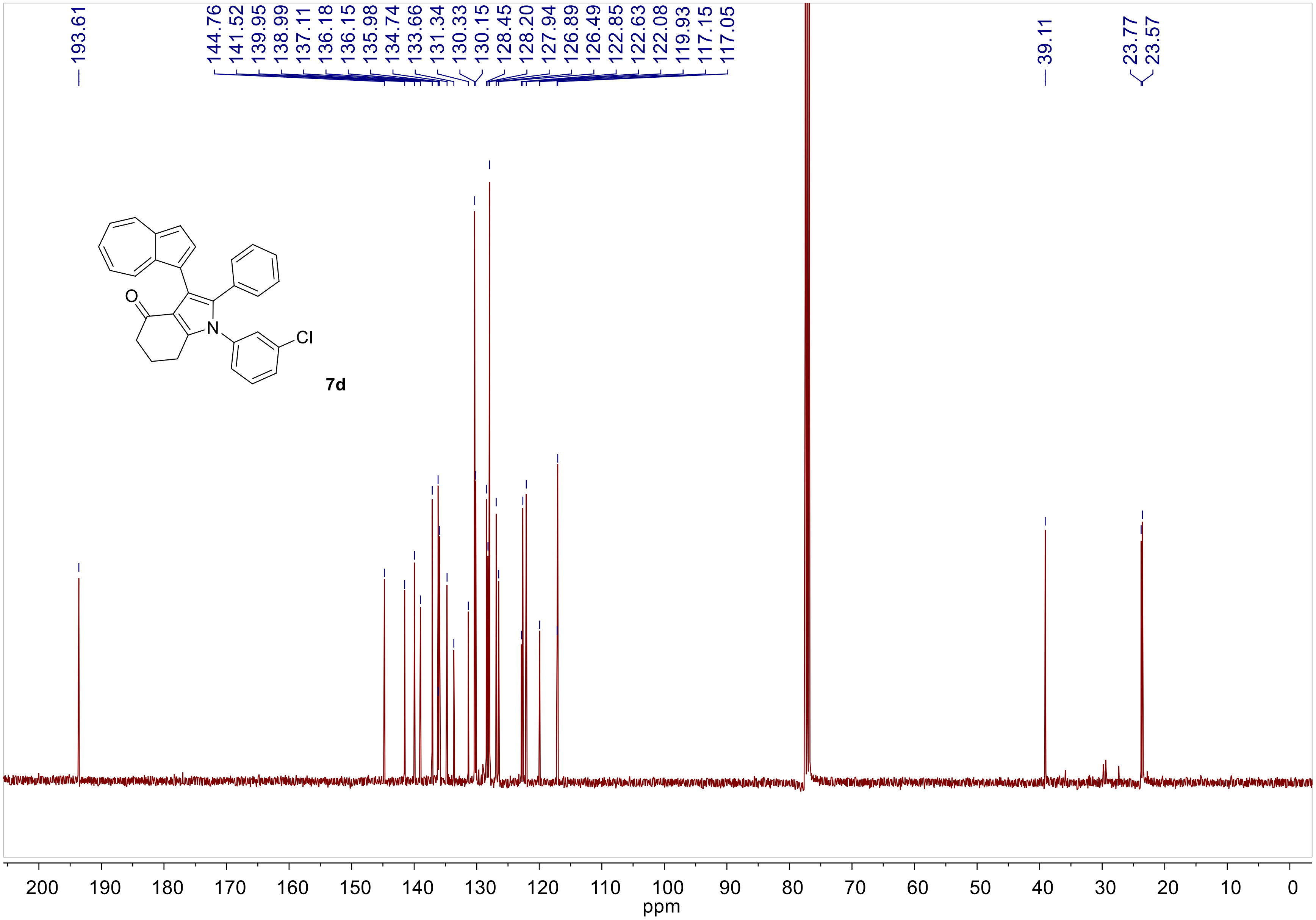
193.61
144.76
141.52
139.95
138.99
137.11
136.18
136.15
135.98
134.74
133.66
131.34
130.33
130.15
128.45
128.20
127.94
126.89
126.49
122.85
122.63
122.08
119.93
117.15
117.05
39.11
23.77
23.57
O
N
Cl
7d
200 190 180 170 160 150 140 130 120 110 100 90 80 70 60 50 40 30 20 10 0
ppm

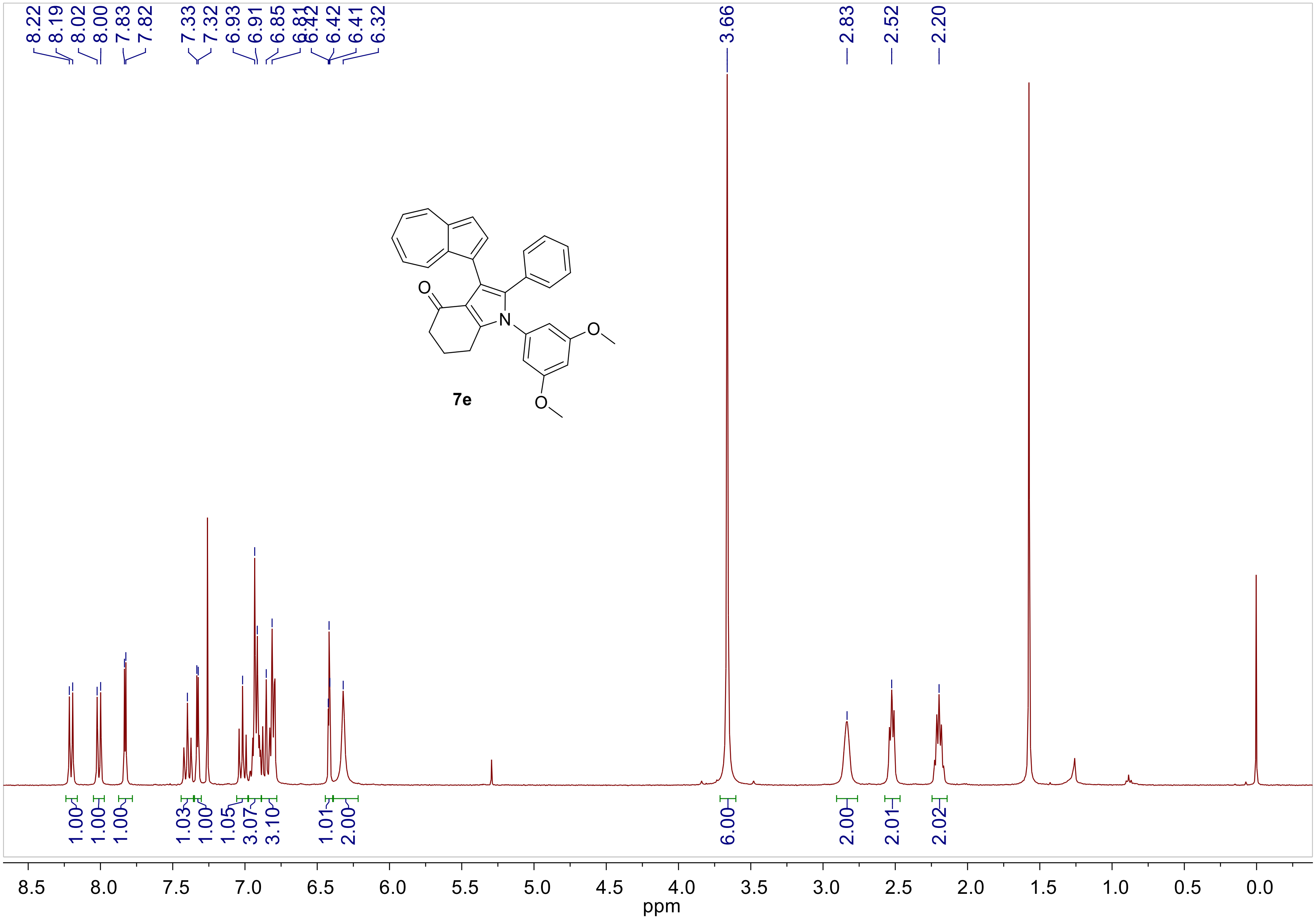
8.22
8.19
8.02
8.00
7.83
7.82
7.33
7.32
6.93
6.91
6.85
6.81
6.42
6.42
6.41
6.32
3.66
2.83
2.52
2.20
O
N
O
O
7e
1.00
1.00
1.00
1.03
1.00
1.05
3.07
3.10
1.01
2.00
6.00
2.00
2.01
2.02
8.5
8.0
7.5
7.0
6.5
6.0
5.5
5.0
4.5
4.0
3.5
3.0
2.5
2.0
1.5
1.0
0.5
0.0
ppm

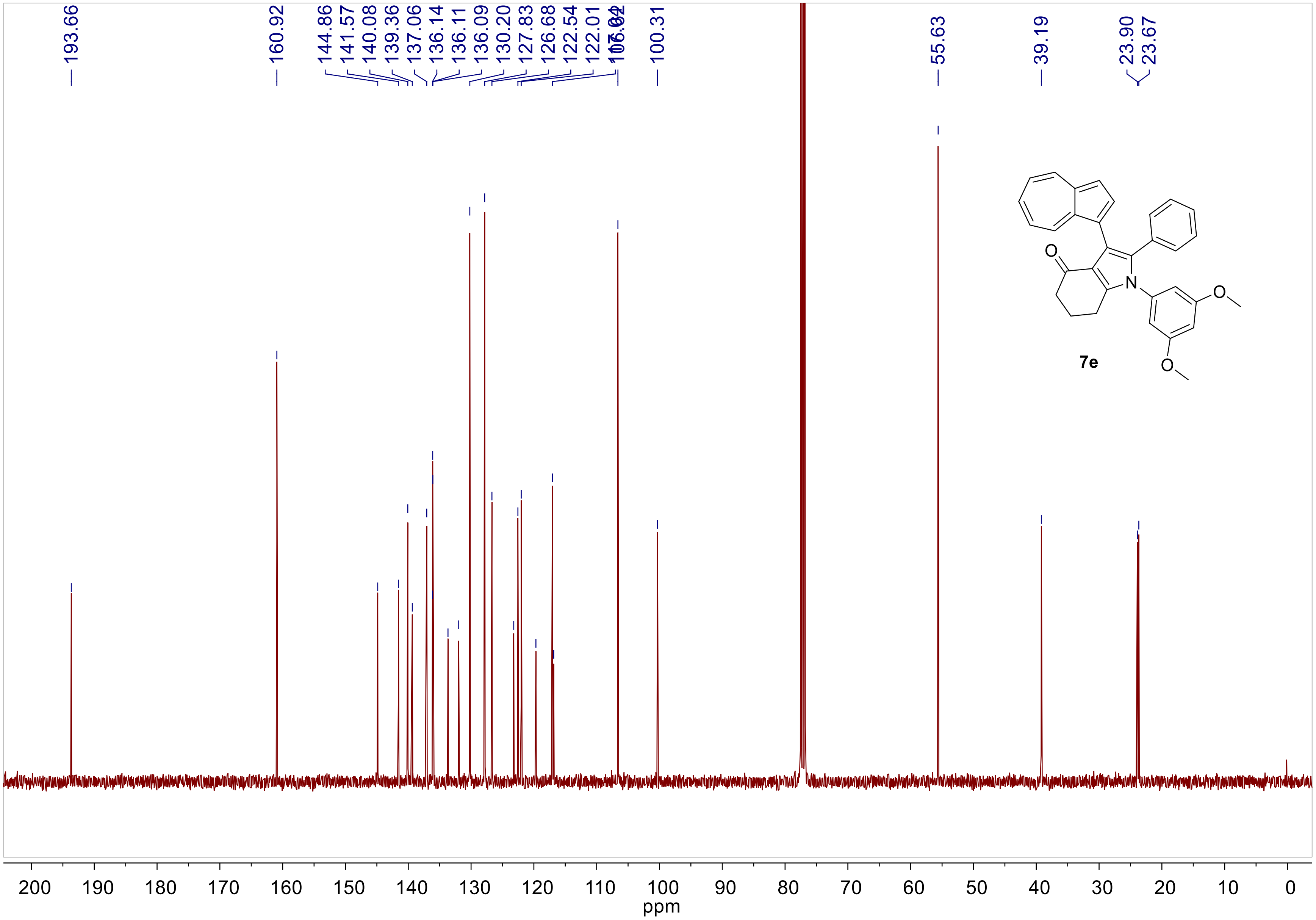
193.66
160.92
144.86
141.57
140.08
139.36
137.06
136.14
136.11
136.09
130.20
127.83
126.68
122.54
122.01
100.31
55.63
39.19
23.90
23.67
O
N
O
O
7e
200 190 180 170 160 150 140 130 120 110 100 90 80 70 60 50 40 30 20 10 0
ppm

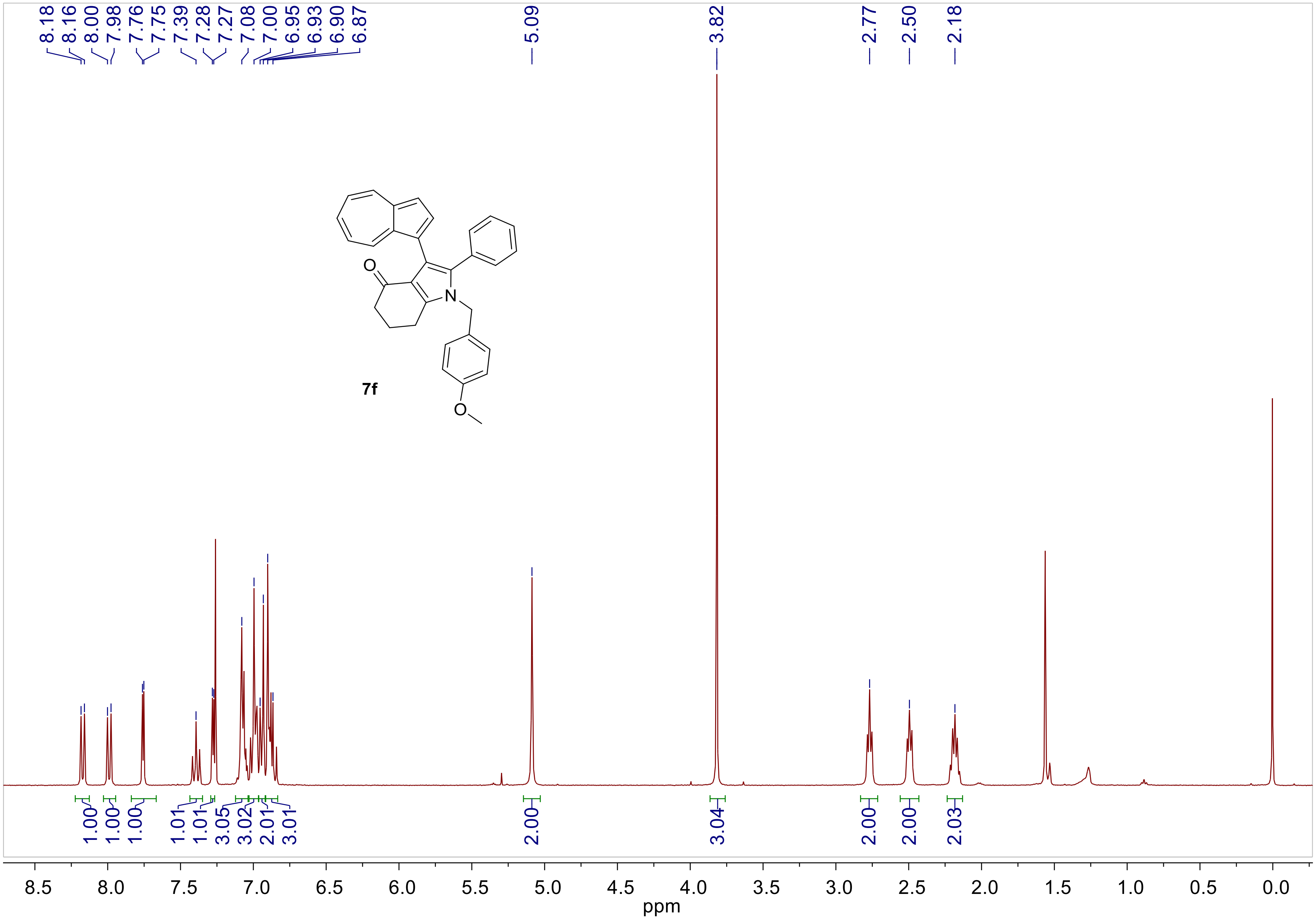
8.18
8.16
8.00
7.98
7.76
7.75
7.39
7.28
7.27
7.08
7.00
6.95
6.93
6.90
6.87
5.09
3.82
2.77
2.50
2.18
O
N
O
7f
1.00
1.00
1.00
1.01
1.01
3.05
3.02
2.01
3.01
2.00
3.04
2.00
2.00
2.03
8.5
8.0
7.5
7.0
6.5
6.0
5.5
5.0
4.5
4.0
3.5
3.0
2.5
2.0
1.5
1.0
0.5
0.0
ppm

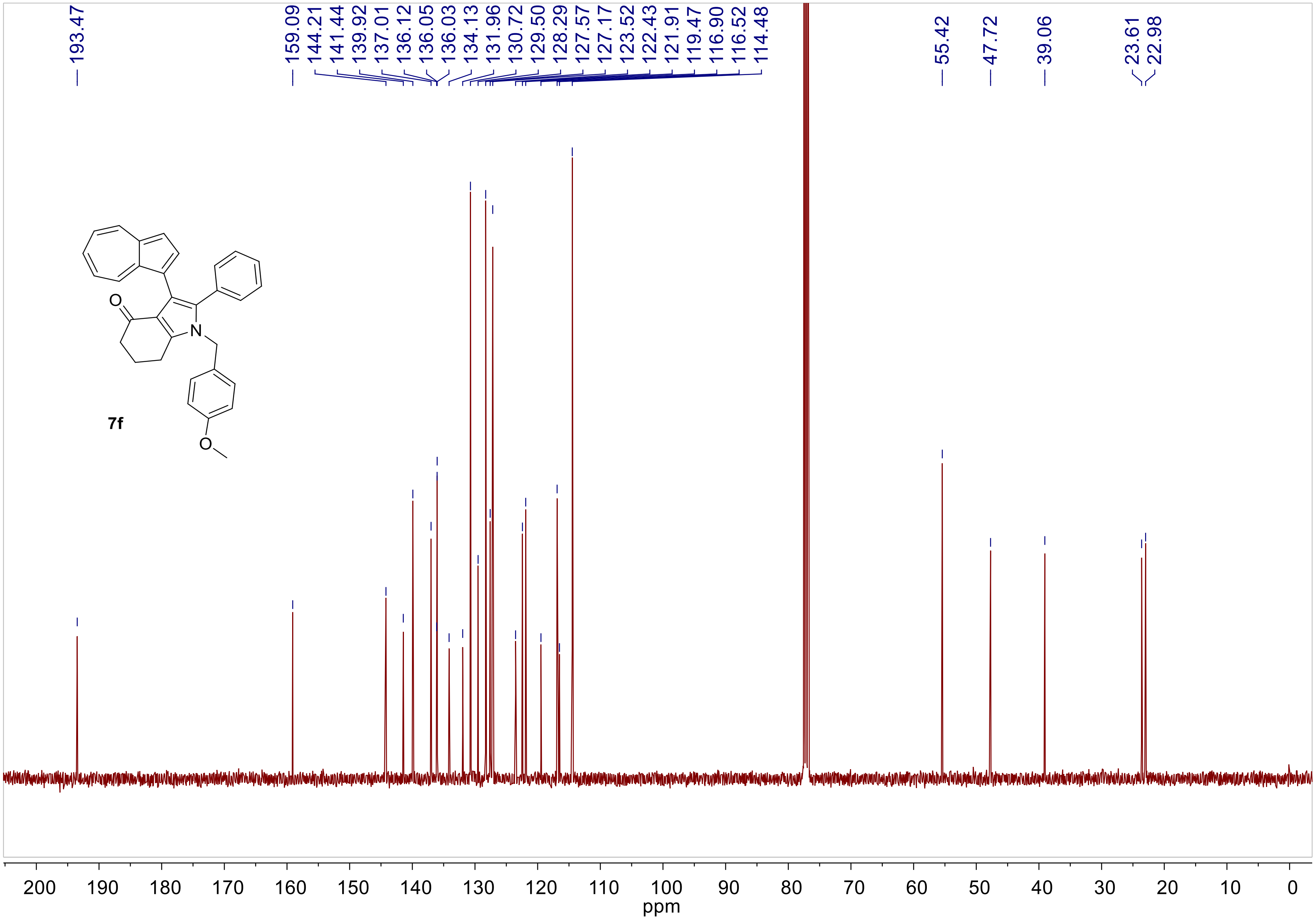
193.47
159.09
144.21
141.44
139.92
137.01
136.12
136.05
136.03
134.13
131.96
130.72
129.50
128.29
127.57
127.17
123.52
122.43
121.91
119.47
116.90
116.52
114.48
55.42
47.72
39.06
23.61
22.98
O
N
O
7f
200
190
180
170
160
150
140
130
120
110
100
90
80
70
60
50
40
30
20
10
0
ppm

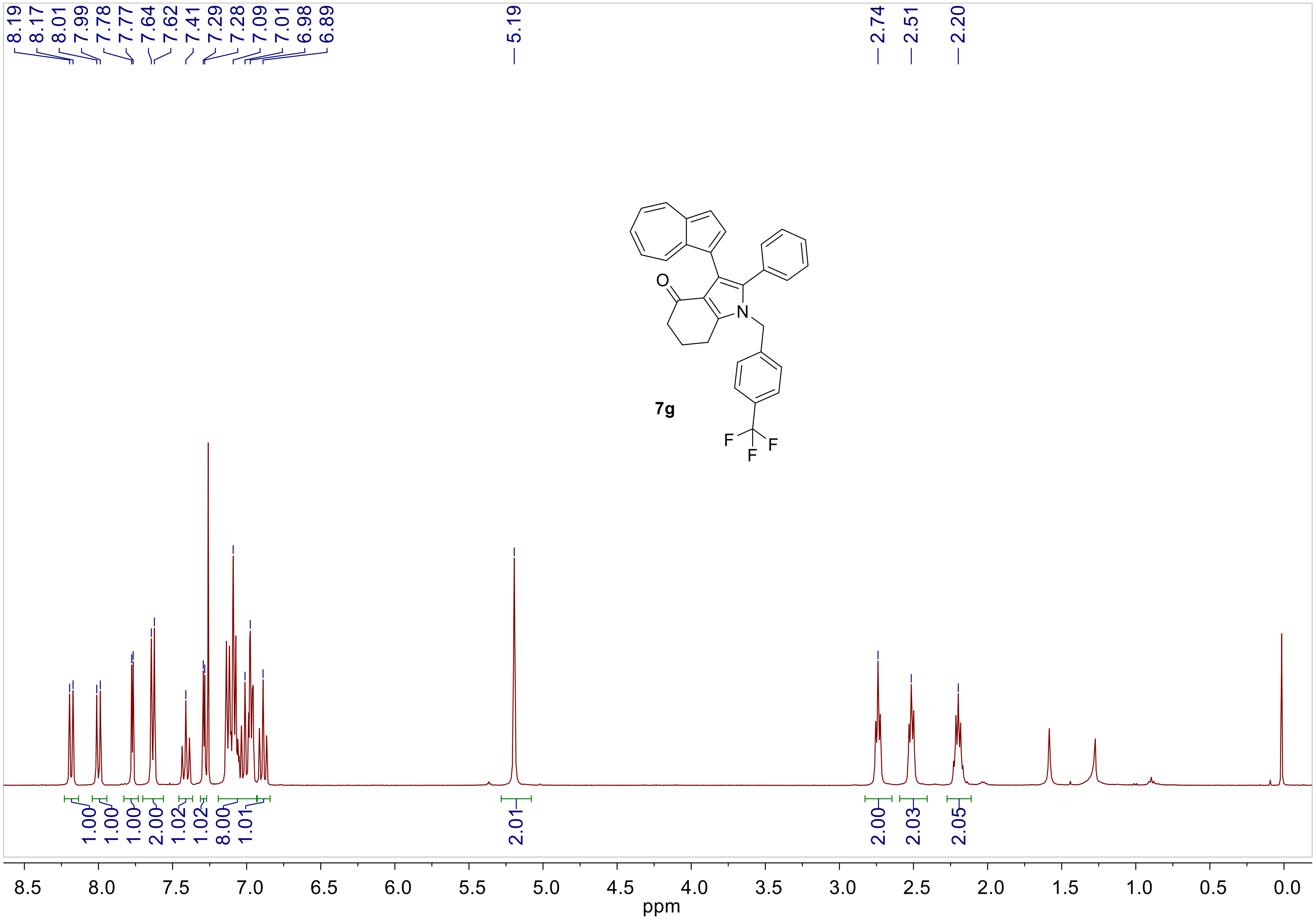
8.19
8.17
8.01
7.99
7.78
7.77
7.64
7.62
7.41
7.29
7.28
7.09
7.01
6.98
6.89
5.19
2.74
2.51
2.20
O
N
7g
F
F
F
1.00
1.00
1.00
2.00
1.02
1.02
8.00
1.01
2.01
2.00
2.03
2.05
8.5
8.0
7.5
7.0
6.5
6.0
5.5
5.0
4.5
4.0
3.5
3.0
2.5
2.0
1.5
1.0
0.5
0.0
ppm

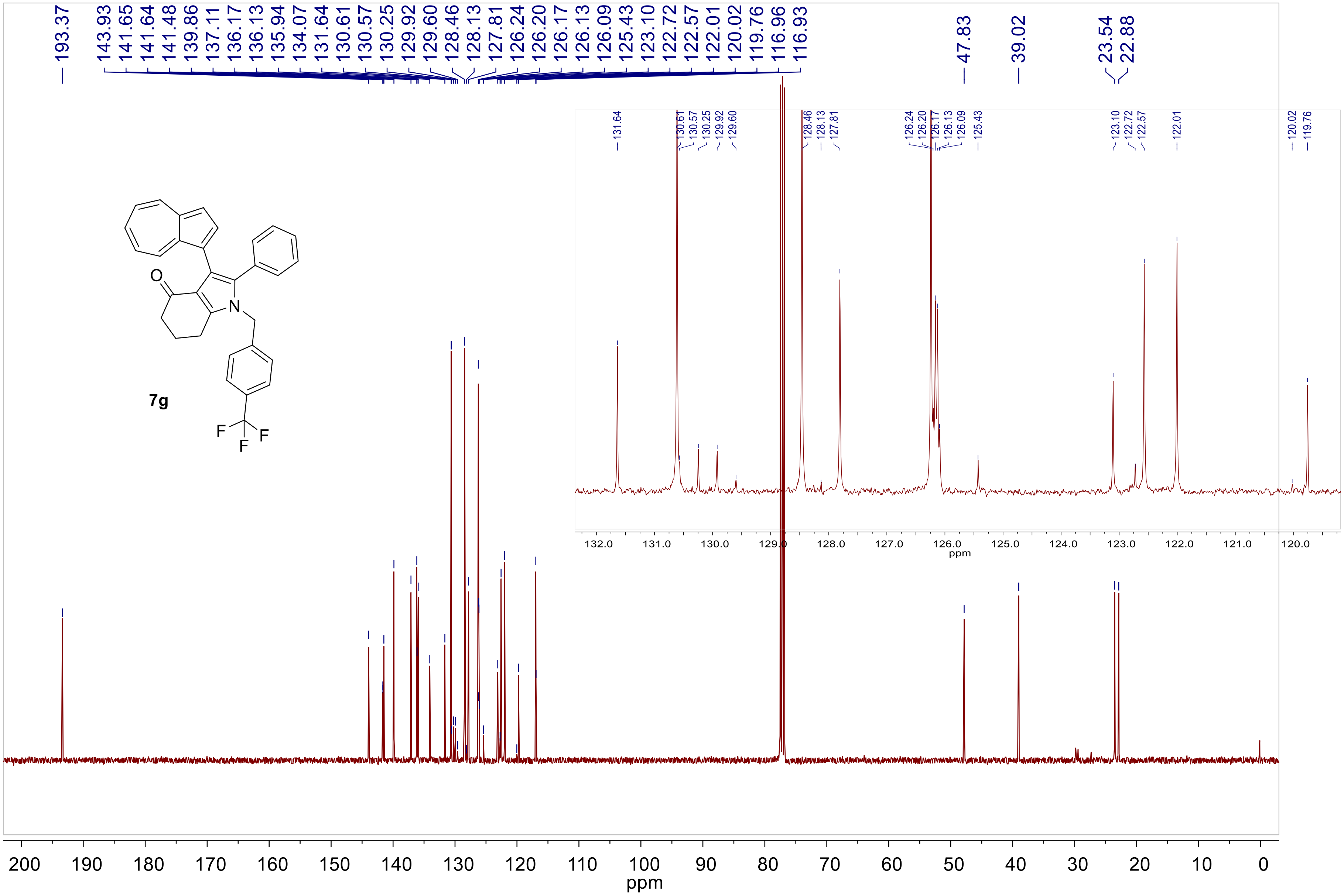
193.37
143.93
141.65
141.64
141.48
139.86
137.11
136.17
136.13
135.94
134.07
131.64
130.61
130.57
130.25
129.92
129.60
128.46
128.13
127.81
126.24
126.20
126.17
126.13
126.09
125.43
123.10
122.72
122.57
122.01
120.02
119.76
116.96
116.93
47.83
39.02
23.54
22.88
O
N
F
F
F
7g
ppm
ppm

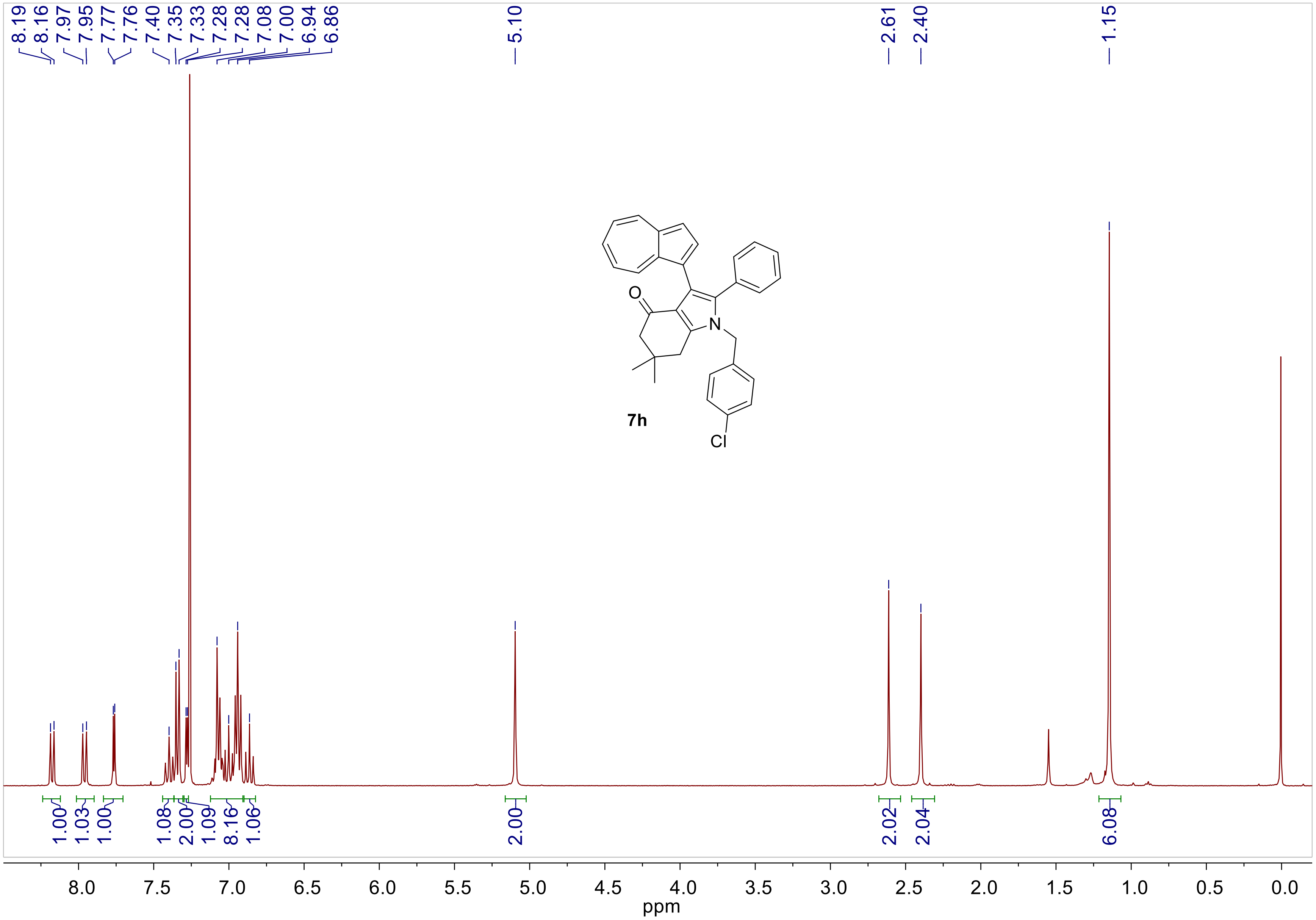
8.19
8.16
7.97
7.95
7.77
7.76
7.40
7.35
7.33
7.28
7.28
7.08
7.00
6.94
6.86
5.10
2.61
2.40
1.15
O
N
Cl
7h
1.00
1.03
1.00
1.08
2.00
1.09
8.16
1.06
2.00
2.02
2.04
6.08
8.0
7.5
7.0
6.5
6.0
5.5
5.0
4.5
4.0
3.5
3.0
2.5
2.0
1.5
1.0
0.5
0.0
ppm

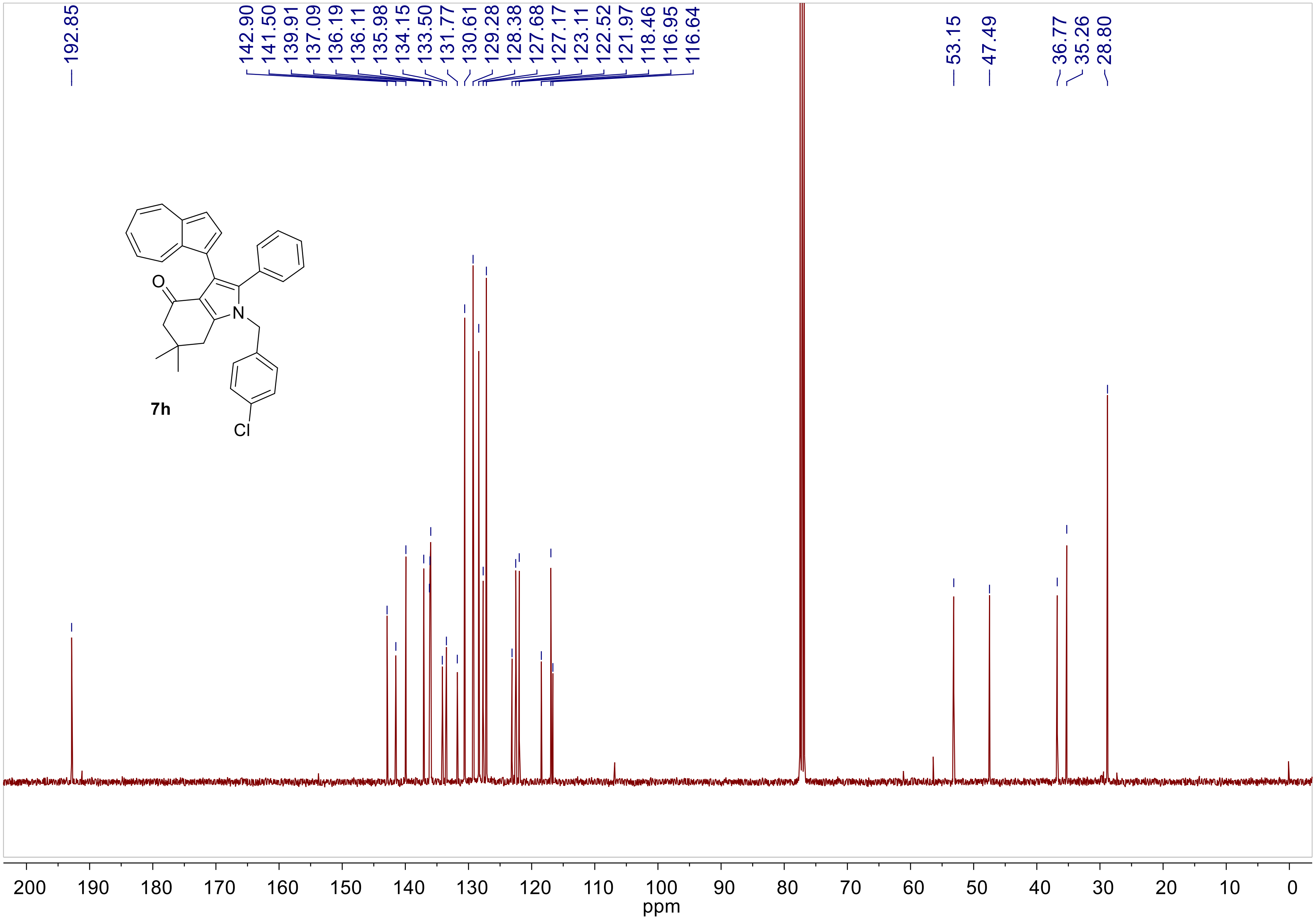
192.85
142.90
141.50
139.91
137.09
136.19
136.11
135.98
134.15
133.50
131.77
130.61
129.28
128.38
127.68
127.17
123.11
122.52
121.97
118.46
116.95
116.64
53.15
47.49
36.77
35.26
28.80
O
N
Cl
7h
200 190 180 170 160 150 140 130 120 110 100 90 80 70 60 50 40 30 20 10 0
ppm
